# Supplementary material for: Phospholipid scramblase Xkr8 is required for developmental axon pruning via phosphatidylserine exposure
Source: EMBO J. 2023 May 22;42(14):e111790. doi: 10.15252/embj.2022111790 (PMC10350823; doi:10.15252/embj.2022111790)
Supplement: Supplementary file 1 — Appendix [file EMBJ-42-e111790-s003.pdf]

# Phospholipid scramblase Xkr8 is required for developmental axon pruning

Urte Neniskyte*, Ugne Kuliesiute, Auguste Vadisiute, Kristina Jevdokimenko, Ludovico Coletta, Senthilkumar Deivasigamani, Daina Pamedytyte, Neringa Daugelaviciene, Daiva Dabkeviciene, Emerald Perlas, Daina Pamedytyte, Ugne Kuliesiute, Aditya Bali, Bernadette Basilico, Alessandro Gozzi, Davide Ragozzino, Cornelius T. Gross*

## Appendix

Table of contents

1. Appendix Figure S1 page 2
2. Appendix Figure S2 page 3
3. Appendix Table S1 page 4
4. Appendix Figure S3 page 6
5. Appendix Figure S4 page 7
6. Appendix Figure S5 page 8

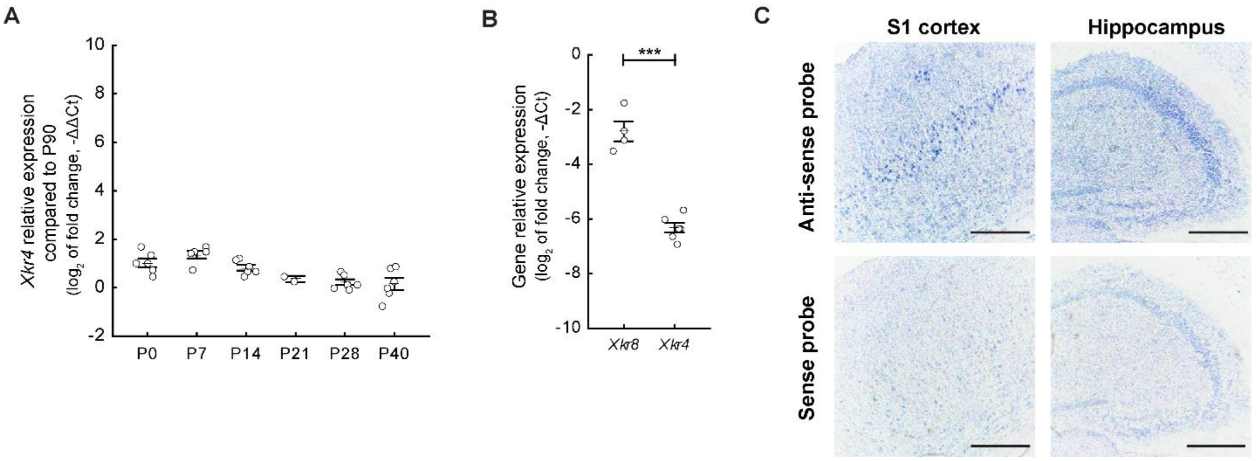


**Appendix Figure S1. Expression of *Xkr8* and *Xkr4* in developing brain. A** Developmental profile of *Xkr4* mRNA expression in mouse brain from P0 to P40 was measured by quantitative RT-PCR and normalized to the expression of *Gapdh* at a relative time-point and to the expression of the gene of interest at P90. No significant changes in the expression of *Xkr4* were observed during the course of development. (one-way ANOVA, each dot represents an individual mouse, $n$ = 2–6 per age group; mean ± SEM).

**B** Expression of *Xkr8* and *Xkr4* mRNA in postnatal P0 brain by quantitative RT-PCR normalized to the expression of *Gadph* at P0. Significantly higher expression of *Xkr8* was observed in P0 brains compared to *Xkr4* (one-way ANOVA, each dot represents an individual mouse, $n$ = 2–6 per age group; mean ± SEM, *** $p < 0.001$).

**C** *Xkr8* mRNA expression in hippocampus and cortex of adult mice visualized by *in situ* hybridization; *scale bar* 200 µm.

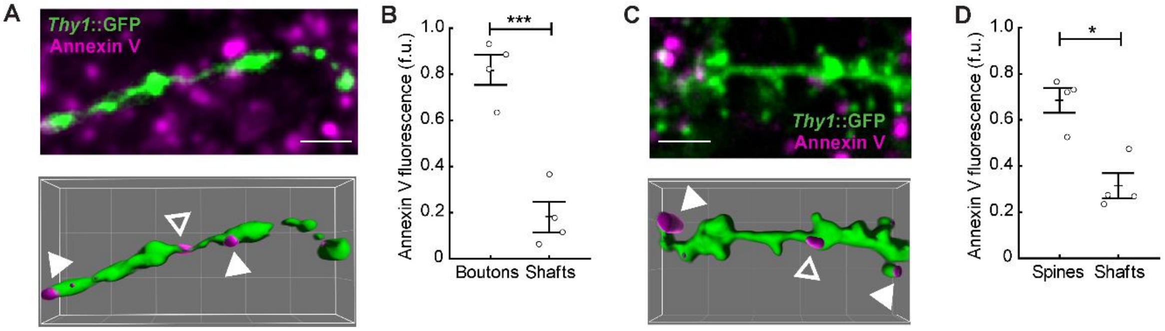


**Appendix Figure S2. PtdSer is preferentially exposed on synaptic structures of pyramidal *Thy1*::GFP neurons.**

**A-D** Organotypic hippocampal slices from *Thy1*::GFP (*green*) mice were labelled with PtdSer-binding fluorescently tagged Annexin V (*magenta*) at 16-19 DIV. Fluorescent confocal image *z* stacks were reconstructed on Imaris to obtain 3D surfaces of axons (**A**) and dendrites (**C**); *scale bar* 2 µm, *grid cell* 2 µm. The fluorescence of bound Annexin V within GFP+ was quantified on ImageJ by measuring the integrated density of fluorescent structures within *Thy1*::GFP surface. GFP+ axonal boutons (**B**) and dendritic shafts (**D**) were identified manually by their characteristic structure. Annexin V structures on boutons or spines are labelled with closed arrows, while those on shafts are labelled with open arrows. The data were compared by two-tailed Student's *t*-test, each dot represents OHSC preparation from one mouse, n = 4; mean ± SEM, * $p < 0.05$, *** $p < 0.001$.

**Appendix Table S1. Statistical analysis of data presented in Figure 1–3 using sex as an independent variable.** (NOTE: Due to small sample sizes, these results are only indicative.)

| Two-way/Three-way ANOVA | | | | | Mean±SEM; n | |
|---|---|---|---|---|---|---|
| Figure | Effect | Degr. of freedom | F | *p* | Male | Female |
| 1a | SEX | 1, 13 | 0.61 | 0.45 | 2.17±0.79; 13 | 2.79±0.89; 12 |
| | AGE*SEX: | 5, 13 | 0.34 | 0.88 | | |
| | P0 | | | | 5.87±2.13; 2 | 8.84±0.49; 2 |
| | P7 | | | | 2.48±2.58; 3 | 1.92±1.03; 2 |
| | P14 | | | | 2.07±1.04; 3 | 2.61±1.83; 2 |
| | P21 | | | | 1.20±NA; 1 | 1.22±0.11; 2 |
| | P28 | | | | 0.20±0.17; 2 | 1.72±0.80; 2 |
| | P40 | | | | 0.58±0.03; 2 | 0.46±0.05; 2 |
| 1g | SEX | 1, 26 | 1.09 | 0.31 | 6.3±1.01; 18 | 8.04±1.51; 18 |
| | AGE*SEX: | 2, 26 | 0.80 | 0.46 | | |
| | P8 | | | | 8.72±2.51; 6 | 13.17±3.48; 6 |
| | P15 | | | | 5.35±1.08; 6 | 6.50±1.49; 6 |
| | P28 | | | | 4.95±1.09; 6 | 4.45±0.87; 6 |
| 2c | SEX | 1, 26 | 1.09 | 0.31 | 6.3±1.01; 18 | 8.04±1.51; 18 |
| | AGE*SEX: | 2, 26 | 0.80 | 0.46 | | |
| | P8 | | | | 8.72±2.51; 6 | 13.17±3.48; 6 |
| | P15 | | | | 5.35±1.08; 6 | 6.50±1.49; 6 |
| | P28 | | | | 4.95±1.09; 6 | 4.45±0.87; 6 |
| | GENE*SEX: | 1, 26 | 0.20 | 0.66 | | |
| | WT | | | | 6.93±1.40; 9 | 9.34±1.22; 9 |
| | cKO | | | | 5.76±1.51; 9 | 6.74±2.79; 9 |
| 3b | SEX | 1, 26 | 11.59 | **0.002** | **0.13±0.006; 18** | **0.11±0.004; 18** |
| | AGE*SEX: | 2, 26 | 1.82 | 0.18 | | |
| | P8 | | | | 0.15±0.006; 6 | 0.12±0.007; 6 |
| | P15 | | | | 0.12±0.008; 6 | 0.11±0.005; 6 |
| | P28 | | | | 0.13±0.011; 6 | 0.10±0.007; 6 |
| | GENE*SEX: | 1, 26 | 0.01 | 0.93 | | |
| | WT | | | | 0.12±0.009; 9 | 0.10±0.006; 9 |
| | cKO | | | | 0.14±0.007; 9 | 0.12±0.004; 9 |
| 3c | SEX | 1, 26 | 5.08 | **0.03** | **2.96±0.12; 18** | **2.66±0.09; 18** |
| | AGE*SEX: | 2, 26 | 0.76 | 0.48 | | |
| | P8 | | | | 3.39±0.06; 6 | 2.91±0.11; 6 |
| | P15 | | | | 2.72±0.21; 6 | 2.37±0.15; 6 |
| | P28 | | | | 2.78±0.23; 6 | 2.69±0.11; 6 |
| | GENE*SEX: | 1, 26 | 0.49 | 0.49 | | |
| | Wt | | | | 2.93±0.17; 9 | 2.72±0.16; 9 |
| | cKO | | | | 2.99±0.18; 9 | 2.60±0.07; 9 |

*Continued on the next page*

*Continued from the last page*

| | | | | | | |
|---|---|---|---|---|---|---|
| 3e | SEX<br>AGE*SEX:<br>P8<br>P15<br>P28<br>GENE*SEX:<br>WT<br>cKO | 1, 26<br>2, 26<br><br><br><br>1, 26 | 2.41<br>3.23<br><br><br><br>0.11 | 0.13<br>0.06<br><br><br><br>0.78 | 13.29±1.63; 18<br><br>3.97±0.51; 6<br>17.07±0.36; 6<br>18.83±0.57; 6<br><br>13.04±2.38; 9<br>13.54±2.36; 9 | 12.69±1.83; 18<br><br>2.25±0.44; 6<br>16.34±0.36; 6<br>19.49±0.47; 6<br><br>12.56±2.50; 9<br>12.83±2.83; 9 |
| 3f | SEX<br>GENE*SEX:<br>WT<br>cKO | 1, 8<br>1, 8 | 0.72<br>0.01 | 0.42<br>0.93 | 1059±59; 6<br><br>1002±70; 3<br>1116±96; 3 | 980±68; 6<br><br>932±58; 3<br>1029±131; 3 |
| 3j | SEX<br>AGE*SEX:<br>P8<br>P28<br>GENE*SEX:<br>WT<br>cKO | 1, 17<br>1, 17<br><br><br>1, 17 | 0.58<br>4.75<br><br><br>2.53 | 0.46<br>**0.04**<br><br><br>0.13 | 48.1±6.4; 12<br><br>**33.1±9.6; 6**<br>**63.0±1.4; 6**<br><br>39.7±11.6; 6<br>56.4±4.5; 6 | 52.3±6.6; 12<br><br>**49.5±12.1; 6**<br>**55.1±6.6; 6**<br><br>35.1±8.6; 6<br>69.4±1.7; 6 |
| 3k | SEX<br>AGE*SEX:<br>P8<br>P28<br>GENE*SEX:<br>WT<br>cKO | 1, 17<br>1, 17<br><br><br>1, 17 | 0.04<br>2.46<br><br><br>2.86 | 0.84<br>0.14<br><br><br>0.11 | 23.9±4.1; 12<br><br>15.7±3.3; 6<br>32.1±6.3; 6<br><br>27.5±8.1;6<br>20.3±2.5; 6 | 24.7±2.5; 12<br><br>22.7±5.0; 6<br>26.8±1.4; 6<br><br>21.7±4.6; 6<br>27.8±1.7; 6 |
| 3l | SEX<br>AGE*SEX:<br>P8<br>P28<br>GENE*SEX:<br>WT<br>cKO | 1, 17<br>1, 17<br><br><br>1, 17 | 3.16<br>0.48<br><br><br>6.86 | 0.09<br>0.50<br><br><br>**0.02** | 2.52±0.44;12<br><br>2.32±0.66; 6<br>2.72±0.65; 6<br><br>**1.25±0.16; 6**<br>**3.78±0.45; 6** | 2.02±0.19; 6<br><br>2.01±0.31; 6<br>2.03±0.27; 6<br><br>**1.49±0.16; 6**<br>**2.56±0.18; 6** |

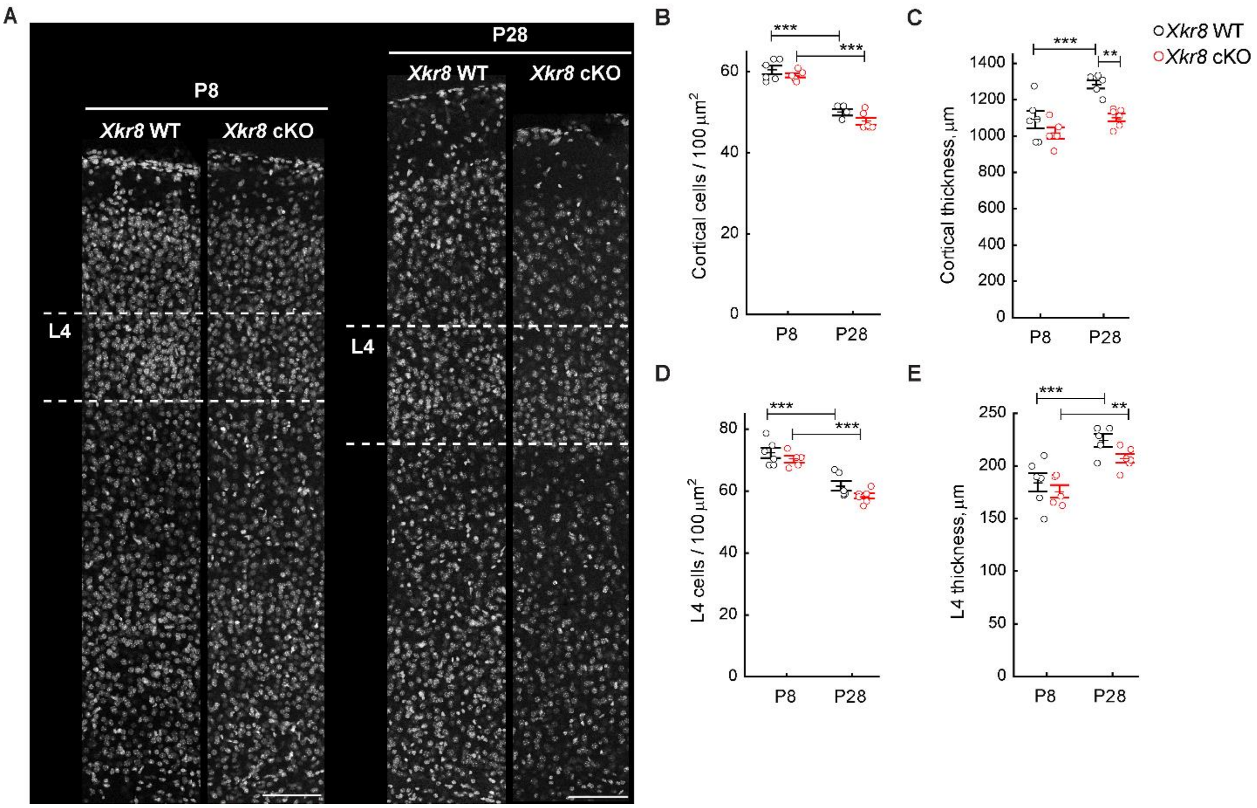


**Appendix Figure S3. No decrease in developmental cell loss in *Xkr8* knockout.**
**A** Cortex of *Xkr8* WT and cKO mice was labelled with DAPI to identify nuclei; *scale bar* 100 µm.
**B-E** (B, D) Quantification of cortical and layer 4 (L4) cell density revealed no difference between *Xkr8* WT and cKO animals. (C, E) Cortical thickness and layer 4 (L4) thickness did not increase in *Xkr8* cKO animals (nested design and mixed model ANOVA, each dot represents an individual mouse, $n$ = 4–6 per age and genotype group; mean ± SEM, ** $p < 0.01$, *** $p < 0.001$).

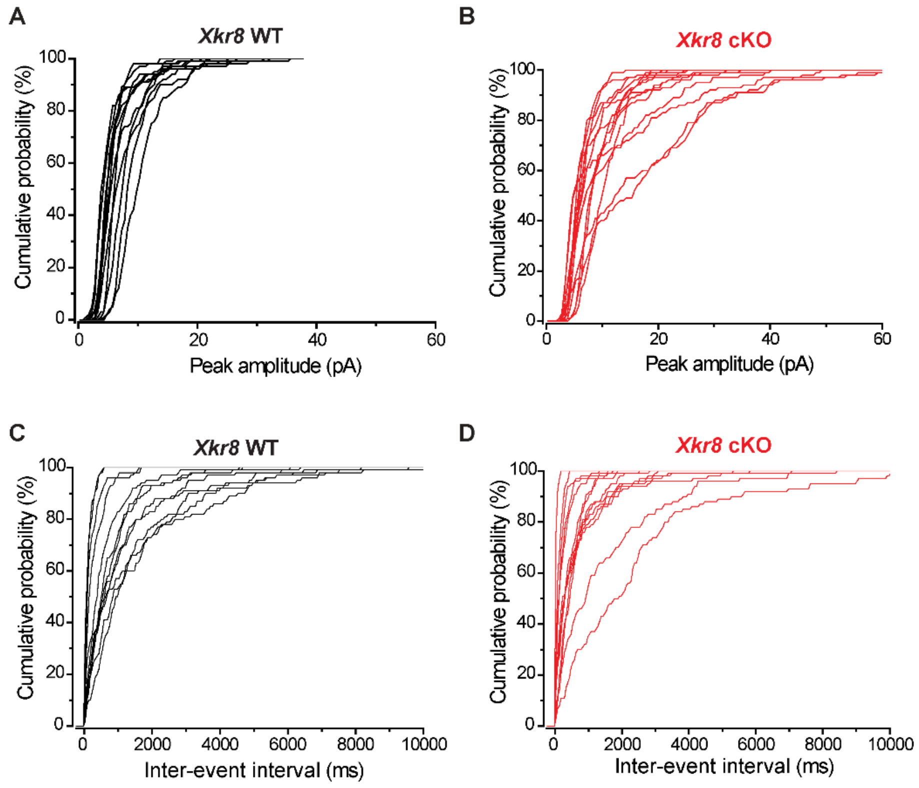


**Appendix Figure S4. Synaptic activity of individual neurons in *Xkr8* cKO and WT hippocampus.**

**A-D** (A, B) Individual traces of sEPSC peak amplitudes of *Xkr8* WT and cKO neurons. (C, D) Individual traces of sEPSC inter-event intervals of *Xkr8* WT and cKO neurons. A subset of *Xkr8* cKO neurons with particularly high synaptic activity (B, D) compared to *Xkr8* WT neurons (A, C) was observed.

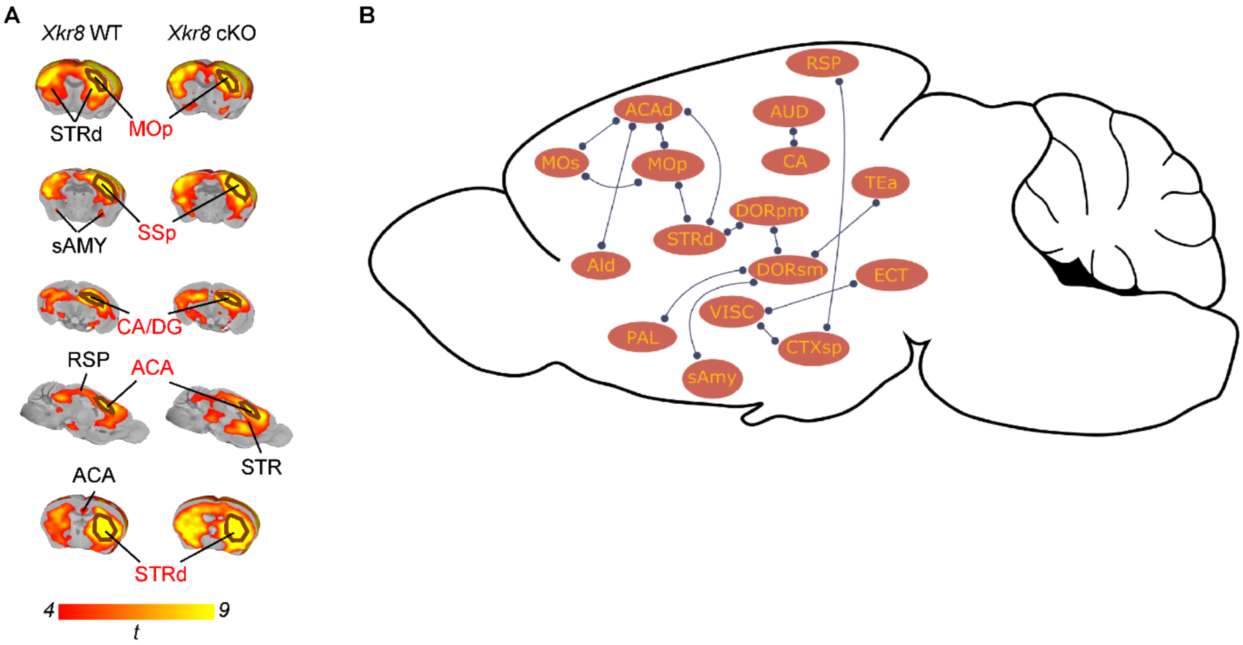


**Appendix Figure S5. Seed-based correlation maps of functional connectivity in *Xkr8* WT and cKO mice.**

**A** Seed-based correlation maps in control and *Xkr8* cKO mice (seed area delineated in black) with red/yellow heat-maps indicating regions exhibiting significant rsfMRI connectivity with the seed volume. The corresponding between-group voxel-wise differences have been reported in **Figure 7**.

**B** Inter-group comparison of intra-hemispheric rsfMRI connectivity in regionally-parcellated brains using network-based statistics (NBS) showed regions exhibiting increased intra-hemispheric connectivity ($t > 2.1$, FWE corrected at $p < 0.05$). Only pairs of regions characterized by significantly increased intra-hemispheric connectivity independently in both hemispheres were retained for display.

Data information: ACA: anterior cingulate cortex, ACAd: anterior cingulate cortex, dorsal, AId: agranular insular area, dorsal, sAMY: striatum-like amygdalar nuclei, AUD: auditory areas, CA: Ammon's horn, CTXsp: cortical subplate, DG: dentate gyrus, DORpm: poly-modal association cortex-related region, DORsm: sensory-motor cortex-related region, ECT: ectorhinal area, MOp: primary motor area, MOs: secondary motor area, PAL: pallidum, RSP: retrosplenial area, SSp: primary somatosensory area, STR: striatum, STRd: striatum dorsal region, SUB: subiculum, TEa: temporal association areas, VIS: visual areas, VISC: visceral area.
