# Supplementary material for: Phospholipid scramblase Xkr8 is required for developmental axon pruning via phosphatidylserine exposure
Source: EMBO J. 2023 May 22;42(14):e111790. doi: 10.15252/embj.2022111790 (PMC10350823; doi:10.15252/embj.2022111790)
Supplement: Supplementary file 2 — Expanded View Figures PDF [file EMBJ-42-e111790-s008.pdf]

Expanded View Figures

Figure EV1. Immunolabeling of Xkr8 in *Cx3cr1*::tdTomato microglia.

A, B Immunofluorescence labeling of total Xkr8 (*yellow*) within $tdTomato^{+}$ microglia (*red*) in the S1 cortex of P0 mouse in mosaic image (A) and enlarged representative cells in layer 4 (B, 1), layer 6 (B, 2) and corpus callosum (B, 3 and 4); *scale bars* 100 µm (A) and 10 µm (B).

▶

© 2023 The Authors

© 2023 The Authors

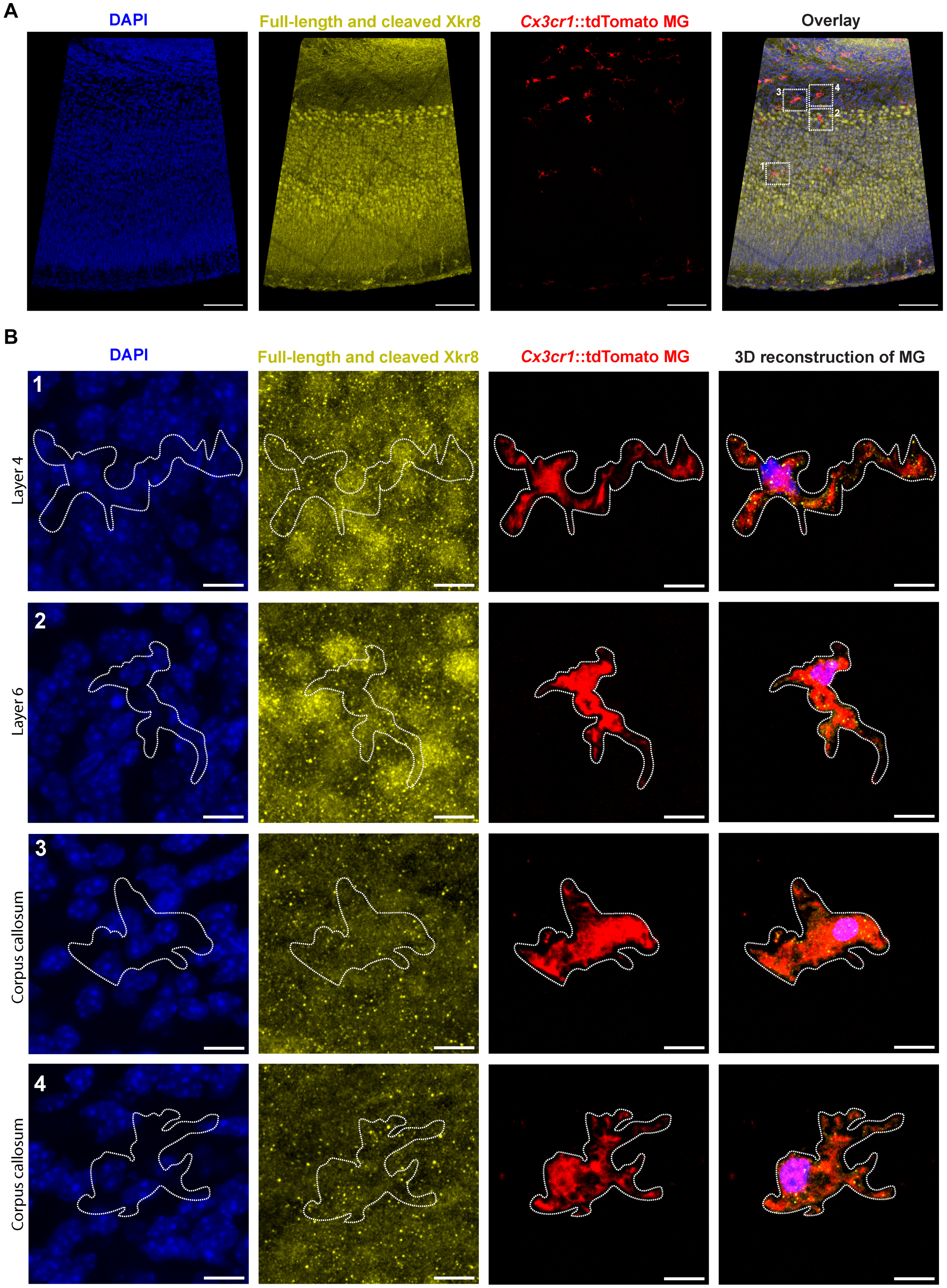

Figure EV1.

 © 2023 The Authors

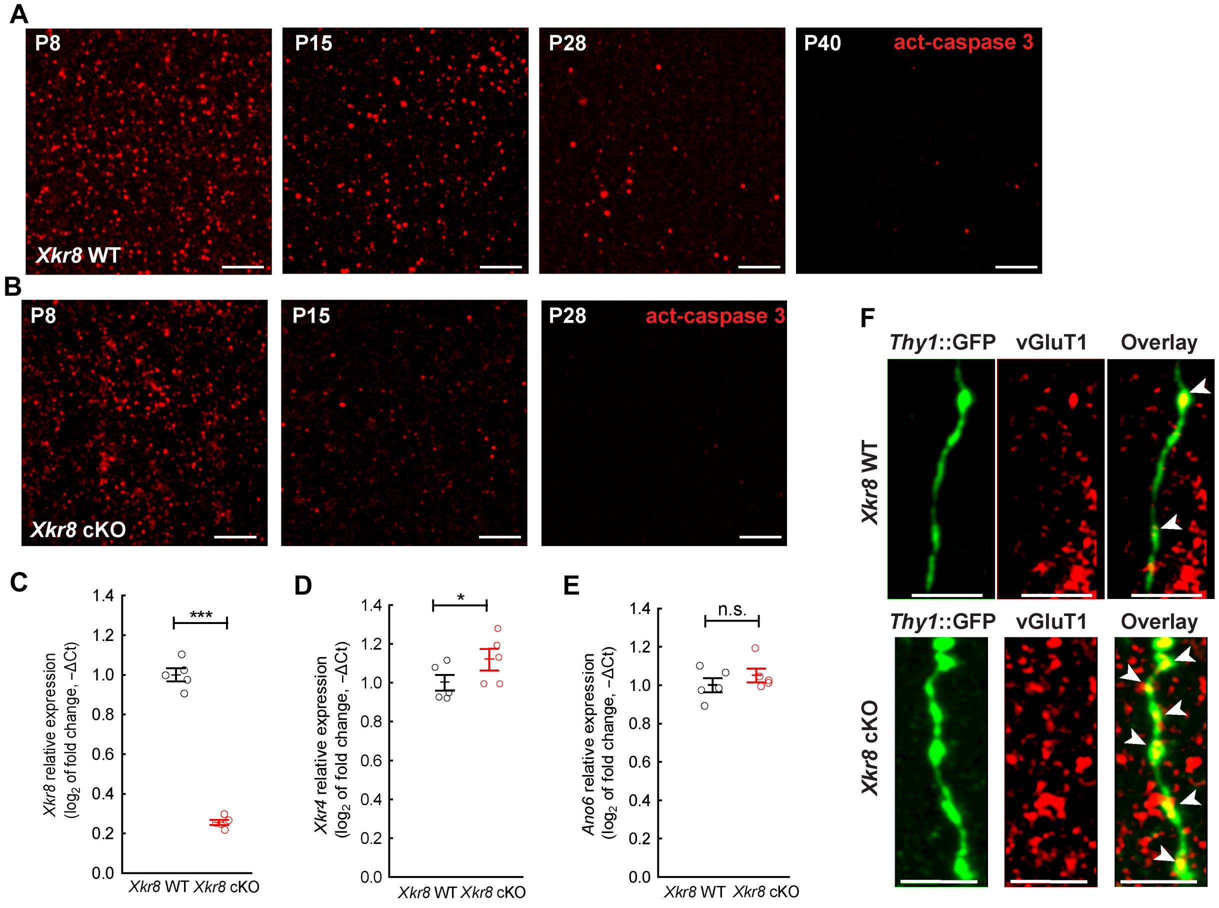

Figure EV2. The characterization of *Xkr8* cKO mouse brain.

A, B Immunofluorescence labeling of active caspase-3 in developing brain of *Xkr8* WT (A) and *Xkr8* cKO (B) mice from P8 to P40; *scale bar* 2 μm. Signal quantification is presented in Fig 2C.

C–E Expression of *Xkr8* (C), *Xkr4* (D) and *Ano6* (E) mRNA in postnatal P0 brain of *Xkr8* WT and *Xkr8* cKO mouse was measured by quantitative RT–PCR, normalized to the expression of *Gadph* at P0 and to the expression of that mRNA in *Xkr8* WT brain. Data were analyzed by one-way ANOVA, each dot represents an individual mouse, $n = 5$ per genotype group; mean ± SEM, *$P < 0.05$, ***$P < 0.001$.

F Immunofluorescence labeling of vGluT1 (*red*) in *Thy1*::GFP^{+} axons (*green*) in *Xkr8* WT and *Xkr8* cKO hippocampus. The arrows indicate axonal varicosities; *scale bar* 5 μm.

Source data are available online for this figure.

© 2023 The Authors

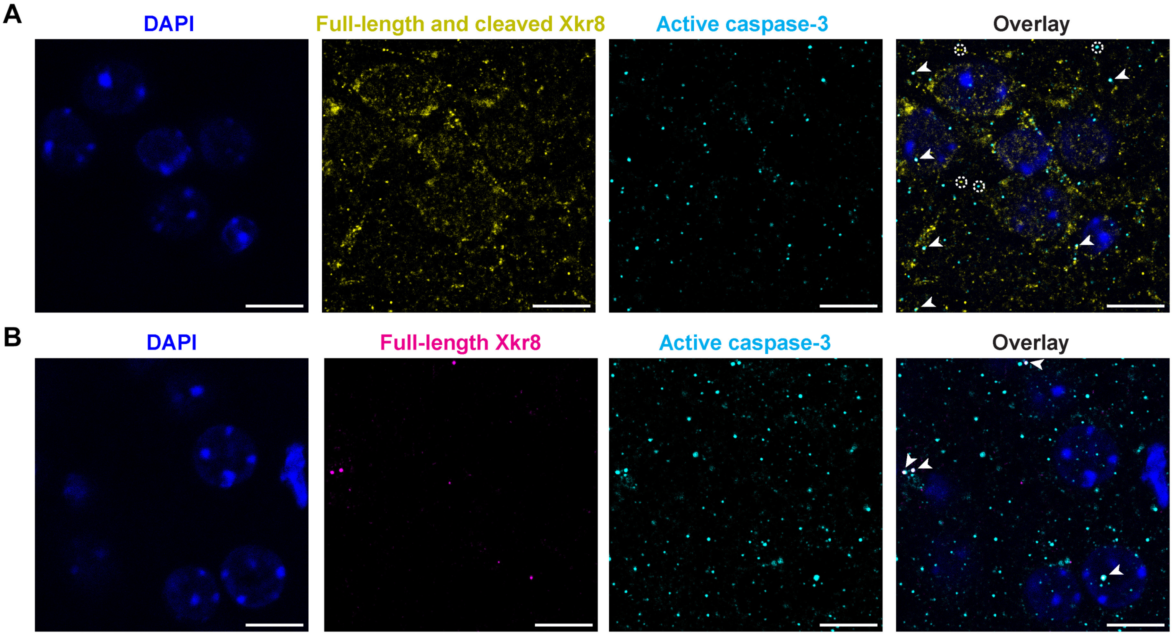

Figure EV3. The collocalization of Xkr8 and active caspase-3 in developing brain.

A, B Immunofluorescence co-labeling of active caspase-3 (*cyan*) and Xkr8 antibodies that recognize either both full-length and cleaved Xkr8 (A, *yellow*) or only full-length Xkr8 (B, *magenta*) in the S1 cortex of P8 mouse. Arrows mark co-localizing particles, dashed circles label particles that do not co-localize; *scale bar* 10 μm.

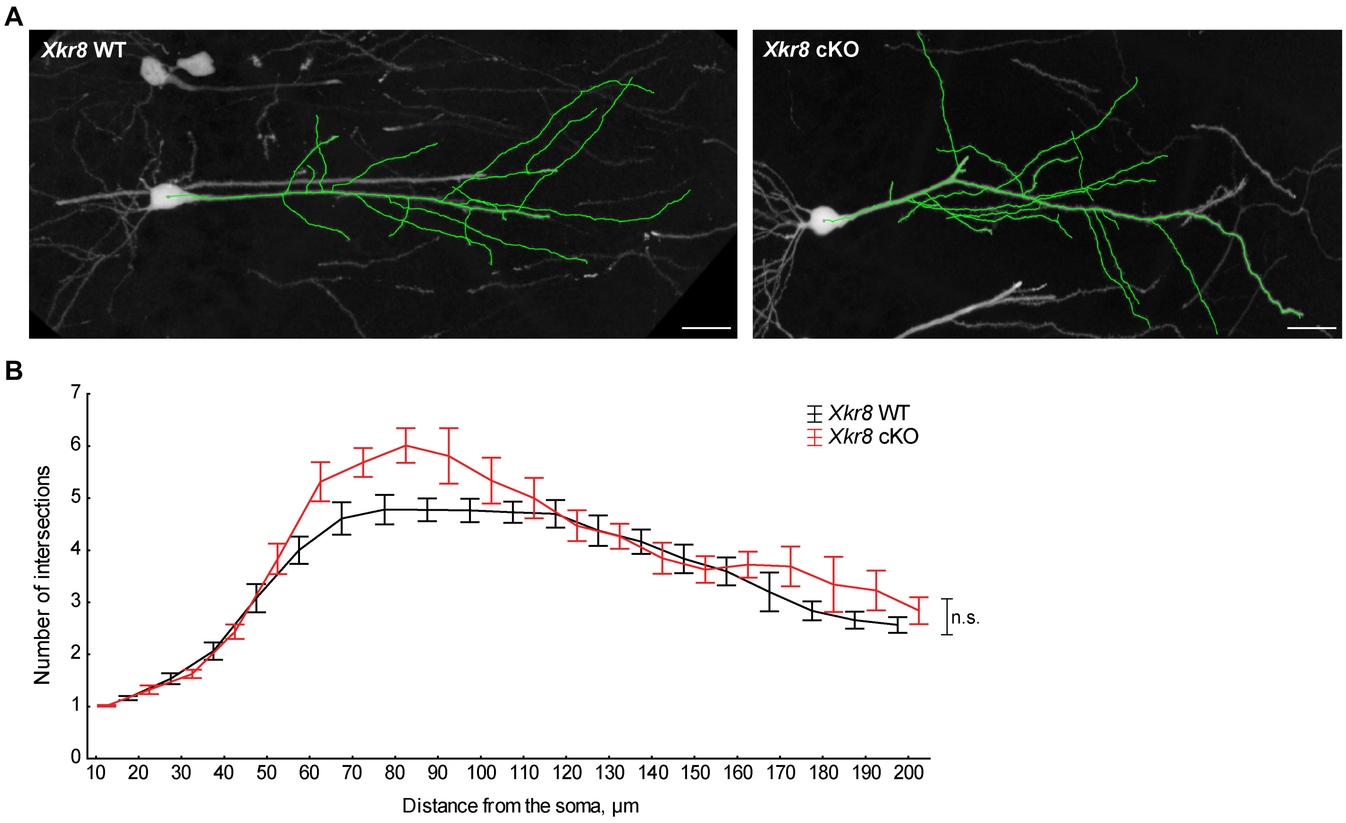

Figure EV4.

 © 2023 The Authors

Figure EV4. *Xkr8* KO did not alter dendritic morphology of pyramidal neurons.

A Representative dendritic arbors of *Thy1*::GFP+ hippocampal CA1 neurons at P28 in *Xkr8* WT and cKO brains; *scale bar* 20 μm.
B Dendritic branching patterns of *Thy1*::GFP+ CA1 neurons in *Xkr8* WT and cKO brains were defined by Sholl analysis, which quantifies the number of dendritic branches at predefined distances from the soma (Mann–Whitney test, $n = 6$ mice per genotype group; mean ± SEM).

Source data are available online for this figure.

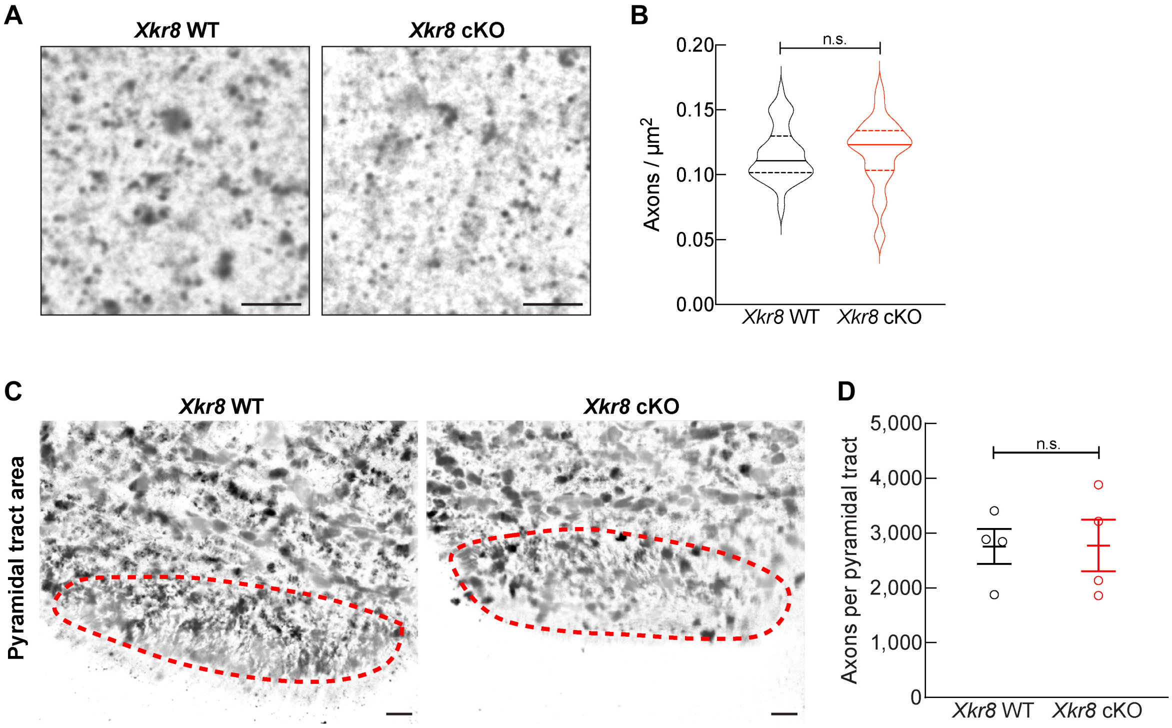

Figure EV5. The density of corticospinal axons in the medulla of P0 *Xkr8* WT and cKO mouse.

A Corticospinal axons of the pyramidal tracts in medulla of P0 *Xkr8* WT and cKO mice were visualized by Palmgren staining in high magnification (60×) to quantify axonal density; *scale bar* 4 μm.
B Corticospinal axon density (Mann–Whitney test, $n = 4$ mice per genotype, the data are presented as median and quartiles).
C Corticospinal axons of the pyramidal tracts in medulla of P0 *Xkr8* WT and cKO mice were visualized by Palmgren staining in low magnification (20×) to measure pyramidal tract area, delineated by dashed lines; *scale bar* 20 μm.
D Corticospinal axon count per whole pyramidal tract area (two-tailed Student's *t*-test, $n = 4$ mice per genotype, the data are presented as mean ± SEM).

Source data are available online for this figure.

© 2023 The Authors
