# Supplementary material for: Phospholipid scramblase Xkr8 is required for developmental axon pruning via phosphatidylserine exposure
Source: EMBO J. 2023 May 22;42(14):e111790. doi: 10.15252/embj.2022111790 (PMC10350823; doi:10.15252/embj.2022111790)
Supplement: Supplementary file 4 — PDF+ [file EMBJ-42-e111790-s010.pdf]

Article

SOURCE DATA | TRANSPARENT PROCESS | OPEN ACCESS

THE EMBO JOURNAL

Phospholipid scramblase Xkr8 is required for developmental axon pruning via phosphatidylserine exposure

Urte Neniskyte[1,2,3,*], Ugne Kuliesiute[1,2], Auguste Vadisiute[2,3,4], Kristina Jevdokimenko[2], Ludovico Coletta[5], Senthilkumar Deivasigamani[3], Daina Pamedytyte[1,2], Neringa Daugelaviciene[1,2], Daiva Dabkeviciene[1], Emerald Perlas[3], Aditya Bali[2], Bernadette Basilico[4], Alessandro Gozzi[5], Davide Ragozzino[4] & Cornelius T Gross[3,**]

Abstract

The mature mammalian brain connectome emerges during development via the extension and pruning of neuronal connections. Glial cells have been identified as key players in the phagocytic elimination of neuronal synapses and projections. Recently, phosphatidylserine has been identified as neuronal "eat-me" signal that guides elimination of unnecessary input sources, but the associated transduction systems involved in such pruning are yet to be described. Here, we identified Xk-related protein 8 (Xkr8), a phospholipid scramblase, as a key factor for the pruning of axons in the developing mammalian brain. We found that mouse Xkr8 is highly expressed immediately after birth and required for phosphatidylserine exposure in the hippocampus. Mice lacking Xkr8 showed excess excitatory nerve terminals, increased density of cortico-cortical and cortico-spinal projections, aberrant electrophysiological profiles of hippocampal neurons, and global brain hyperconnectivity. These data identify phospholipid scrambling by Xkr8 as a central process in the labeling and discrimination of developing neuronal projections for pruning in the mammalian brain.

Keywords callosal projections; circuit maturation; phosphatidylserine; phospholipid scrambling; synaptic pruning

Subject Categories Development; Neuroscience

DOI 10.15252/embj.2022111790 | Received 31 May 2022 | Revised 18 April 2023 | Accepted 25 April 2023 | Published online 22 May 2023

The EMBO Journal (2023) 42: e111790

Introduction

The development of the vertebrate central nervous system involves the outgrowth of axons to form synaptic contacts in target regions and the subsequent elimination of a subset of these connections—a phenomenon called developmental pruning—to produce the functional circuitry of the mature brain (Innocenti *et al*, 1977; Innocenti, 1981; O'Leary, 1987; LaMantia & Rakic, 1990; Portera-Cailliau *et al*, 2005). Pruning has a dramatic impact on brain connectivity. In primates, for example, 70% of cortical axons are lost shortly after birth (LaMantia & Rakic, 1990). In the few systems where pruning has been studied in some detail, such as the neuromuscular junction, the pruning of connections is accompanied by the functional maturation of the remaining synapses (Personius & Balice-Gordon, 2001), suggesting that there is competition between incoming axons for limited synaptic resources and that an initial exuberance of inputs may provide robustness to neurodevelopmental axon pathfinding (Innocenti & Price, 2005). However, the role of pruning in the brain as a whole has remained unclear because a lack of understanding about the molecular signals involved has until now prevented its selective perturbation.

Recent findings have shown that glial cells contribute to synapse and axon elimination and remodeling (Paolicelli *et al*, 2011; Schafer *et al*, 2012; Chung *et al*, 2013; Kurematsu *et al*, 2022) offering a possible avenue for the identification of pruning signals. Interactions between glial cells and neurons involve three components: glial receptors, soluble opsonins, and neuronal surface signals. Microglial complement receptor 3 (Schafer *et al*, 2012) and astrocytic Mer tyrosine kinase (Chung *et al*, 2013) promote phagocytosis, while microglial SIRPα (Lehrman *et al*, 2018) inhibits phagocytic uptake of synaptic material. Soluble opsonins, such as complement components C1q, C3, and C4 (Schafer *et al*, 2012; Sekar *et al*, 2016), bind to glial targets and facilitate their recognition, and neuronal *eat-me* and *spare-me* signals either promote or inhibit, respectively, phagocytic glia–neuron interactions. Recently, CD47 has been shown to limit microglial elimination of axonal inputs (Lehrman *et al*, 2018), and surface presentation of phosphatidylserine (PtdSer) has been demonstrated to function as an *eat-me* signal for the elimination of neuronal processes during invertebrate metamorphosis (Sapar *et al*, 2018) and during adult neurogenesis in olfactory bulb and hippocampus (Kurematsu *et al*, 2022). PtdSer is known to interact with phagocytosis proteins involved in synaptic pruning in the mammalian brain [such as C1q (Paidassi *et al*, 2008) or MerTK (Chung *et al*, 2013)] and has been implicated in synaptic pruning via microglial adhesion G protein-coupled receptor ADGRG1/GPR56 (Li *et al*, 2020; Scott-Hewitt *et al*, 2020). However, so far, the

© 2023 The Authors. Published under the terms of the CC BY 4.0 license.

mechanism regulating the exposure of PtdSer during developmental circuit refinement remains unknown.

The exposure of PtdSer is controlled by the balance of its scrambling and internalization (Lemke, 2019). PtdSer is ubiquitously present on the intracellular leaflet of the plasma membrane of eukaryotes under homeostatic conditions. ATP-dependent flippases maintain PtdSer on the intracellular leaflet and phospholipid scramblases promote its exposure on the extracellular surface (Nagata *et al*, 2016). PtdSer can be externalized by two types of scramblases: Xk-related proteins (Suzuki *et al*, 2014) and TMEM16 proteins (Suzuki *et al*, 2013b). Xk-related protein 8 (Xkr8) is one of the first identified phospholipid scramblases and is activated by caspase-3 cleavage (Suzuki *et al*, 2013a). This feature suggests that Xkr8 could respond to caspase-dependent apoptosis-like molecular processes to drive PtdSer exposure and synaptic pruning (Erturk *et al*, 2014; Gyorffy *et al*, 2018). Here we investigated a possible role for PtdSer scrambling by Xkr8 as a neuronal *eat-me* signal for the elimination of connections during brain development.

Results

Xkr8 scramblase is dynamically regulated in developing brain

Xkr8 was previously identified as a caspase-activated phospholipid scramblase promoting PtdSer exposure on non-neuronal cells (Suzuki *et al*, 2013a). To investigate a possible involvement of Xkr8 in PtdSer scrambling in the developing brain, we first examined the expression of *Xkr8* mRNA using real-time PCR in hippocampus samples from birth (P0) to adulthood (P90). Data were normalized to average adult P90 expression levels to derive a $-\Delta\Delta$Ct value. We found that Xkr8 was highly expressed shortly after birth with more than 200-fold higher mRNA levels at P0 compared to P90 (two-way ANOVA $F_{(5,21)} = 6.430$, $P < 0.001$; Fig 1A). Xkr8 expression fell significantly during the first week of life to approximately 3-fold above P90 levels and then gradually reduced to adult levels. *Xkr8* mRNA expression in both hippocampus and somatosensory cortex in adult brain was confirmed by RNA *in situ* hybridization (ISH) (Appendix Fig S1C). On the other hand, expression of Xkr4, another lipid scramblase from the Xk-related family, remained stable in the brain through development ($-1.5 < -\Delta\Delta\text{Ct} < 1.5$ at all ages, Appendix Fig S1A) and Xkr9, another Xk-related scramblase, was not detectably expressed, in line with previously published data (Suzuki *et al*, 2014). Therefore, Xkr8 appears to be the phospholipid scramblase, which is selectively regulated during early brain development (Appendix Fig S1B).

Full-length Xkr8 is inactive and it is activated by caspase-3- or caspase-7-mediated proteolytic cleavage of its 5 kDa C-terminal domain (Suzuki *et al*, 2013a). To assess whether caspase-mediated Xkr8 cleavage occurs in the developing brain, we compared immunofluorescence labeling of developing and adult brain using two antibodies, one of which recognizes full-length, but not cleaved Xkr8, while the other recognizes both full-length and cleaved protein (Fig 1B). Consistent with its mRNA expression profile we found that total Xkr8 levels were higher in P0 and P8 brains compared to adult (Fig 1C and E). Interestingly, full-length, uncleaved Xkr8 was not detected in the early postnatal brain (Fig 1D and F). These data demonstrate that expression of Xkr8 in hippocampus is high in the first postnatal week, but declines thereafter, and suggest that it undergoes proteolytic cleavage preferentially during the early postnatal period.

In situ hybridization staining and immunofluorescent labeling localized Xkr8 to mostly pyramidal neurons. Importantly, we found only weak immunolabeling of Xkr8 within tdTomato$^+$ microglia (Fig EV1A and B), suggesting that Xkr8 is specifically expressed in neurons of developing brain.

Because caspase-3 has previously been suggested to be required for Xkr8 activation (Suzuki *et al*, 2013a) and to be involved in neuronal pruning (Erturk *et al*, 2014), we assessed how caspase-3 activation changes in the developing hippocampus. Immunolabeling for active caspase-3 showed a punctate pattern with the highest density observed at P8 (Figs 1G and EV2A). Furthermore, at P8 active caspase-3 co-localized with Xkr8, in particular with its uncleaved isoform (Fig EV3A and B). These data argue for a link between caspase-3 activation and Xkr8 cleavage in neurons in the perinatal hippocampus that may mediate PtdSer exposure, which guides developmental synaptic pruning.

To assess a potential functional involvement of Xkr8 in synaptic pruning during perinatal development, we developed mice selectively lacking Xkr8 in excitatory cortical and hippocampal neurons (*Xkr8*^{flx}/*Xkr8*^{flx};*Emx1*::Cre;*Thy1*::EGFP, called *Xkr8* cKO; Fig 1C–F). *Xkr8* cKO mice did not have any gross developmental or behavioral abnormalities. Importantly, the expression of other well-described scramblases Xkr4 and TMEM16F (encoded by *Ano6*) was only slightly increased in *Xkr8* cKO mice, indicating that the loss of Xkr8 could not have been counteracted by compensatory mechanisms upregulating other scramblases (Fig EV2C–E).

Using *Xkr8* cKO mice, we first assessed whether Xkr8 is required for the exposure of PtdSer in the brain. Selective visualization of extracellular PtdSer in the tissue is challenging, because tissue fixation permeabilizes cell membranes and allows fluorescently labeled PtdSer-binding proteins to access ubiquitous intracellular PtdSer. To overcome this issue, we performed PtdSer labeling with the fluorescently tagged high affinity PtdSer-binding protein Annexin V in live *ex vivo* organotypic hippocampal slices (Vermes *et al*, 1995; Weinhard *et al*, 2018a). Organotypic *Xkr8* cKO cultures showed a significant reduction of PtdSer exposure compared to WT controls (two-tailed *t*-test $P < 0.001$; Fig 2A and B), suggesting that Xkr8 is a pivotal lipid scramblase in brain tissue at this time period. In contrast, activated caspase-3 immunolabeling was not significantly affected in *Xkr8* cKO mice (two-way ANOVA $F_{(2,30)} = 0.86$, $P = 0.433$; Figs 2C and EV2B). Therefore, our *Xkr8* cKO experiments infer that lipid scrambling by Xkr8 is required for PtdSer exposure in brain tissue.

Xkr8 is required for efficient axonal pruning

Next, we investigated the impact of defective PtdSer exposure on synaptic development by evaluating the morphology and density of excitatory pre- and postsynaptic structures in *Xkr8* WT and cKO mice across postnatal development. To be able to analyze specific neuronal structures, such as boutons, spines, dendrites, or axons, we used *Thy1*::GFP transgenic mice in which 2–3% of pyramidal neurons express cytoplasmic green fluorescent protein (GFP) (Feng

 © 2023 The Authors

et al, 2000). To investigate PtdSer exposure in living brain tissue, we first performed PtdSer labeling with the fluorescently tagged high affinity PtdSer-binding protein Annexin V in *ex vivo* organotypic hippocampal slices (Vermes *et al*, 1995). PtdSer exposure was observed in a punctate pattern that overlapped with neuronal labeling of excitatory pyramidal neurons (*Thy1*::EGFP; Appendix Fig S2A–D). We then reconstructed 3D surfaces of GFP$^+$ neurons from confocal *z* stacks and examined Annexin V labeling within

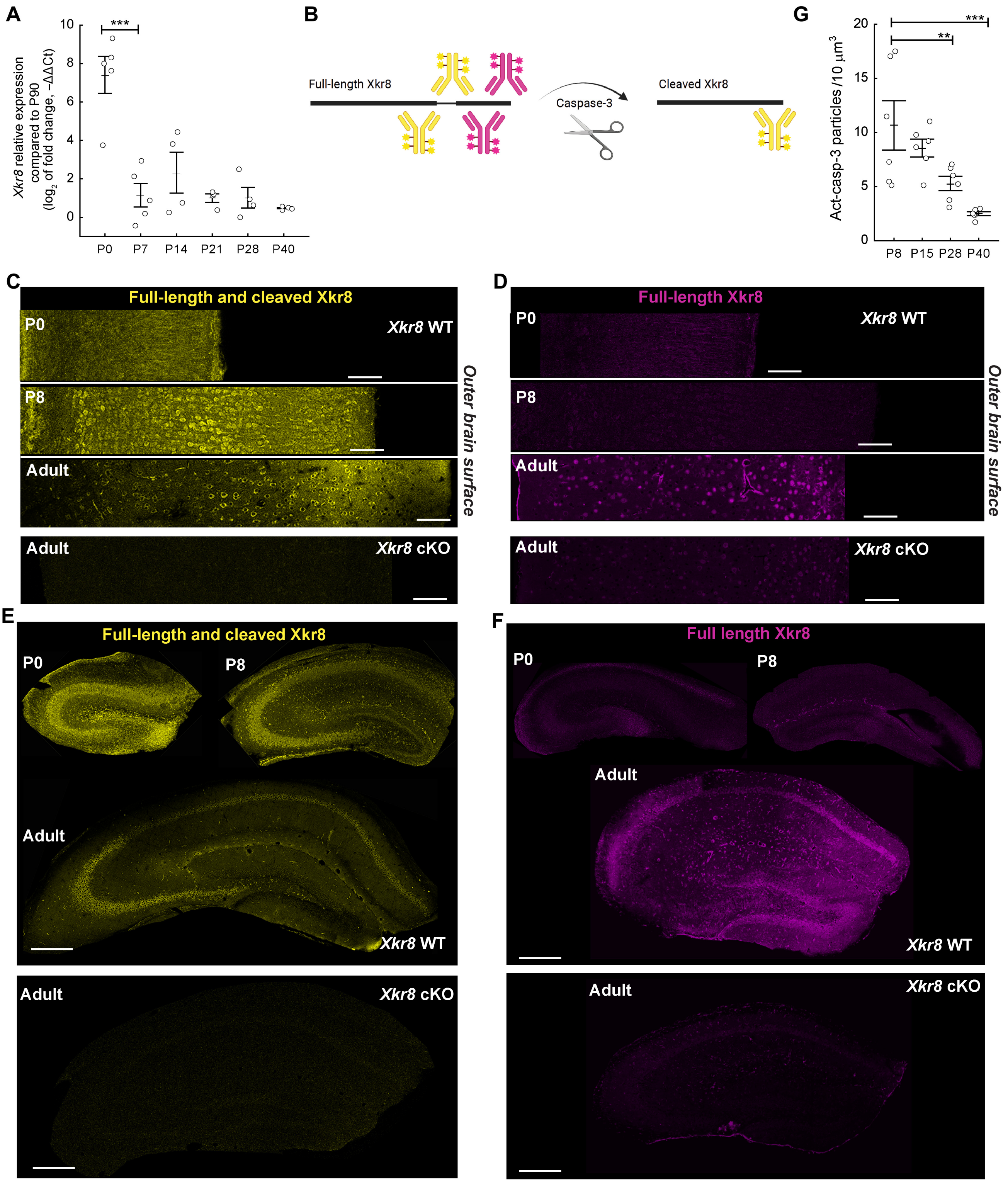

Figure 1.

Figure 1. The expression of Xkr8 scramblase in the developing mouse brain.

A *Xkr8* mRNA expression immediately after birth and during postnatal development. Quantitative RT–PCR data were normalized to *Xkr8* mRNA expression at P90 (one-way ANOVA, each dot represents an individual mouse, $n = 4$–6 per age group).

B To visualize the cleavage of Xkr8, two distinct antibodies were used that recognize either both full-length and cleaved Xkr8 (*yellow*) or full-length Xkr8 only (*magenta*).

C–F (C, E) Immunofluorescence labeling with an antibody that recognizes both full-length and cleaved Xkr8 in developing (P0, P8) and adult S1 cortex and hippocampus. (D, F) Immunofluorescence labeling with an antibody recognizing full-length uncleaved Xkr8 in developing (P0, P8) and adult S1 cortex and hippocampus. Loss of Xkr8 immunosignal in double transgenic *Xkr8*$^{flx/flx}$;*Emx1*::Cre animals (*Xkr8* cKO) is observed with both Xkr8 antibodies (C–F).

G Immunofluorescence labeling of active caspase-3 in developing brain from P8 to P40 in *Xkr8* WT brains was analyzed by nested design and mixed model ANOVA, each dot represents an individual mouse ($n = 6$ per age group; images are presented in Fig EV2A).

Data information: Data presented as mean ± SEM; $^{**}P < 0.01$, $^{***}P < 0.001$; *scale bars* 100 μm (C, D) and 300 μm (E, F).
Source data are available online for this figure.

these surfaces. We observed that PtdSer exposure significantly colocalized with boutons compared to axonal shafts (Appendix Fig S2B) suggesting that PtdSer is preferentially exposed on synaptic structures in developing neurons. To a smaller extent, we also observed preferential PtdSer exposure on dendritic spines compared to dendritic shafts (Appendix Fig S2D).

To investigate synaptic pruning in *Xkr8* cKO hippocampus, we then assessed the dynamics of axonal bouton size and density. Axonal boutons were identified by their typical morphology, as these varicosities were confirmed to be vGluT1^{+} in both *Xkr8* WT and cKO brains (Fig EV2F). A gradual decrease of axonal bouton size between P8 and P28 was observed in *Xkr8* WT mice (Fig 3A and B), but not in *Xkr8* cKO mice, with bouton size becoming significantly greater in cKO mice when compared to WT littermates at P28 (two-way ANOVA $F_{(2,30)} = 3.54$, $P = 0.04$; Fig 3A and B). No differences in the density of boutons along individual axons were observed in *Xkr8* WT and cKO animals (Fig 3A and C). Moreover, no differences in the density of excitatory dendritic spines were observed across the early postnatal period in *Xkr8* cKO brains when compared to wild-type controls (Fig 3A and E), nor was any change in total length of excitatory neuron dendritic branches or branching pattern detected by Sholl analysis (Figs 3F and EV4A and B).

In contrast, the absolute density of cortical boutons, as assessed by vGluT1-immunopositive (vGluT1^{+}) puncta, showed a significant, 3-fold increase in *Xkr8* cKO mice compared to wild-type controls at P8 (Fig 3G and J). To assess whether Xkr8 was indeed required for the removal of axons in developing brain, we evaluated the phagocytosis of neurofilaments (Fig 4A and B, SMI321) and axonal boutons (Fig 4C and D, vGluT1) by Iba1^{+} microglia cells. We found significantly decreased uptake of both pan-axonal and bouton material by *Xkr8* cKO microglia (Fig 4B and D). In contrast, the internalization of dendritic spines (Fig 4E and F, PSD95) was not affected. Importantly, the increase of vGluT1^{+} puncta was observed at P8, before the formation of the majority of cortical excitatory synapses, suggesting that it involves the elimination of axonal material, rather than synapses, thus explaining why PSD95 uptake was not affected (Figs 3E and 4F). Interestingly, at least some of the difference in bouton density was restored at later developmental stages (Fig 3G and J), possibly as a result of the elaboration of surviving axons in wild-type animals.

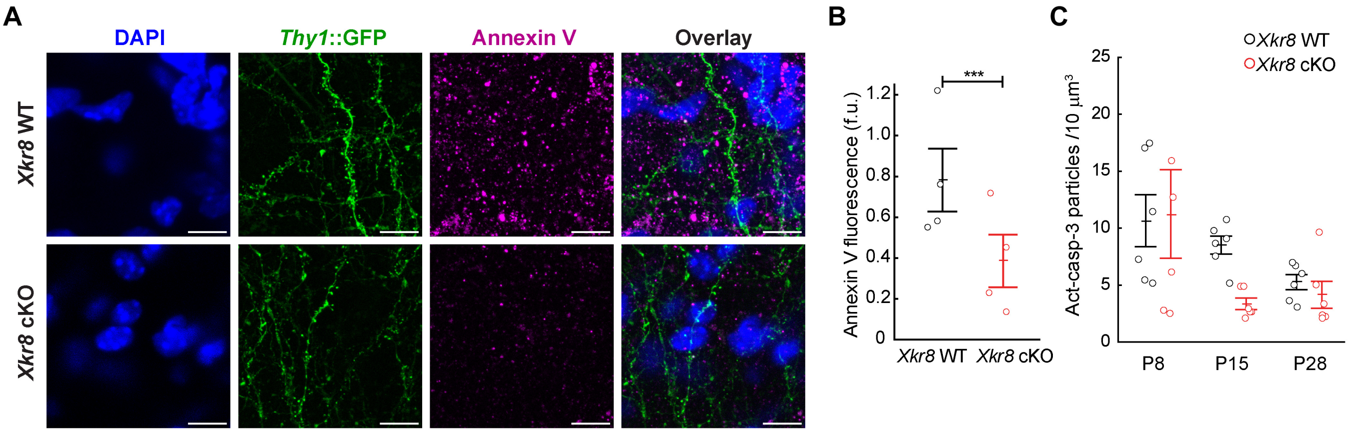

Figure 2. Reduced PtdSer exposure in mouse hippocampus lacking Xkr8 scramblase.

A, B Organotypic slices of *Xkr8* WT and cKO hippocampus with *Thy1*::GFP neurons (*green*) were labeled with PtdSer-binding fluorescently tagged Annexin V (*magenta*) at 16–19 DIV. The fluorescence of bound Annexin V was quantified on ImageJ by measuring the integrated density of fluorescent structures within the same volume for each slice and expressing the value as fluorescence units (f.u.; two-tailed Student's *t*-test, each dot represents an individual slice preparation, $n = 4$).

C Immunofluorescence labeling of active caspase-3 in developing brain from P8 to P28 in *Xkr8* WT and *Xkr8* cKO brains was quantified and compared by nested design and mixed model ANOVA ($F_{(2,30)} = 0.86$, $P = 0.433$), each dot represents an individual mouse ($n = 6$ per age group) (images are presented in Fig EV2A and B).

Data information: Data presented as mean ± SEM; $^{***}P < 0.001$; *scale bar* 10 μm.
Source data are available online for this figure.

 © 2023 The Authors

To test whether Xkr8 functions cell autonomously to regulate axonal pruning, we compared the density of cortical versus thalamic excitatory boutons that lack or express Xkr8, respectively, in our cortical cell-specific cKO strain (Fig 3I). The density of thalamo-cortical excitatory boutons that do not lack Xkr8 in our cKO mice, as assessed by vGluT2$^+$ puncta, was only modestly increased at P8 and normalized at P28 in *Xkr8* cKO mice when compared to wild-type controls (Fig 3H and K). Interestingly, adult *Xkr8* cKO mice continued to show an excess of cortical relative to thalamic inputs (Fig 3L). These findings confirm a predominantly cell autonomous effect of Xkr8 on axonal remodeling during the first postnatal week, but also demonstrate a small,

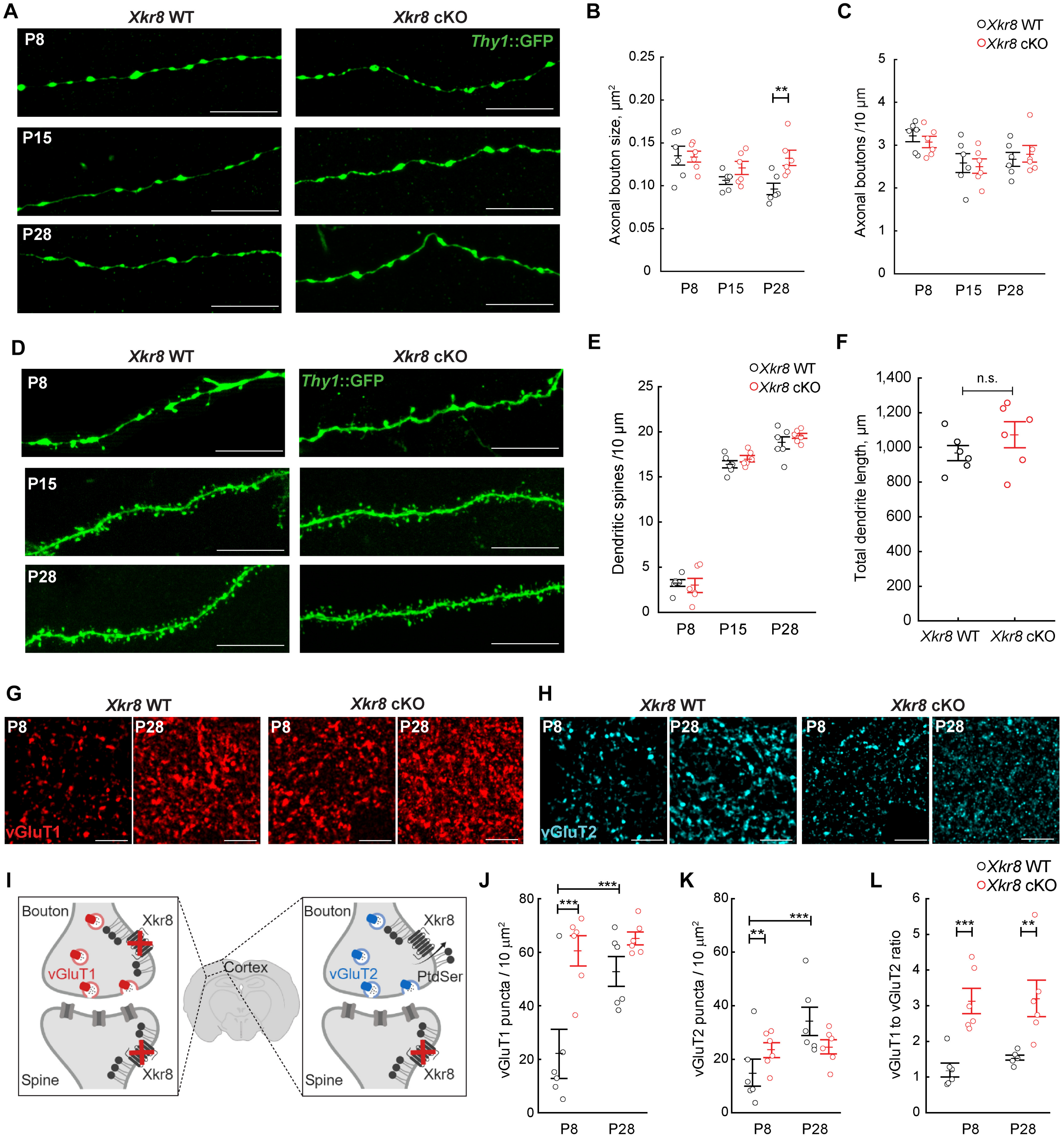

Figure 3.

◀ **Figure 3. Increased density of presynaptic puncta in *Xkr8* knockout.**

A–C The size and density of axonal boutons on *Thy1*::GFP axons (*green*) in *Xkr8* WT and cKO animals (nested design and mixed model ANOVA, each dot represents an individual mouse, $n = 6$ per age and genotype group).

D, E Dendritic spine morphology (D) and density (E) of *Thy1*::GFP$^+$ CA1 neurons in *Xkr8* WT and cKO animals from P8 to P28 (nested design and mixed model ANOVA, each dot represents an individual mouse, $n = 5$–6 per age and genotype group).

F Total length of dendritic branches of *Thy1*::GFP$^+$ CA1 neurons in *Xkr8* WT and cKO brains (nested design and mixed model ANOVA, each dot represents an individual mouse, $n = 6$ per genotype group).

G, H Immunofluorescence labeling of vGluT1$^+$ and vGluT2$^+$ boutons in L4 somatosensory cortex of *Xkr8* WT and cKO brains.

I Schematic representation of selective Xkr8 loss in cortical vGluT1$^+$ pre- and post-synaptic neurons, but not thalamocortical vGluT2$^+$ projections in L4 of somatosensory cortex of *Xkr8* cKO brains.

J–L The density of cortical (vGluT1$^+$, J) and thalamic (vGluT2$^+$, K) and the ratio of vGluT1$^+$ and vGluT2$^+$ inputs (L) in *Xkr8* WT and cKO brains (nested design and mixed model ANOVA, each dot represents an individual mouse, $n = 6$ per age and genotype group).

Data information: Data presented as mean ± SEM; $^{**}P < 0.01$, $^{***}P < 0.001$; *scale bars* 10 μm (A, D) and 2 μm (G, H).
Source data are available online for this figure.

but significant cell non-autonomous impact of Xkr8, possibly due to a contribution of postsynaptic Xkr8 to axonal maturation (Fig 3I) or the cleavage and release of surface exposed PtdSer as lysophosphatidylserine that has been shown to activate microglia and promote phagocytosis (Frasch & Bratton, 2012; Blankman *et al*, 2013).

As there are indications that brain maturation may be sex-specific (Weinhard *et al*, 2018b), we have used both male and female mice in this study. The statistical analysis of data presented in Figs 1–3 using sex as an independent variable, revealed that axonal bouton size and density as well as the density of vGluT1$^+$ puncta and, accordingly, vGluT1$^+$ to vGluT2$^+$ ratio may be sex-dependent (Appendix Table S1). However, due to low number of animals in each sex group, these results only indicate tendencies rather than being conclusive and warrant further investigation.

The first postnatal week is a period of intense cortical axon pruning but is also marked by cortical pyramidal cell apoptosis, a process known to involve PtdSer exposure (Wong & Marin, 2019). To examine whether the increase in the density of cortical excitatory puncta seen in *Xkr8* cKO mice at P8 might be driven by altered neuronal cell death rather than axonal pruning, we quantified cortical cell density and thickness at P8 and P28. Neither cortical thickness nor cell density was altered in *Xkr8* cKO brains at P8 when compared to wild-type controls (Appendix Fig S3A–E), pointing to a selective role for Xkr8 in axonal pruning rather than cell elimination. We did observe, however, a significant reduction in cortical thickness in *Xkr8* cKO mice at P28, although this was not reflected in any change in cell density (Appendix Fig S3A–E). We cannot explain the reduction in cortical thickness at this point, but these data do not support a role for Xkr8 in neuronal apoptosis in the first postnatal week.

In the absence of a change in bouton density along individual axons (Fig 3C), the increase in the absolute density of vGluT1$^+$ puncta (Fig 3J) may point to a deficiency in the elimination of entire axon arbors, rather than the removal of individual boutons, a process known to occur selectively during the first postnatal week of cortical development (Portera-Cailliau *et al*, 2005). To directly test whether Xkr8 is required to promote the removal of axons during brain development, we quantified the number of axons in corticospinal tract in the coronal sections of the medulla of *Xkr8* cKO and wild-type control animals using modified Palmgren staining (Goshgarian, 1977). Significantly more axons per bundle were found in *Xkr8* cKO at P8 compared to wild-type control brains at P8, but was normalized by P28 (Fig 5A and B), in line with the trajectory observed in the density of cortical vGlut1$^+$ puncta. Importantly, we found no differences in the density of corticospinal axons or total axonal number per corticospinal tract at P0 (Fig EV5A–D), suggesting that lack of Xkr8 did not affect prenatal axonal outgrowth and further supporting its role in postnatal axonal pruning.

To further investigate Xkr8 in the postnatal development of axonal tracts, we examined the pruning of inter-hemispheric cortical axons in the primary (S1) and secondary (S2) somatosensory cortex that are known to undergo activity-dependent pruning during the early postnatal period (De León Reyes *et al*, 2019). The fluorescently labeled retrograde tracer cholera toxin B (CTB) was injected directly into the corpus callosum at P30-32 to label callosal projection neurons at the completion of developmental pruning (Fig 5C). As reported previously, we observed sparse labeling in layer L4 of S1 and denser labeling in layer L4 of S2 of wild-type control mice (Fig 5D). *Xkr8* cKO mice, on the other hand, showed a significant, 2-fold increase in back-labeled neurons in S1 compared to control animals (Fig 5D and E), suggesting a reduced efficiency of axonal pruning in the absence of Xkr8. There was also a small increase in back-labeled S2 neurons in *Xkr8* cKO mice compared to wild-type controls, consistent with the more moderate pruning seen in this area (De León Reyes *et al*, 2019) (Fig 5D and F). Even though the increase of labeled S2 neurons in *Xkr8* cKO mice was non-significant, a higher number of observations would be required to unequivocally confirm or exclude the role of Xkr8 in the pruning of S2 projections.

Altogether, these findings further support a role for Xkr8-dependent phospholipid scrambling in the elimination of cortical projections during early postnatal development.

Altered synaptic transmission in Xkr8 cKO

Axonal pruning has been proposed to be important for the topographical refinement of neuronal projections and for the maturation and strengthening of synapses in the mammalian brain (Innocenti, 1981; O'Leary, 1987; LaMantia & Rakic, 1990; Portera-Cailliau *et al*, 2005). However, until now, it has remained difficult to test these hypotheses because it was not possible to selectively disrupt axonal pruning. To determine whether postnatal Xkr8-dependent axonal pruning might have long-term consequences on circuit maturation, we examined spontaneous and evoked excitatory synaptic

 © 2023 The Authors

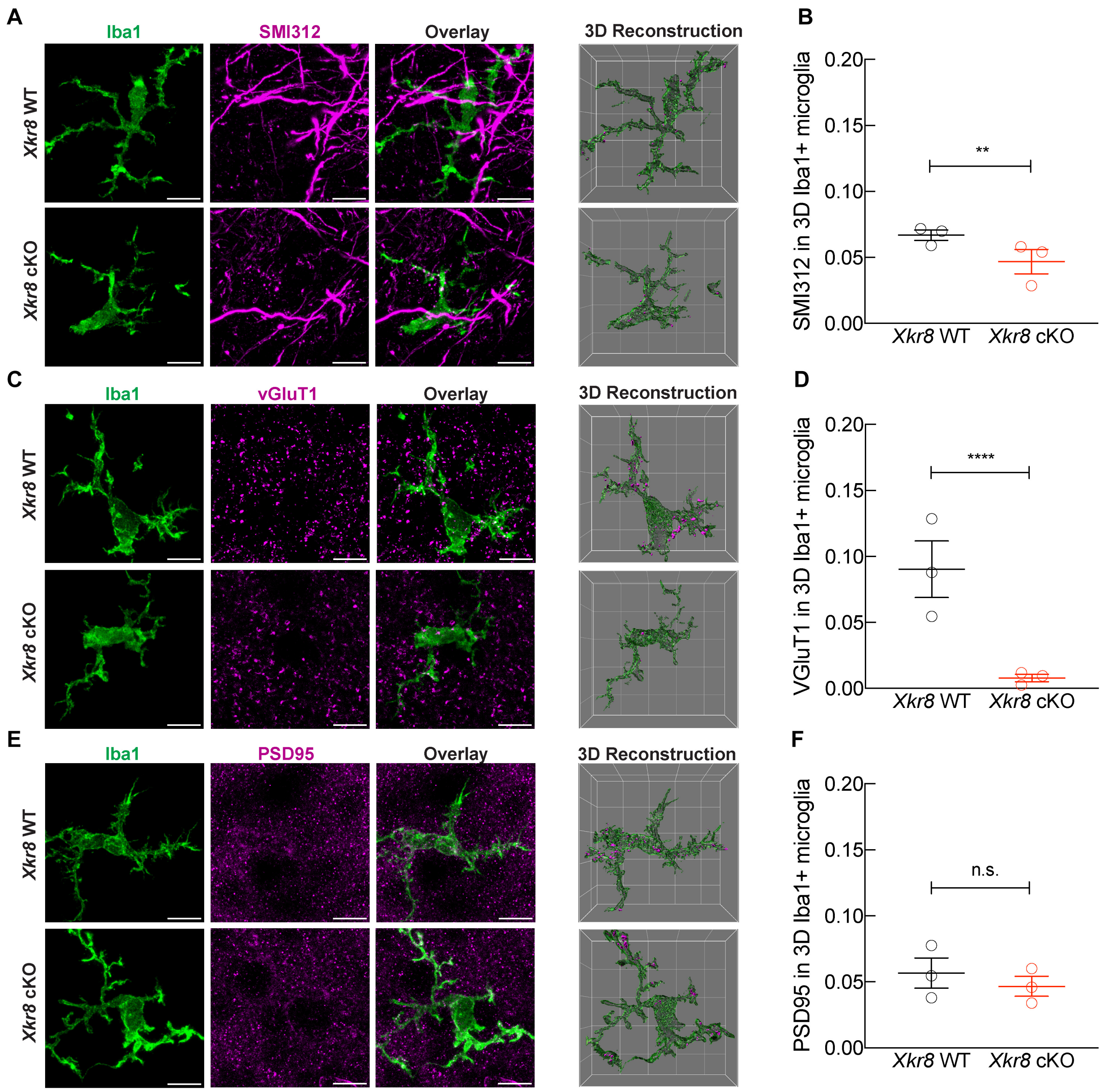

Figure 4. Xkr8 deficiency limits microglial uptake of presynaptic material in developing S1 cortex.

A–F The immunolabeling of internalized pan-axonal material (A, B), pre-synaptic vGluT1 particles (C, D) and post-synaptic PSD95 particles (E, F) within Iba1$^+$ microglia in P8 cortex of *Xkr8* WT and *Xkr8* cKO mice was quantified per individual 3D reconstructed microglial cell. The total volume of internalized particles was normalized to microglial cell volume and compared by nested design and mixed model ANOVA, each dot represents an individual mouse (n = 3 per genotype group). Data presented as mean ± SEM; $^{**}P < 0.01$, $^{****}P < 0.0001$; *scale bar* 10 µm, *grid cell* 10 µm.

Source data are available online for this figure.

activity of CA1 pyramidal neurons in hippocampal slices from *Xkr8* cKO and wild-type control mice at P40 by whole cell patch clamp electrophysiology. First, we evaluated the amplitude and frequency of spontaneous excitatory postsynaptic currents (sEPSCs) that reflect basal, action potential-dependent synaptic network activity. Pyramidal neurons from *Xkr8* cKO mice showed a significant increase in the amplitude, but not frequency of sEPSCs when compared to wild-type controls (Fig 6A–E; Appendix Fig S4A–D). On the other hand, the amplitude of evoked CA1 Schaffer collateral excitatory postsynaptic currents (eEPSCs) was not significantly altered in *Xkr8* cKO mice when compared to wild-type controls (Fig 6F and G). These findings suggest that while the strength of the majority of

© 2023 The Authors

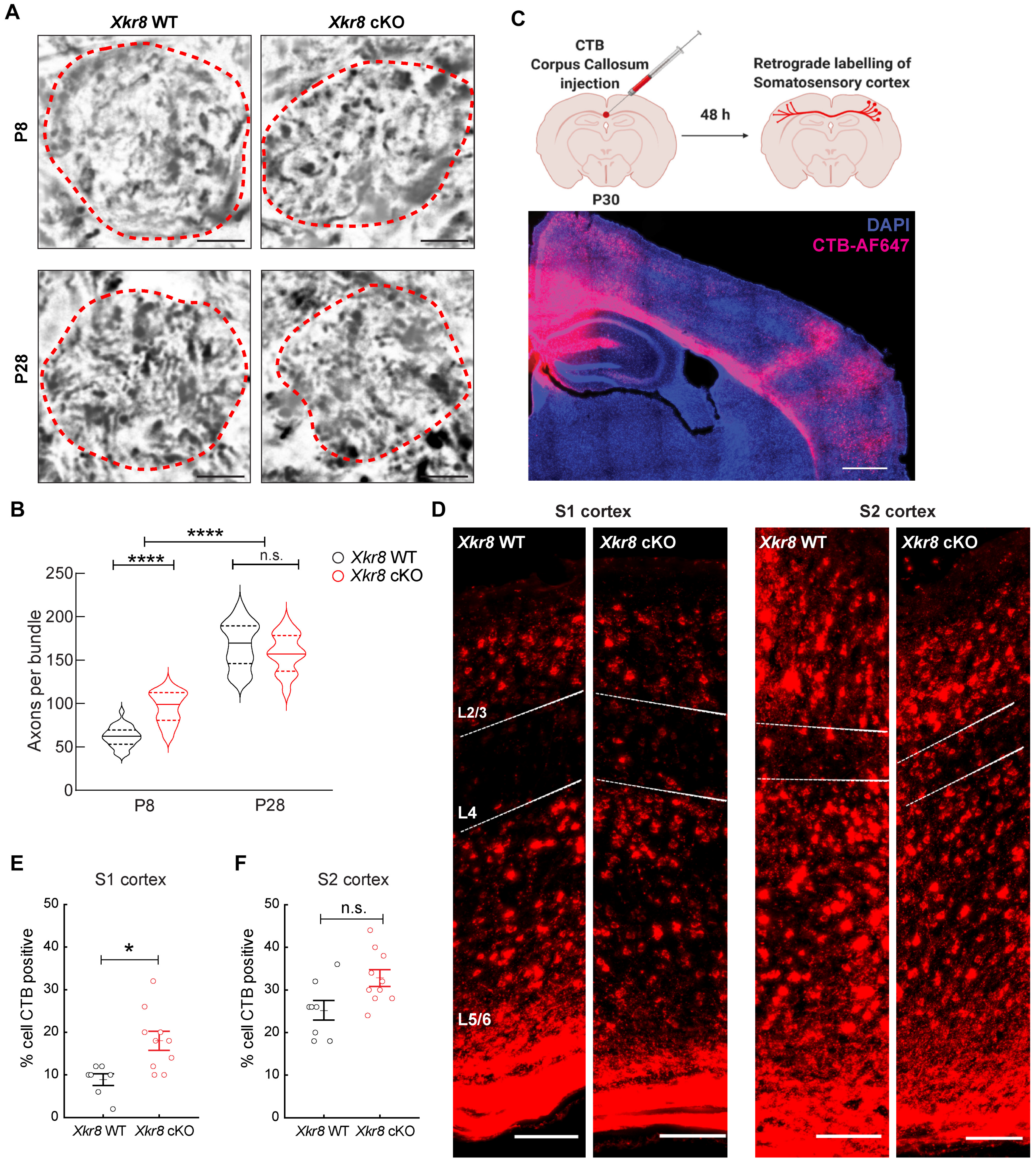

Figure 5. Excess axonal projections in *Xkr8* knockout.

A, B Corticospinal axons of the axonal bundles of pyramidal tracts in medulla of P8 and P28 *Xkr8* WT and cKO mice were visualized by Palmgren staining and quantified per each bundle, delineated by a dashed line (Mann–Whitney test, $n = 6$ mice per genotype, the data are presented as median and quartiles).

C, D To label callosal projections *in vivo*, cholera toxin subunit B (CTB) was injected stereotactically into corpus callosum of *Xkr8* WT and cKO brains at P30-32. Back-labeled neurons were quantified in L4 of primary (S1) and secondary (S2) somatosensory cortex.

E, F *Xkr8* cKO animals showed significantly more back-labeled neurons in S1-L4, but not S2-L4 indicating aberrant pruning of collosal S1 projections (nested design and mixed model ANOVA, each dot represents an individual mouse, $n = 7$–10 per genotype group).

Data information: Data presented as mean ± SEM; $^{*}P < 0.05$, $^{****}P < 0.0001$; *scale bars* 4 µm (A), 500 µm (C) and 100 µm (D).
Source data are available online for this figure.

© 2023 The Authors

excitatory synapses was unaltered, thus maintaining normal eEPSCs, which results from the activation of multiple synapses in a field, a subset of spontaneously active synapses was increased, driving the increase of mean sEPSC amplitude, which characterizes individual synapses. An analysis of the cumulative histogram of sEPSC amplitudes revealed that the significant increase in sEPSC amplitude in *Xkr8* cKO mice was driven primarily by an increase in large synaptic events (Appendix Fig S4A–D), further supporting the existence of a subset of strengthened excitatory synapses in the cKO.

On the other hand, an analysis of the AMPA and NMDA receptor components of eEPSCs revealed a significant reduction in AMPA/NMDA ratio in *Xkr8* cKO mice compared to wild-type controls (Fig 6I and J). Because the AMPA/NMDA ratio of CA1 pyramidal neurons is known to increase across early postnatal development (from about 1.2 to 2.5), in part due to the disappearance of transient, NMDAR-only synapses (Basilico *et al*, 2019), the reduction seen in *Xkr8* cKO mice suggests a failure of synaptic maturation. The immaturity of the synapses is further supported by significantly reduced eEPSC/sEPSC ratio in *Xkr8* cKO mice, which indicates impaired response of *Xkr8* cKO neurons to the stimulation (Fig 6H). Finally, we examined presynaptic properties of excitatory CA1 Schaffer collateral inputs by measuring paired-pulse ratio (PPR), a measure sensitive to the probability of action potential-dependent neurotransmitter release. PPR was significantly increased in *Xkr8* cKO mice when compared to wild-type controls (Fig 6K and L). indicative of a lower excitatory neurotransmitter release probability. PPR at excitatory CA1 Schaffer collateral synapses has been shown to remain constant across development (Hsia *et al*, 1998), and at present, the mechanistic link between defective axonal pruning and increased pair-pulsed facilitation is not clear. Taken together, these data point to a wide range of functional synaptic connectivity deficits associated with deficient axonal pruning, of which a subset are consistent with a failure in the functional maturation of excitatory synapses.

Inter-hemispheric cortical hyperconnectivity in Xkr8 cKO

Finally, we investigated the impact of deficient Xkr8-dependent PtdSer exposure during development on global brain connectivity in adulthood using resting state functional magnetic resonance imaging (rsfMRI) (Bijsterbosch *et al*, 2020; Grandjean *et al*, 2020). Voxel-wise temporal correlations in blood oxygen level dependent (BOLD) fMRI signal in lightly anesthetized mice has been used to estimate long-range functional neuronal connectivity in an unbiased and global manner (Liska *et al*, 2018). To assess whether deficits in the axonal pruning of cortical pyramidal neurons in *Xkr8* cKO mice might be associated with global changes in functional connectivity, we assessed brain-wide correlations in BOLD activity by seed-based rsfMRI analysis centered on cortical structures. This analysis revealed a significant increase in inter-hemispheric functional synchronization in *Xkr8* cKO mice compared to wild-type controls in all cortical seed areas examined, including anterior cingulate cortex, motor cortex, somatosensory cortex, and hippocampus (Fig 7A and B; Appendix Fig S5A and B). Increased inter-hemispheric functional connectivity also extended to forebrain regions with high reciprocal connectivity with cortex, such as dorsal striatum, but not to other sub-cortical brain areas such as hypothalamus or brainstem (Fig 7A and B) consistent with the selective ablation of Xkr8 in cortical and hippocampal excitatory neurons in our cKO mice. Finally, we complemented the seed-based approach with an unbiased, network-based statistical analysis of brain-wide signal synchronization (Pagani *et al*, 2019). Network-based statistical analysis revealed significant bilateral increases in intra-hemispheric connectivity among a large fraction of cortical brain regions and between selected cortical and subcortical brain regions (Appendix Fig S5B). These findings could be the result of a failure in axonal pruning of both inter- and intra-hemispheric long-range cortical connections in *Xkr8* cKO mice. Alternatively, increased intra-hemispheric synchronization could be an indirect consequence of increased inter-hemispheric functional connectivity between common cortical inputs (Swanson *et al*, 2017).

Discussion

We have shown here that Xkr8 phospholipid scramblase is developmentally regulated during the period of synaptic pruning and is required for appropriate sculpting of developing brain circuitry. Knockout of *Xkr8* in cortical excitatory neurons led to a significant increase in excitatory axonal inputs during the first postnatal week (Fig 3J) and a deficit in cortico-cortical and cortico-spinal axonal pruning (Fig 5). Notably, we found that *Xkr8* cKO animals did not show any change in the density of boutons along axonal shafts, nor of the density of spines along excitatory dendritic shafts (Fig 3C and E). These findings argue for a selective role of Xkr8-dependent PtdSer exposure in the elimination of entire axons, rather than the elimination of individual synaptic structures, and support the existence of distinct molecular processes controlling axonal and synaptic pruning that are known to occur in parallel during the first postnatal week in the mammalian cortex (Portera-Cailliau *et al*, 2005). This distinction was further strengthened by our observation that Xkr8-dependent PtdSer exposure and axonal pruning occurred before the formation of the majority of excitatory synapses as measured by the density of dendritic spines (Fig 3E). Nevertheless, we did observe a significant impact of Xkr8 on the maturation of the size of excitatory axonal boutons. While hippocampal excitatory bouton size decreased gradually in wild-type control animals from P8 to P28, this decrease was absent in *Xkr8* cKO mice (Fig 3A and B). The difference in bouton size appears gradually as axons develop in the absence of Xkr8, even though Xkr8 expression is significantly downregulated after birth (Fig 1A). This may indicate that Xkr8 has additional functions beyond the elimination of wholesale axons that persist despite a decrease in its expression during the second and third postnatal weeks. Otherwise, its role during the perinatal period may lead to a different developmental projectory, in which varicosity size is no longer adjusted as it is in wild-type animals.

Although the density of excitatory cortical axonal inputs was significantly increased at P8 in *Xkr8* cKO mice, this difference was no longer apparent at P28 (Fig 3J). The transient nature of the phenotype appeared to be driven by a rapid increase in vGluT1^{+} puncta in wild-type mice, which rose significantly to match cKO levels by P28 (Fig 3J). In addition to increased density of vGluT1^{+} puncta, *Emx1*::Cre-driven *Xkr8* cKO also led to the increase in vGluT2^{+} axons in cortex, suggesting that PtdSer-exposure may act in a cell non-autonomous manner to influence neuronal maturation.

© 2023 The Authors

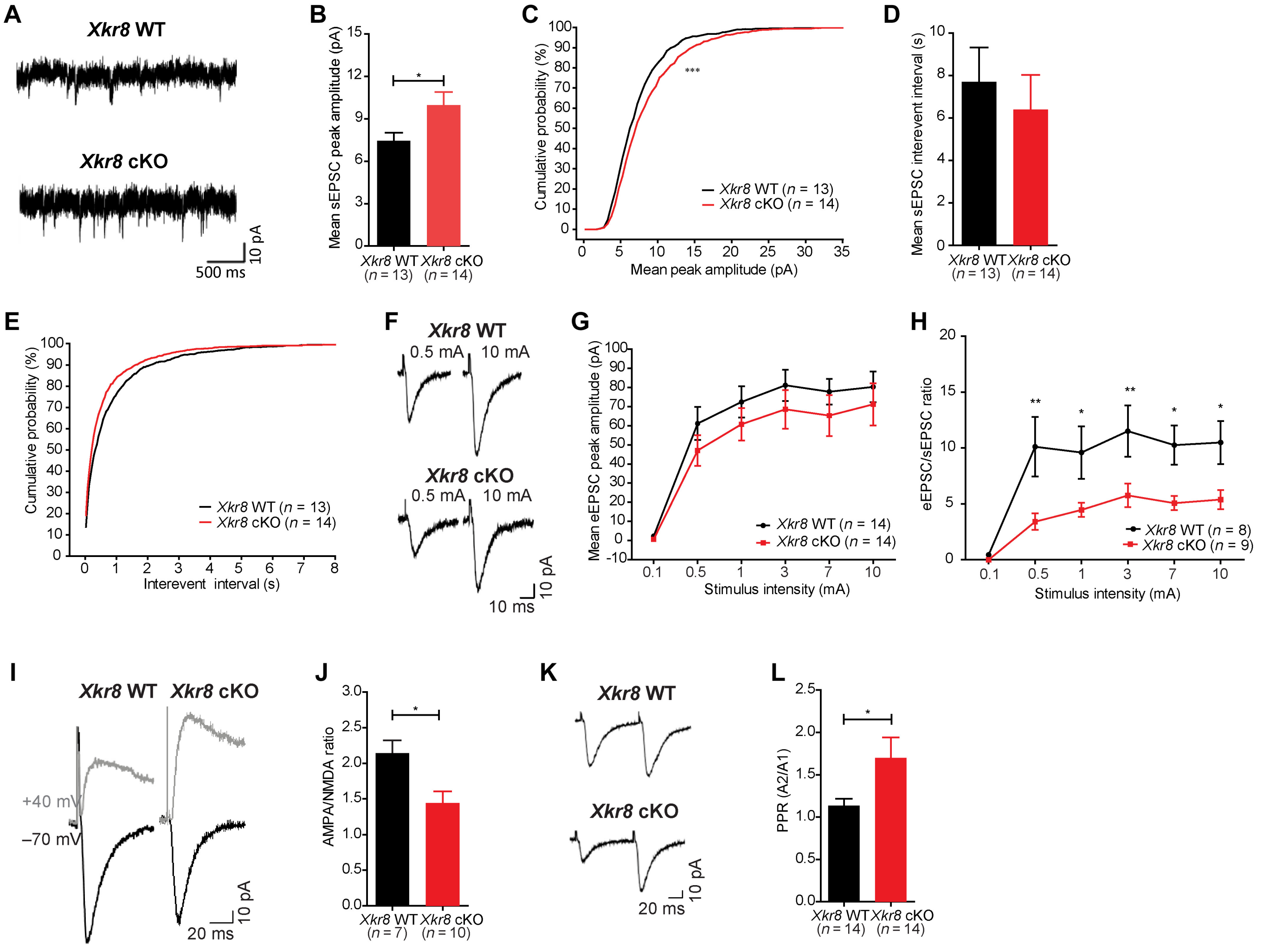

Figure 6. Electrophysiological alterations in *Xkr8* cKO hippocampus.

A Sample traces of spontaneous excitatory postsynaptic currents (sEPSC) of *Xkr8* WT and cKO neurons in acute hippocampal slices.

B–E *Xkr8* cKO neurons had increased mean sEPSC amplitude (B, C), but no changes in sEPSC frequency (D, E; $P = 0.58$).

F–H Evoked excitatory postsynaptic currents (eEPSC) of *Xkr8* cKO neurons showed a trend to decrease ($P = 0.08$) (F, G) and the ratio of eEPSC and sEPSC amplitudes was lower in Xkr8 knockout neurons (H).

I–L *Xkr8* cKO neurons had reduced of AMPA/NMDA amplitude ratio (I, J) and increased paired-pulse ratio (PPR; K, L).

Data information: Data were analyzed by two-tailed Student's *t*-test (B, D, J, L), Kolmogorov–Smirnov two-sample test (C, E) or two-way repeated measures ANOVA (H); $n = 7$–14 neurons per genotype. Data presented as mean ± SEM; $^*P < 0.05$, $^{**}P < 0.01$, $^{***}P < 0.001$.
Source data are available online for this figure.

Interestingly, exposed PtdSer has been shown to be cleaved by phospholipases to generate the chemotactic signal lysoPtdSer and we speculate that a lack of such a cell non-autonomous chemotactic role of PtdSer may explain the phenotypes we see beyond vGlut1$^+$ synapses in our mice (Frasch & Bratton, 2012; Blankman *et al*, 2013).

Our identification of Xkr8 as a selective regulator of developmental axonal elimination allowed us for the first time to begin to explore the functional consequences of axonal pruning on mature brain circuitry. In the neuromuscular junction, for example, where axonal pruning has been studied in some detail, the elimination of inputs is associated with a strengthening of the remaining inputs, and interventions that interfere with this process block the functional maturation of synaptic inputs (Tapia *et al*, 2012; Darabid *et al*, 2013, 2014; Neniskyte & Gross, 2017). While the overall profile of synaptic changes observed in *Xkr8* cKO mice does not appear to be the result of a single synaptic deficit, there may be links between the pre- and post-synaptic changes observed. For example, the reduced eEPSC AMPA/NMDA ratio seen in acute brain slices from mature *Xkr8* cKO mice (Fig 6J) resembles that seen in wild-type mice during the early postnatal period (Hsia *et al*, 1998) and points to a failure in the maturation of excitatory synapses in the knockout. At the same time, the apparent existence of a subset of spontaneously active excitatory synapses with exaggerated synaptic strength in cKO mice (Appendix Fig S4) suggests that Xkr8-dependent processes are also required for maintaining a

 © 2023 The Authors

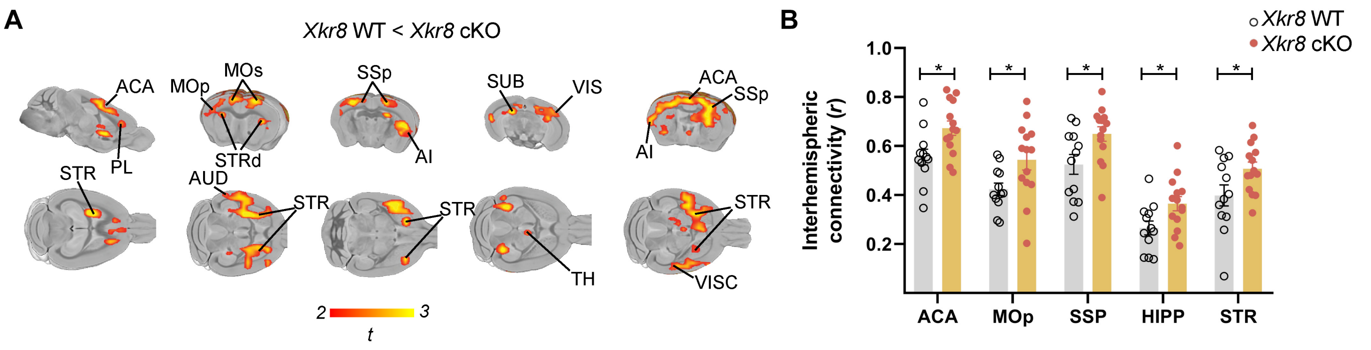

Figure 7. Increased global functional connectivity in *Xkr8* knockout.

A Brain regions with significantly increased functional connectivity in *Xkr8* cKO mice compared to *Xkr8* WT mice as identified by seed-based mapping of rsfMRI networks (t-test, $P < 0.05$ FWE cluster-corrected, with cluster-defining threshold of $t_{24} > 2.07$, $P < 0.05$; for seed placement see Appendix Fig S5). Colored regions represent the areas of the brain exhibiting increased functional connectivity in *Xkr8* cKO compared to *Xkr8* WT brains; color scale represents the level of functional correlation; abbreviations specify anatomical designations of identified regions.

B Inter-hemispheric connectivity in representative brain volumes of interest in *Xkr8* WT and cKO mice. Regional quantification was performed by computing regional fMRI correlation between the right and left hemisphere. The analysis revealed increased inter-hemispheric connectivity in *Xkr8* cKO brains compared to *Xkr8* WT (two-tailed Student's t-test, each dot represents an individual mouse, n = 12–14 per genotype; data presented as mean ± SEM, $^*P < 0.05$).

Data information: ACA: anterior cingulate cortex, AI: agranular insular area, AUD: auditory areas, HIPP: hippocampus, MOp: primary motor area, MOs: secondary motor area, PL: prelimbic area, SSp: primary somatosensory area, STR: striatum, STRd: striatum dorsal region, SUB: subiculum, TH: thalamus, VIS: visual areas, VISC: visceral area. Source data are available online for this figure.

homeostatic balance between weak and strong synaptic contacts. Finally, the increased paired-pulse facilitation (Fig 6L) may be linked to the persistence of silent synapses lacking AMPA receptors as indicated by the low AMPA/NMDA ratio and that can mimic low pre-synaptic neurotransmitter release (Gasparini *et al*, 2000; Crawford & Mennerick, 2012; Basilico *et al*, 2019). Although other explanations, including depotentiated pre-synaptic calcium dynamics or release machinery could also underlie the phenotype, the increased size of excitatory boutons in cKO mice points to a potentiation rather than a depotentiation of pre-synaptic release machinery (Murthy *et al*, 2001; Knodel *et al*, 2014) and leads us to favor a post-synaptic origin of the pair-pulsed facilitation deficit. More will need to be done to understand the cell autonomous and non-autonomous effects of Xkr8-dependent axonal remodeling on circuit maturation to gain a more precise picture of its role in circuit function.

The role we find for Xkr8 scramblase activity in axonal pruning is consistent with previous studies implicating PtdSer in the elimination of neuronal processes under both physiological and pathological conditions. PtdSer has been shown to be locally exposed on synapses in different models of neurodegeneration (Shacham-Silverberg *et al*, 2018) and on the tips of rod outer segments during diurnal phagocytosis in the retina (Ruggiero *et al*, 2012). It has also been associated with focal apoptosis or 'synaptosis' coupled with caspase-3 activation under a variety of neurodegenerative conditions (Mattson *et al*, 1998). Moreover, overexpression of a PtdSer scramblase promoted caspase-dependent dendritic and axonal degeneration in flies (Williams *et al*, 2006; Sapar *et al*, 2018) and caspase-dependent PtdSer exposure was found to be associated with the elimination of hippocampal and retinogeniculate synapses (Scott-Hewitt *et al*, 2020). Finally, caspase-3 activation is required for the regulation of dendritic plasticity (Erturk *et al*, 2014), which demonstrates that it can be involved in neuronal remodeling even in the absence of apoptosis, although it is not known if this is associated with PtdSer exposure.

It is important to note that several studies have implicated proteins of the complement system in synaptic pruning (Stevens *et al*, 2007; Schafer *et al*, 2012) and one of the complement opsonins, C1q, has been identified as a bridging molecule that recognizes PtdSer (Paidassi *et al*, 2008) suggesting a possible link between our findings and those from mice lacking C1q. For example, recent evidence suggests that C1q-bound synapses are enriched for externalized PtdSer and activated caspase-3 (Gyorffy *et al*, 2018). Therefore, PtdSer exposure by Xkr8 might provide an *eat-me* signal that is bound by complement opsonins and subsequently recognized by complement receptors. Moreover, pruning of retinal projections has been shown to require MerTK (Chung *et al*, 2013), a receptor for PtdSer (Lemke, 2019), further supporting a link between Xkr8 activation and axonal pruning. Importantly, microglial adhesion G protein-coupled receptor ADGRG1/GPR56 has been shown to bind to PtdSer on presynaptic elements to mediate synaptic pruning (Li *et al*, 2020), but its role in wholesale axonal pruning remains to be defined.

It is important to note that alternative mechanisms of Xkr8 involvement in axonal elimination may also exist, spanning beyond the exposure of PtdSer. For example, Xkr8 is known to interact with extracellular matrix metalloproteinase inducer Basigin (Sakuragi *et al*, 2021) or synaptic glycoprotein neuroplastin (Suzuki *et al*, 2016). As Basigin is known to mediate neuron–glia interaction (Curtin *et al*, 2007), the role of Xkr8 in neurodevelopment may not specifically require axonal exposure of PtdSer. Another Xkr8 partner neuroplastin regulates calcium homeostasis, synapse formation, and synaptic plasticity (Ilic *et al*, 2021), further supporting the role of Xkr8 in developing brain. However, further investigations are required to define how such interactions are related to or independent of the scramblase activity of Xkr8.

In conclusion, the identification of Xkr8 as a limiting factor for PtdSer exposure in the postnatal brain tissue and its selective involvement in promoting axonal pruning offers a powerful tool to enable investigations aimed at identifying the neural mechanisms that distinguish axons destined to be eliminated from those that survive and, on the other hand, at understanding the evolutionary rationale behind the programmed elimination of neuronal connections during mammalian brain development.

Materials and Methods

Experimental design

Immunofluorescence (IF) data presented here were obtained from organotypic hippocampal slices (at least four independent cultures) and fixed brain samples (5–6 animals per age group and per genotype). IF data were analyzed using nested factor design. Electrophysiology data were obtained from 13 to 14 neurons of each genotype for sEPSC, eEPSC, and PPR experiments, while AMPA/NMDA ratio was measured in 8–10 neurons of each genotype. Resting state fMRI measurements were done in 12–14 animals of each genotype. For all experiments, *Xkr8* WT and cKO animals were litter-matched. The data were analyzed blind to animal genotype.

Animals

C57BL/6J mice were obtained from local EMBL colonies. *Thy1*::EGFP mice (IMSR Cat# JAX:007788, RRID:IMSR_JAX:007788) were used in the heterozygous state. *Thy1*::EGFP;*Cx3cr1*::CreER;*RC*::LSL-tdTomato triple transgenic mouse line was used as previously described (Weinhard *et al*, 2018a). Mice were homozygous for *Thy1*::EGFP and heterozygous for *Cx3cr1*::CreER and *RC*::LSL-tdTomato. Cre-mediated recombination was induced by a single injection of 98% Z-isomer hydroxy-tamoxifen diluted in corn oil at 10 mg/ml (Sigma, 1 mg injected per 20 g of mouse weight) at P10 (Weinhard *et al*, 2018a). *Xkr8*-cKO/*Xkr8*-cKO;*Emx1*::Cre;*Thy1*::EGFP triple transgenic mice were obtained by crossing *Xkr8*-cKO/*Xkr8*-cKO mice kindly provided by Shigekazu Nagata (Suzuki *et al*, 2013a) with *Emx1*::Cre driver line (Iwasato *et al*, 2004) and *Thy1*::EGFP mice (IMSR Cat# JAX:007788, RRID: IMSR_JAX:007788). Mice were homozygous for *Xkr8*-cKO, either heterozygous or wild-type for *Emx1*::Cre and heterozygous for *Thy1*::GFP. All mice were on a C57BL/6J congenic background. Animal studies were conducted in accordance with Italian Law (DL 26/2014, EU 63/2010, Ministero della Sanità, Roma) and the recommendations of the Guide for the Care and Use of Laboratory Animals of the National Institutes of Health. All surgical procedures were performed under anesthesia.

Quantitative RT–PCR

The expression levels of *Xkr8*, *Xkr4*, *Xkr9*, and *Ano6* in developing mouse hippocampus were evaluated by quantitative RT–PCR. Total RNA was isolated from fresh hippocampal tissue of P0, P7, P28, P40, and P90 C57/Bl6 mice using Thermo Scientific GeneJET RNA Purification Kit according to the manufacturer's instructions. RNA integrity was verified using Agilent 2100 Bioanalyzer with RNA 6000 LabChip kit (RIN value of all samples were within 8.5–10). First-strand cDNA was synthesized by Thermo Scientific Maxima First Strand cDNA Synthesis Kit according to the manufacturer's instructions. RT–PCR was performed using 50 ng of cDNA as a template and Thermo Scientific Maxima SYBR Green/ROX qPCR Master Mix. The following primers were used: *mXkr8*: 5′-GCGAC GCCACAGCTCACACT-3′ and 5′-CCCCAGCAGCAGCAGGTTCC-3′; *mXkr4*: 5′-GCCAGTGACCGTGATCAGAA-3′ and 5′-TCCTTGTACTGC AGCCTTGG-3′; *mXkr9*: 5′-GGAAGGCTGCCCGCAACTCA-3′ and 5′-TGGGCCAGAGTCCTCGGAGAA-3′ (Suzuki *et al*, 2014); *mAno6*: 5′-TCTGGTGCTGGAAAACTTTGA-3′ 5′-TCGGGCTTCCCGTTAAATTC-3′; *mGapdh* (internal control): 5′-AGCAGGCATCTGAGGGCCCA-3′ and 5′-GAGAGCAATGCCAGCCCCGG-3′ (Timur *et al*, 2015). To evaluate the change of expression during development, $-\Delta\Delta Ct$ values were calculated using *mGapdh* as a sample internal reference and P90 values as expression reference. To compare *Xkr8* and *Xkr4* expression and to compare *Xkr8*, *Xkr4*, and *Ano6* expression in *Xkr8* WT and cKO mice at P0, $-\Delta Ct$ values were calculated using *mGapdh* as a sample internal reference.

In situ hybridization

Brains were collected, fresh frozen in OCT and sectioned at 20 μm onto Superfrost Plus slides. ISH was performed (Mirabeau *et al*, 2007) using *in vitro* transcribed digoxigenin (DIG)-labeled probes from the *mXkr8* exon 3 sequence. Briefly, sections were fixed in 4% paraformaldehyde, digested with proteinase K for 5 min, acetylated, and hybridized with the probes in 50% formamide, 5× SSC, 5× Denhardt's solution, 500 μg/ml salmon sperm DNA, and 250 μg/ml tRNA overnight at 56.5°C. After post-hybridization washes with 50% formamide, 2× SSC at 56.5°C, and with 2× SSC at room temperature, sections were blocked and incubated overnight with anti-digoxigenin-AP (1:1,000, Roche Cat# 11093274910, RRID: AB_2734716). Signal was detected using NBT/BCIP chromogenic substrates.

Immunofluorescence labeling of developing brain

Xkr8$^{flx/flx}$;+/+;*Thy1*::EGFP and *Xkr8*$^{flx/flx}$;*Emx1*::Cre/+;*Thy1*::EGFP mice at P8, P15, P28, and P40 mice were anesthetized with intraperitoneal injection of 2.5% Avertin (Sigma-Aldrich) and perfused transcardially with 4% paraformaldehyde (PFA). Brains were removed and post-fixed in 4% PFA overnight at 4°C. Coronal 50 μm sections were cut on a vibratome (Leica Microsystems) and permeabilized in 20% normal goat serum with 0.4% Triton X-100 in PBS for 2 h at room temperature.

To visualize Xkr8 protein expression, the sections were immunolabeled overnight at 4°C with primary antibodies using either rabbit anti-Xkr8 (1:50, Thermo Fisher Scientific Cat# PA5-46669, RRID: AB_2577288; recognizes both full-length and cleaved Xkr8) or rabbit anti-Xkr8 (1:50, Thermo Fisher Scientific Cat# PA5-72820, RRID: AB_2718674; recognizes only full-length Xkr8) followed by secondary antibody goat anti-rabbit AF594 (1:400, Thermo Fisher Scientific Cat# A-11011) in PBS with 5% donkey serum for 2 h at room temperature.

To investigate caspase-3 activation, active caspase-3 was immunodetected by overnight incubation at 4°C with primary antibodies (rabbit anti-active-caspase-3 1:100, Cell Signaling Technology Cat#

 © 2023 The Authors

9664, RRID:AB_2070042) followed by secondary antibodies (goat anti-rabbit AF546, Thermo Fisher Scientific Cat# A-11071, RRID: AB_2534115, at 1:400) in PBS with 5% donkey serum for 2 h at room temperature.

To analyze the densities of vGluT1^{+} and vGluT2^{+} inputs, vGluT1 and vGluT2 were immunodetected and EGFP was immunoenhanced by overnight incubation at 4°C with primary antibodies (rabbit anti-vGluT1 1:500, Synaptic Systems Cat# 135302, RRID:AB_887877; mouse anti-vGluT2 1:500, Abcam Cat# ab79157, RRID:AB_1603114; chicken anti-GFP 1:1,000, Aves Labs Cat# GFP-1020, RRID: AB_10000240) followed by the incubation with secondary antibodies (goat anti-rabbit AF546, Thermo Fisher Scientific Cat# A-11071, RRID:AB_2534115; goat anti-mouse AF647, Thermo Fisher Scientific Cat# A-21235, RRID:AB_2535804; and goat anti-chicken AF488, Thermo Fisher Scientific Cat# A-11039, RRID:AB_2534096; all at 1:400) in PBS with 5% goat serum with 0.4% Triton X-100 for 2 h at room temperature.

To visualize internalized axonal and synaptic material within microglia cells, Iba1, pan-axonal material, vGluT1 and PSD95 were immunodetected by overnight incubation at 4°C with primary antibodies (rabbit anti-Iba1, FUJIFILM Wako Shibayagi Cat# 019-19741, RRID:AB_839504 at 1:500; mouse anti-SMI312, BioLegend Cat# 837904, RRID:AB_2566782 at 1:500; guinea pig anti-vGluT1, Synaptic Systems Cat# 135304, RRID:AB_887878 at 1:500; mouse anti-PSD95, Synaptic Systems Cat# 124011, RRID:AB_10804286 at 1:100) followed by the incubation with secondary antibodies (goat anti-rabbit AF594, Thermo Fisher Scientific Cat# A-11012, RRID: AB_2534079; goat anti-mouse AF647, Thermo Fisher Scientific Cat# A-21235, RRID:AB_2535804; goat anti-guinea pig AF488, Thermo Fisher Scientific Cat# A-11073, RRID:AB_2534117, all at 1:500) in PBS with 5% goat serum with 0.4% Triton X-100 for 2 h at room temperature.

Immunolabeled sections were counterstained with DAPI, mounted with Mowiol, and imaged on Leica TCS SP8 confocal microscope using 63×/1.4NA oil-immersion objective at 46 nm lateral pixel size with an axial step of 130 nm.

IF image analysis

Analysis of confocal image stacks was performed using ImageJ (RRID:SCR_003070) open source image analysis software blind to animal genotype.

To quantify the density active caspase-3 structures, images were analyzed in 3D Image Suite. After subtracting the background and applying a Gaussian blur 3D filter the low and high threshold of analyzed signal was defined and the images were 3D segmented. The number of objects in the field of view was obtained by the Measure 3D function and normalized to the volume of the image.

To identify boutons on axonal fragments a maximum intensity *z*-projection was created, background was subtracted, and Gaussian blur filter was applied. Boutons were identified using Find Maxima function with noise tolerance set manually. Output image of Maxima With Tolerance were quantified with Analyze Particles function to obtain the number and size of boutons. The length of axonal fragments was measured and the density of boutons on each axonal fragment was calculated. The number of spines on the dendritic fragments was counted manually in maximum intensity *z*-projections and the density of dendritic spines was defined by the length of analyzed fragment.

To evaluate the densities of vGluT1^{+} and vGluT2^{+} particles, a maximum intensity *z*-projection was created, background was subtracted, and a Gaussian blur filter was applied. vGluT1 and vGluT2 particles were identified by Find Maxima function with noise tolerance set to 10 and output type defined as Maxima With Tolerance. Output image represented vGluT1 and vGluT2 signal as puncta that were counted using Analyze Particles function and normalized to image area.

To evaluate the internalization of SMI312^{+}, vGluT1^{+}, and PSD95^{+} neuronal material by Iba1^{+} microglia cells, a *z*-stack images encompassing the whole microglial cell were acquired with 0.3 μm *z*-step. After subtracting the background and applying Gaussian blur 3D filter, the 3D surface of Iba1^{+} microglial cell was obtained in 3D ROI manager. SMI312^{+}, vGluT1^{+}, and PSD95^{+} the particles were identified by 3D segmentation. For SMI312^{+} analysis, large fragments of traversing axons were removed by Analyze Particles function set to include small particles only. The volume of SMI312^{+}, vGluT1^{+}, and PSD95^{+} within the 3D surface of Iba1^{+} microglia was quantified using Measure 3D function and normalized to the volume of microglia.

Preparation of hippocampal slice culture

Organotypic hippocampal slice cultures were prepared using the air/medium interface method (Stoppini *et al*, 1991). Briefly, mice were decapitated at P3 and hippocampi were dissected out in cold dissecting medium (Hank's Balanced Salt Solution, 100 U/ml penicillin/100 μg/ml streptomycin, 15 mM HEPES, and 0.5% glucose). Hippocampi were transversely cut into 300 μm thickness sections using a McIlwain tissue chopper. Slices were placed onto PTFE cell culture inserts (Millipore) in pre-warmed six-well plates with each well containing 1.2 ml of maintaining medium (50% Minimum Essential Medium, 25% Basal Medium Eagle, 25% horse serum, 100 U/ml penicillin/100 μg/ml streptomycin, 2 mM GlutaMAX, 0.65% glucose, 7.5% sodium bicarbonate). Cultures were maintained in an incubator at 35°C and 5% CO_2 for up to 28 days *in vitro*. Medium was replaced 24 h after preparation and then every 2–3 days.

Live labeling of PtdSer exposure

To define the localization of PtdSer exposure live *Thy1*::EGFP organotypic hippocampal slices at 16–19 days *in vitro* were incubated with Cy5-conjugated Annexin V (Biovision) diluted 1:50 (~2.4 μg/ml) in maintaining medium for 16 h. The slices were then washed three times with maintaining medium to eliminate any unbound Annexin V and fixed in 4% paraformaldehyde (PFA) in phosphate buffer (30 mM KH_2PO_4, 100 mM Na_2HPO_4). After quenching with 30 mM glycine in phosphate-buffered saline (137 mM NaCl, 2.7 mM KCl, 10 mM Na_2HPO_4, 1.8 mM KH_2PO_4) and rinsing with PBS, slices were mounted with Mowiol and imaged as described above. To evaluate PtdSer exposure in *Xkr8* WT and *Xkr8* cKO organotypic slices, the same volume of hippocampal tissue was imaged in all samples. The total fluorescence intensity of bound annexin V was measured using ImageJ (RRID:SCR_003070) software and normalized to the *z* stack volume to express it as fluorescence units. To

© 2023 The Authors

investigate Annexin V localization on *Thy1*::GFP neurons, *z* stacks were acquired to encompass the full fragment of analyzed axon or dendrite. Annexin V structures, axons with boutons and dendrites with spines were reconstructed in 3D using Imaris software (Bitplane). Any Annexin V signal outside the reconstructed GFP^+ surface was eliminated from the analysis. Total fluorescence was measured for each Annexin V structure, which were then visually evaluated for their location: on the shaft, on the dendritic spine, or on the axonal bouton. Then, total cumulative fluorescence for Annexin V structures located on the shafts, spines, and boutons was compared.

Dendrite Sholl analysis

Neurons expressing GFP in P28 *Xkr8* WT and cKO mice brain sections were immunoenhanced as described above. Mosaic images of GFP^+ pyramidal neurons in the CA1 region of the hippocampus were acquired on Leica TCS SP8 resonant scanner confocal microscope with 63×/1.4NA oil immersion objective at 1.25 optical zoom, pixel size 0.144 × 0.144 μm^2, *z*-axial step size 1 μm, using Leica LasX SPE and LasX navigator software. Images were processed and analyzed using ImageJ (RRID:SCR_003070) software. Dendritic arborizations of each neuron were traced from the soma center as origin along the full length of the apical dendrite using the Simple Neurite Tracer plugin (https://imagej.net/SNT) and total dendrite length was measured. The traces were converted into 8 bit images and Sholl analysis was performed using the Sholl Analysis feature provided by the SNT plugin with the center of the soma as a starting point and 10 μm radius step size.

Cortical cell density analysis

Cell density in whole somatosensory cortex and in layer 4 was defined by counting DAPI-stained nuclei in coronal cortical sections of *Xkr8* WT and *Xkr8* cKO mice at P8 and P28. Images were collected on Leica SP8 resonant scanner confocal microscope with 20× 0.75NA air objective. The images were quantified on ImageJ software (RRID:SCR_003070). The thickness of the cortex was defined using Measure function. The same analysis was performed both for the whole cortex and for manually cropped layer 4.

Modified Palmgren silver staining

To visualize corticospinal tracts, modified Palmgren silver staining was used (Goshgarian, 1977). Coronal medulla sections from P8 *Xkr8* WT and cKO mice were placed on the adhesion microscope slides, air dried, and dehydrated through graded 70% to absolute ethanol and rehydrated back to distilled water for uniform staining. For better impregnation of terminal axon arbors, sections were treated with 0.15 M 2-amino-2-methyl-1-propanol solution (adjusted with nitric acid to 5.0 pH) for 7 min and washed in three changes of distilled water, each 1.5 min. Then the sections were impregnated in 10% aqueous silver nitrate for 45 min at 37°C, transferred without rinsing to freshly prepared 2% sodium borate for 10 s, agitated and rinsed in distilled water until the precipitate was removed. The sections were developed in 2% fresh sodium borate solution containing 0.05% hydroquinone and 5% sodium sulfite for 15 min at 37°C and rinsed three times in 50% ethanol, each 1.5 min. Then, the sections were toned with 0.5% gold chloride solution for 5 min and rinsed with distilled water. The staining was intensified with 0.5% oxalic acid in 50% ethanol for 2 min at 37°C. Finally, the sections were rinsed in distilled water, fixed in 5% sodium thiosulphate for 20 s, rinsed, dried, cleared, and mounted with Mowiol. The bright-field imaging was performed on Olympus AX70 wide-field microscope with 60× 1.25NA oil objective. The number of axons was defined by using Find Maxima function on ImageJ software (RRID:SCR_003070).

Corpus callosum CTB labeling and analysis

Cholera toxin subunit B (CTB) back-labeling and quantitation was performed as described by De León Reyes *et al* (2019) with some modifications. P30-32 age mice were anesthetized with ketamine/xylazine (100 and 10 mg/kg, respectively, i.p. injection) and placed in a stereotactic frame (Narishige Instruments); isoflurane in oxygen (1–2%) was administered to maintain anesthesia. The skull surface was exposed and 500 nl of CTB-647 (0.5% in PBS, Life Technologies) was pressure-injected into the corpus callosum (co-ordinates: AP = −1.4, ML = 0.70, and DV = −1.70 with an angle of 180°) at the rate of 50 nl/min using a glass capillary. After the CTB-647 injection, the capillary was left in position for 10 min and then retracted. All mice received a subcutaneous injection of Carprofen (Rymadil 5 mg/kg) as surgical analgesia. After allowing CTB migration for 48 h, mice were transcardially perfused with 4% PFA in PBS. Brains were removed from the skull and post-fixed overnight in 4% PFA at 4°C. Brains were cryoprotected with 30% sucrose and 40 μm coronal sections cut on a cryostat. For quantitative analysis, coronal sections corresponding to −1.23 to −1.5 mm AP were used. Images were acquired with an inverted microscope (Thunder, Leica Microsystems) using 20×/0.8 NA objective and an sCMOS camera. Images were acquired using 1 μm optical thickness with LAS X software (Leica Microsystems). Mosaics were generated by merging several individual frames, using a spatial overlap of 15% on LAS X software. Quantification of CTB^+ cells was performed manually using ImageJ on images from *z* stacks using DAPI and CTB staining. S1 and S2 regions of the somatosensory cortex were demarcated by the pattern of CTB back-labeling. Fifty nuclei were randomly selected using the 'multi-point tool' and the proportion of CTB^+ cells among nuclei was calculated in layer 4 of the S1 and S2 regions. Data are presented as the percentage of CTB^+ cells out of selected $DAPI^+$ cells.

In vitro electrophysiology

Acute hippocampal slices were prepared from *Xkr8*-cKO/*Xkr8*-cKO;*Emx1*::+/+;*Thy1*::EGFP and *Xkr8*-cKO/*Xkr8*-cKO;*Emx1*::Cre/+;*Thy1*::EGFP male mice at P40. Animals were decapitated under halothane anesthesia and whole brains were rapidly immersed for 5–10 min in chilled artificial cerebrospinal fluid (ACSF: 125 mM NaCl, 2.5 mM HCl, 2 mM $CaCl_2$, 1 mM $MgCl_2$, 1.25 mM NaH_2PO_4, 1.1 mM glucose, 2.6 mM $NaHCO_3$) with 250 mM glycerol. Brains were sectioned into 250-μm-thick slices at 4°C, using a vibratome (DSK, Dosaka EM). Slices were placed in a chamber filled with oxygenated ACSF to recover for 1 h at room temperature (RT). All recordings were performed at RT on slices submerged and perfused with ACSF with 10 μM bicuculline. CA1 pyramidal neurons were

 © 2023 The Authors

visualized with an upright Axioscope microscope (Zeiss) and were patched in whole-cell configuration. Borosilicate glass micropipettes (3.5–4.5 MΩ) were filled with an intracellular solution (135 mM $CsMetSO_4$, 10 mM HEPES, 2 mM MgATP, 0.3 mM NaGTP, 2 mM Qx314 bromide, 2 mM $MgCl_2$, 0.4 mM $CaCl_2$, 5 mM BAPTA). Bipolar theta micropipettes (filled with ACSF) were used for stimulation and placed in *stratum radiatum* near the CA1 area over the Schaffer-commissural afferent fibers. Membrane currents were recorded with a patch-clamp amplifier (Axopatch 200A, Molecular Devices) and were filtered at 2 kHz, digitized (10 kHz) and acquired with Clampex 10 software (Molecular Devices). To record sEPSCs, each neuron was clamped at −70 mV for 10 min. Recorded signals were low-pass filtered at 1 kHz and analyzed using Clampfit 10.4 software (Molecular Devices). sEPSC were identified on the basis of a template created for each neuron using 50–70 single events for each trace. All events recognized through the template search function were visualized, identified, and accepted by manual analysis. Input–output curves of eEPSCs were recorded by sequentially stimulating Schaffer collateral fibers at different intensities (0.1, 0.5, 1, 3, 7 and 10 mA) using a paired-pulse protocol (0.1 ms duration of the stimulus, 50 ms interval between two consecutive stimuli and 10 s interval between pairs). Paired pulse ratio was determined at 0.5 mA intensity of stimulation. To measure AMPA/NMDA ratio, the stimulation intensity was chosen to evoke a half-maximal response to the first stimulus while maintaining the cell at −70 mV. The cell was then depolarized to +40 mV and the NMDA component was measured. The responses of each neuron were analyzed in 18 sweeps (3 separate concatenated recordings) and the peak amplitude was estimated after averaging the traces. NMDA response was evaluated by measuring response amplitude at the 25 ms time point after the peak of AMPA response ($t_{\mathrm{AMPA}\,+\,25\ \mathrm{ms}}$). AMPA/NMDA ratio was determined as the ratio between the peak amplitude recorded at −70 mV (AMPA current) and $t_{\mathrm{AMPA}\,+\,25\ \mathrm{ms}}$ amplitude of the response recorded at +40 mV (NMDA current). Estimated EPSC reversal potentials were 8.8 ± 3.7 and 7.2 ± 1.4 mV (mean ± SEM) for *Xkr8* WT and *Xkr8* KO, respectively. Cells deviating more than 15 mV from this value were discarded from the analyses. The experiments were performed 1–8 h after slicing. The recordings were carried out blind to animal genotype.

Resting state functional magnetic resonance imaging

Adult male homozygous *Xkr8* cKO (n = 14) and age-matched control WT littermates (n = 12; 16–40 weeks) were imaged as previously described (Liska *et al*, 2018). Briefly, animals were anesthetized with isoflurane, intubated, and artificially ventilated. After surgery, isoflurane was discontinued and replaced with light halothane anesthesia (Sforazzini *et al*, 2014). Functional data acquisition commenced 30 min after isoflurane cessation. Functional images were acquired with a 7T MRI scanner (Bruker Biospin) as previously described (Liska *et al*, 2018; Gutierrez-Barragan *et al*, 2019), using a 72-mm birdcage transmit coil and a 4-channel solenoid coil for signal reception. Single-shot BOLD rsfMRI time series were acquired using an echo planar imaging (EPI) sequence with the following parameters: TR/TE 1000/15 ms, flip angle 30°, matrix 100 × 100, field of view 2.3 × 2.3 cm, 18 coronal slices, slice thickness 550 μm for 1,620 volumes, corresponding to a total acquisition time of 27 min.

Functional connectivity analysis

Resting state functional magnetic resonance imaging time-series were preprocessed as previously described (Bertero *et al*, 2018; Liska *et al*, 2018; Suetterlin *et al*, 2018; Pagani *et al*, 2019). The initial 25 volumes of the time series were removed to allow for T1 and gradient temperature equilibration effects. Data were then despiked, motion corrected, and spatially registered to a common reference template. Motion traces of head realignment parameters (3 translations + 3 rotations) and mean ventricular signal (corresponding to the averaged BOLD signal within a reference ventricular mask) were used as nuisance covariates and regressed out from each time course. All rsfMRI time series next underwent band-pass filtering within a frequency window of 0.01–0.1 Hz, and spatial smoothing with a full width at half maximum of 0.6 mm. No intergroup differences were observed in mean p_aCO_2 (*Xkr8* WT: 28.6 ± 7.6 mmHg; *Xkr8* cKO: 28.2 ± 6.2 mmHg; P = 0.9), p_aO_2 (*Xkr8* WT: 204.2 ± 8.12 mmHg; *Xkr8* cKO: 209.4 ± 19.4 mmHg; P = 0.4), and head motion as assessed with frame-wise displacement (*Xkr8* WT: 0.04 ± 0.007 mm; *Xkr8* cKO: 0.04 ± 0.009 mm; P = 0.8). rsfMRI connectivity alterations in *Xkr8* cKO mice were mapped using seed-based analysis in predefined volumes of interest (VOIs) (Pagani *et al*, 2019). Voxel-wise intergroup differences were mapped using a 2-tailed Student's t-test ($P < 0.05$ family-wise error [FWE] cluster-corrected, with cluster-defining threshold of $t_{24} > 2.07$, $P < 0.05$). To corroborate results of voxel-wise connectivity maps, we also calculated rsfMRI connectivity between 74 unilateral (37 right and 37 left) VOIs for each mouse and then we performed a t-test between genotypes for each edge separately ($t > 2.1$, $P < 0.05$). FWER correction was performed using 5,000 permutations ($P < 0.05$) as implemented in the network based statistics toolbox (Zalesky *et al*, 2010).

Quantification and statistical analysis

Statistica 10.0 software was used for statistical analysis. The data are presented as mean ± SEM. For the Sholl analysis of dendritic trees, curves represent mean intersection values ± SEM. Outliers were defined as exceeding three interquartile ranges and removed from the analysis. Organotypic slice data comparisons were performed by two-tailed Student's t-test. A nested design and mixed-model ANOVA was used for the analysis of IF, CTB and dendrite length data and included both fixed (age and genotype) and random (mice) effects. One-way ANOVA was used for qPCR data. Multiple comparisons were performed by Fischer LSD *post hoc* test. Electrophysiology data were analyzed using Origin 6 and GraphPad Prism 5 and SigmaPlot 12.3 software. Mean amplitude and frequency of sEPSCs, mean amplitude of eEPSCs, AMPA/NMDA ratio and PPR were compared using two-tailed Student's t-test. eEPSC/sEPSC ratios were compared using two-way repeated measures ANOVA and Fischer LSD *post hoc* test. Cumulative probability of peak amplitude of sEPSCs was compared using Kolmogorov–Smirnov two-sample test. Statistical analysis of rsfMRI was performed as described above. Threshold for significance levels was set at $P < 0.05$.

© 2023 The Authors

Data availability

Source Data microscopy images are deposited at BioImage Archive (accession No. S-BIAD678).

Expanded View for this article is available online.

Acknowledgements

We are grateful to Shigekazu Nagata (Kyoto University, Kyoto, Japan) for kindly providing us with *Xkr8* cKO mice. We thank Laetitia Weinhard (EMBL Rome, Italy) for her help in establishing the organotypic cultures. We thank the EMBL Microscopy and Laboratory Animal Resources Facilities for their essential support. UN has received funding from the European Union's Horizon 2020 research and innovation program under the Marie Skłodowska-Curie grant agreement No 705452, International Brain Research Organization Return Home Fellowship, and L'ORÉAL Baltic "For Women In Science" fellowship with the support of the Lithuanian National Commission for the United Nations Educational, Scientific and Cultural Organization (UNESCO) the Lithuanian Academy of Sciences; UN, UK, DP, ND and DD were funded by the European Regional Development Fund under grant agreement No 01.2.2-CPVA-V-716-01-0001 with the Central Project Management Agency (CPVA), Lithuania; CTG has received funding from EMBL and the European Research Council, ERC Advanced Grant COREFEAR No 341139. AG has received funding from IIT, the Simons Foundation (SFARI No 314688 and 400101), the Brain and Behavior Foundation (NARSAD, No 25861), and the European Research Council, ERC – DISCONN, No 802371. Open Access funding enabled and organized by Projekt DEAL.

Author contributions

Urte Neniskyte: Conceptualization; supervision; investigation; visualization; writing – original draft; writing – review and editing. **Ugne Kuliesiute:** Investigation. **Auguste Vadisiute:** Investigation; visualization. **Kristina Jevdokimenko:** Investigation. **Ludovico Coletta:** Investigation; visualization. **Senthilkumar Deivasigamani:** Investigation. **Daina Pamedytyte:** Investigation. **Neringa Daugelaviciene:** Investigation. **Daiva Dabkeviciene:** Formal analysis; visualization. **Emerald Perlas:** Investigation. **Aditya Bali:** Investigation. **Bernadette Basilico:** Investigation. **Alessandro Gozzi:** Supervision; funding acquisition. **Davide Ragozzino:** Supervision. **Cornelius T Gross:** Conceptualization; supervision; funding acquisition; writing – original draft.

Disclosure and competing interests statement

The authors declare that they have no conflict of interest.

References

Basilico B, Pagani F, Grimaldi A, Cortese B, Di Angelantonio S, Weinhard L, Gross C, Limatola C, Maggi L, Ragozzino D (2019) Microglia shape presynaptic properties at developing glutamatergic synapses. *Glia* 67: 53 – 67

Bertero A, Liska A, Pagani M, Parolisi R, Masferrer ME, Gritti M, Pedrazzoli M, Galbusera A, Sarica A, Cerasa A *et al* (2018) Autism-associated 16p11.2 microdeletion impairs prefrontal functional connectivity in mouse and human. *Brain* 141: 2055 – 2065

Bijsterbosch J, Harrison SJ, Jbabdi S, Woolrich M, Beckmann C, Smith S, Duff EP (2020) Challenges and future directions for representations of functional brain organization. *Nat Neurosci* 23: 1484 – 1495

Blankman JL, Long JZ, Trauger SA, Siuzdak G, Cravatt BF (2013) ABHD12 controls brain lysophosphatidylserine pathways that are deregulated in a murine model of the neurodegenerative disease PHARC. *Proc Natl Acad Sci USA* 110: 1500 – 1505

Chung WS, Clarke LE, Wang GX, Stafford BK, Sher A, Chakraborty C, Joung J, Foo LC, Thompson A, Chen C *et al* (2013) Astrocytes mediate synapse elimination through MEGF10 and MERTK pathways. *Nature* 504: 394 – 400

Crawford DC, Mennerick S (2012) Presynaptically silent synapses: dormancy and awakening of presynaptic vesicle release. *Neuroscientist* 18: 216 – 223

Curtin KD, Wyman RJ, Meinertzhagen IA (2007) Basigin/EMMPRIN/CD147 mediates neuron-glia interactions in the optic lamina of Drosophila. *Glia* 55: 1542 – 1553

Darabid H, Arbour D, Robitaille R (2013) Glial cells decipher synaptic competition at the mammalian neuromuscular junction. *J Neurosci* 33: 1297 – 1313

Darabid H, Perez-Gonzalez AP, Robitaille R (2014) Neuromuscular synaptogenesis: coordinating partners with multiple functions. *Nat Rev Neurosci* 15: 703 – 718

De León Reyes NS, Mederos S, Varela I, Weiss LA, Perea G, Galazo MJ, Nieto M (2019) Transient callosal projections of L4 neurons are eliminated for the acquisition of local connectivity. *Nat Commun* 10: 4549

Erturk A, Wang Y, Sheng M (2014) Local pruning of dendrites and spines by caspase-3-dependent and proteasome-limited mechanisms. *J Neurosci* 34: 1672 – 1688

Feng G, Mellor RH, Bernstein M, Keller-Peck C, Nguyen QT, Wallace M, Nerbonne JM, Lichtman JW, Sanes JR (2000) Imaging neuronal subsets in transgenic mice expressing multiple spectral variants of GFP. *Neuron* 28: 41 – 51

Frasch SC, Bratton DL (2012) Emerging roles for lysophosphatidylserine in resolution of inflammation. *Prog Lipid Res* 51: 199 – 207

Gasparini S, Saviane C, Voronin LL, Cherubini E (2000) Silent synapses in the developing hippocampus: lack of functional AMPA receptors or low probability of glutamate release? *Proc Natl Acad Sci USA* 97: 9741 – 9746

Goshgarian HG (1977) A rapid silver impregnation for central and peripheral nerve fibers in paraffin and frozen sections. *Exp Neurol* 57: 296 – 301

Grandjean J, Canella C, Anckaerts C, Ayrancı G, Bougacha S, Bienert T, Buehlmann D, Coletta L, Gallino D, Gass N *et al* (2020) Common functional networks in the mouse brain revealed by multi-centre resting-state fMRI analysis. *Neuroimage* 205: 116278

Gutierrez-Barragan D, Basson MA, Panzeri S, Gozzi A (2019) Infraslow state fluctuations govern spontaneous fMRI network dynamics. *Curr Biol* 29: 2295 – 2306

Gyorffy BA, Kun J, Torok G, Bulyaki E, Borhegyi Z, Gulyassy P, Kis V, Szocsics P, Micsonai A, Matko J *et al* (2018) Local apoptotic-like mechanisms underlie complement-mediated synaptic pruning. *Proc Natl Acad Sci USA* 115: 6303 – 6308

Hsia AY, Malenka RC, Nicoll RA (1998) Development of excitatory circuitry in the hippocampus. *J Neurophysiol* 79: 2013 – 2024

Ilic K, Mlinac-Jerkovic K, Sedmak G, Rosenzweig I, Kalanj-Bognar S (2021) Neuroplastin in human cognition: review of literature and future perspectives. *Transl Psychiatry* 11: 394

Innocenti GM (1981) Growth and reshaping of axons in the establishment of visual callosal connections. *Science* 212: 824 – 827

Innocenti GM, Price DJ (2005) Exuberance in the development of cortical networks. *Nat Rev Neurosci* 6: 955 – 965

Innocenti GM, Fiore L, Caminiti R (1977) Exuberant projection into the corpus callosum from the visual cortex of newborn cats. *Neurosci Lett* 4: 237 – 242

Iwasato T, Nomura R, Ando R, Ikeda T, Tanaka M, Itohara S (2004) Dorsal telencephalon-specific expression of Cre recombinase in PAC transgenic mice. *Genesis* 38: 130 – 138

 © 2023 The Authors

Knodel MM, Geiger R, Ge L, Bucher D, Grillo A, Wittum G, Schuster CM, Queisser G (2014) Synaptic bouton properties are tuned to best fit the prevailing firing pattern. *Front Comput Neurosci* 8: 101

Kurematsu C, Sawada M, Ohmuraya M, Tanaka M, Kuboyama K, Ogino T, Matsumoto M, Oishi H, Inada H, Ishido Y *et al* (2022) Synaptic pruning of murine adult-born neurons by microglia depends on phosphatidylserine. *J Exp Med* 219: e20202304

LaMantia AS, Rakic P (1990) Axon overproduction and elimination in the corpus callosum of the developing rhesus monkey. *J Neurosci* 10: 2156 – 2175

Lehrman EK, Wilton DK, Litvina EY, Welsh CA, Chang ST, Frouin A, Walker AJ, Heller MD, Umemori H, Chen C *et al* (2018) CD47 protects synapses from excess microglia-mediated pruning during development. *Neuron* 100: 120 – 134

Lemke G (2019) How macrophages deal with death. *Nat Rev Immunol* 19: 539 – 549

Li T, Chiou B, Gilman CK, Luo R, Koshi T, Yu D, Oak HC, Giera S, Johnson-Venkatesh E, Muthukumar AK *et al* (2020) A splicing isoform of GPR56 mediates microglial synaptic refinement via phosphatidylserine binding. *EMBO J* 39: e104136

Liska A, Bertero A, Gomolka R, Sabbioni M, Galbusera A, Barsotti N, Panzeri S, Scattoni ML, Pasqualetti M, Gozzi A (2018) Homozygous loss of autism-risk gene CNTNAP2 results in reduced local and long-range prefrontal functional connectivity. *Cereb Cortex* 28: 1141 – 1153

Mattson MP, Keller JN, Begley JG (1998) Evidence for synaptic apoptosis. *Exp Neurol* 153: 35 – 48

Mirabeau O, Perlas E, Severini C, Audero E, Gascuel O, Possenti R, Birney E, Rosenthal N, Gross C (2007) Identification of novel peptide hormones in the human proteome by hidden Markov model screening. *Genome Res* 17: 320 – 327

Murthy VN, Schikorski T, Stevens CF, Zhu Y (2001) Inactivity produces increases in neurotransmitter release and synapse size. *Neuron* 32: 673 – 682

Nagata S, Suzuki J, Segawa K, Fujii T (2016) Exposure of phosphatidylserine on the cell surface. *Cell Death Differ* 23: 952 – 961

Neniskyte U, Gross CT (2017) Errant gardeners: glial-cell-dependent synaptic pruning and neurodevelopmental disorders. *Nat Rev Neurosci* 18: 658 – 670

O'Leary DD (1987) Remodelling of early axonal projections through the selective elimination of neurons and long axon collaterals. *Ciba Found Symp* 126: 113 – 142

Pagani M, Bertero A, Liska A, Galbusera A, Sabbioni M, Barsotti N, Colenbier N, Marinazzo D, Scattoni ML, Pasqualetti M *et al* (2019) Deletion of autism risk gene Shank3 disrupts prefrontal connectivity. *J Neurosci* 39: 5299 – 5310

Paidassi H, Tacnet-Delorme P, Garlatti V, Darnault C, Ghebrehiwet B, Gaboriaud C, Arlaud GJ, Frachet P (2008) C1q binds phosphatidylserine and likely acts as a multiligand-bridging molecule in apoptotic cell recognition. *J Immunol* 180: 2329 – 2338

Paolicelli RC, Bolasco G, Pagani F, Maggi L, Scianni M, Panzanelli P, Giustetto M, Ferreira TA, Guiducci E, Dumas L *et al* (2011) Synaptic pruning by microglia is necessary for normal brain development. *Science* 333: 1456 – 1458

Personius KE, Balice-Gordon RJ (2001) Loss of correlated motor neuron activity during synaptic competition at developing neuromuscular synapses. *Neuron* 31: 395 – 408

Portera-Cailliau C, Weimer RM, De Paola V, Caroni P, Svoboda K (2005) Diverse modes of axon elaboration in the developing neocortex. *PLoS Biol* 3: e272

Ruggiero L, Connor MP, Chen J, Langen R, Finnemann SC (2012) Diurnal, localized exposure of phosphatidylserine by rod outer segment tips in wild-type but not Itgb5$^{-/-}$ or Mfge8$^{-/-}$ mouse retina. *Proc Natl Acad Sci USA* 109: 8145 – 8148

Sakuragi T, Kanai R, Tsutsumi A, Narita H, Onishi E, Nishino K, Miyazaki T, Baba T, Kosako H, Nakagawa A *et al* (2021) The tertiary structure of the human Xkr8-Basigin complex that scrambles phospholipids at plasma membranes. *Nat Struct Mol Biol* 28: 825 – 834

Sapar ML, Ji H, Wang B, Poe AR, Dubey K, Ren X, Ni JQ, Han C (2018) Phosphatidylserine externalization results from and causes neurite degeneration in Drosophila. *Cell Rep* 24: 2273 – 2286

Schafer DP, Lehrman EK, Kautzman AG, Koyama R, Mardinly AR, Yamasaki R, Ransohoff RM, Greenberg ME, Barres BA, Stevens B (2012) Microglia sculpt postnatal neural circuits in an activity and complement-dependent manner. *Neuron* 74: 691 – 705

Scott-Hewitt N, Perrucci F, Morini R, Erreni M, Mahoney M, Witkowska A, Carey A, Faggiani E, Schuetz LT, Mason S *et al* (2020) Local externalization of phosphatidylserine mediates developmental synaptic pruning by microglia. *EMBO J* 39: e105380

Sekar A, Bialas AR, de Rivera H, Davis A, Hammond TR, Kamitaki N, Tooley K, Presumey J, Baum M, Van Doren V *et al* (2016) Schizophrenia risk from complex variation of complement component 4. *Nature* 530: 177 – 183

Sforazzini F, Schwarz AJ, Galbusera A, Bifone A, Gozzi A (2014) Distributed BOLD and CBV-weighted resting-state networks in the mouse brain. *Neuroimage* 87: 403 – 415

Shacham-Silverberg V, Sar Shalom H, Goldner R, Golan-Vaishenker Y, Gurwicz N, Gokhman I, Yaron A (2018) Phosphatidylserine is a marker for axonal debris engulfment but its exposure can be decoupled from degeneration. *Cell Death Dis* 9: 1116

Stevens B, Allen NJ, Vazquez LE, Howell GR, Christopherson KS, Nouri N, Micheva KD, Mehalow AK, Huberman AD, Stafford B *et al* (2007) The classical complement cascade mediates CNS synapse elimination. *Cell* 131: 1164 – 1178

Stoppini L, Buchs PA, Muller D (1991) A simple method for organotypic cultures of nervous tissue. *J Neurosci Methods* 37: 173 – 182

Suetterlin P, Hurley S, Mohan C, Riegman KLH, Pagani M, Caruso A, Ellegood J, Galbusera A, Crespo-Enriquez I, Michetti C *et al* (2018) Altered neocortical gene expression, brain overgrowth and functional over-connectivity in Chd8 haploinsufficient mice. *Cereb Cortex* 28: 2192 – 2206

Suzuki J, Denning DP, Imanishi E, Horvitz HR, Nagata S (2013a) Xk-related protein 8 and CED-8 promote phosphatidylserine exposure in apoptotic cells. *Science* 341: 403 – 406

Suzuki J, Fujii T, Imao T, Ishihara K, Kuba H, Nagata S (2013b) Calcium-dependent phospholipid scramblase activity of TMEM16 protein family members. *J Biol Chem* 288: 13305 – 13316

Suzuki J, Imanishi E, Nagata S (2014) Exposure of phosphatidylserine by Xk-related protein family members during apoptosis. *J Biol Chem* 289: 30257 – 30267

Suzuki J, Imanishi E, Nagata S (2016) Xkr8 phospholipid scrambling complex in apoptotic phosphatidylserine exposure. *Proc Natl Acad Sci USA* 113: 9509 – 9514

Swanson LW, Hahn JD, Sporns O (2017) Organizing principles for the cerebral cortex network of commissural and association connections. *Proc Natl Acad Sci USA* 114: E9692 – E9701

Tapia JC, Wylie JD, Kasthuri N, Hayworth KJ, Schalek R, Berger DR, Guatimosim C, Seung HS, Lichtman JW (2012) Pervasive synaptic branch removal in the mammalian neuromuscular system at birth. *Neuron* 74: 816 – 829

Timur ZK, Akyildiz Demir S, Marsching C, Sandhoff R, Seyrantepe V (2015) Neuraminidase-1 contributes significantly to the degradation of neuronal B-series gangliosides but not to the bypass of the

© 2023 The Authors

catabolic block in Tay-Sachs mouse models. *Mol Genet Metab Rep* 4: 72 – 82

Vermes I, Haanen C, Steffens-Nakken H, Reutelingsperger C (1995) A novel assay for apoptosis. Flow cytometric detection of phosphatidylserine expression on early apoptotic cells using fluorescein labelled Annexin V. *J Immunol Methods* 184: 39 – 51

Weinhard L, di Bartolomei G, Bolasco G, Machado P, Schieber NL, Neniskyte U, Exiga M, Vadisiute A, Raggioli A, Schertel A *et al* (2018a) Microglia remodel synapses by presynaptic trogocytosis and spine head filopodia induction. *Nat Commun* 9: 1228

Weinhard L, Neniskyte U, Vadisiute A, di Bartolomei G, Aygün N, Riviere L, Zonfrillo F, Dymecki S, Gross C (2018b) Sexual dimorphism of microglia and synapses during mouse postnatal development. *Dev Neurobiol* 78: 618 – 626

Williams DW, Kondo S, Krzyzanowska A, Hiromi Y, Truman JW (2006) Local caspase activity directs engulfment of dendrites during pruning. *Nat Neurosci* 9: 1234 – 1236

Wong FK, Marin O (2019) Developmental cell death in the cerebral cortex. *Annu Rev Cell Dev Biol* 35: 523 – 542

Zalesky A, Fornito A, Bullmore ET (2010) Network-based statistic: identifying differences in brain networks. *Neuroimage* 53: 1197 – 1207

License: This is an open access article under the terms of the Creative Commons Attribution License, which permits use, distribution and reproduction in any medium, provided the original work is properly cited.

 © 2023 The Authors

Expanded View Figures

Figure EV1. Immunolabeling of Xkr8 in *Cx3cr1*::tdTomato microglia.

A, B Immunofluorescence labeling of total Xkr8 (*yellow*) within $tdTomato^+$ microglia (*red*) in the S1 cortex of P0 mouse in mosaic image (A) and enlarged representative cells in layer 4 (B, 1), layer 6 (B, 2) and corpus callosum (B, 3 and 4); *scale bars* 100 µm (A) and 10 µm (B).

▶

© 2023 The Authors

© 2023 The Authors

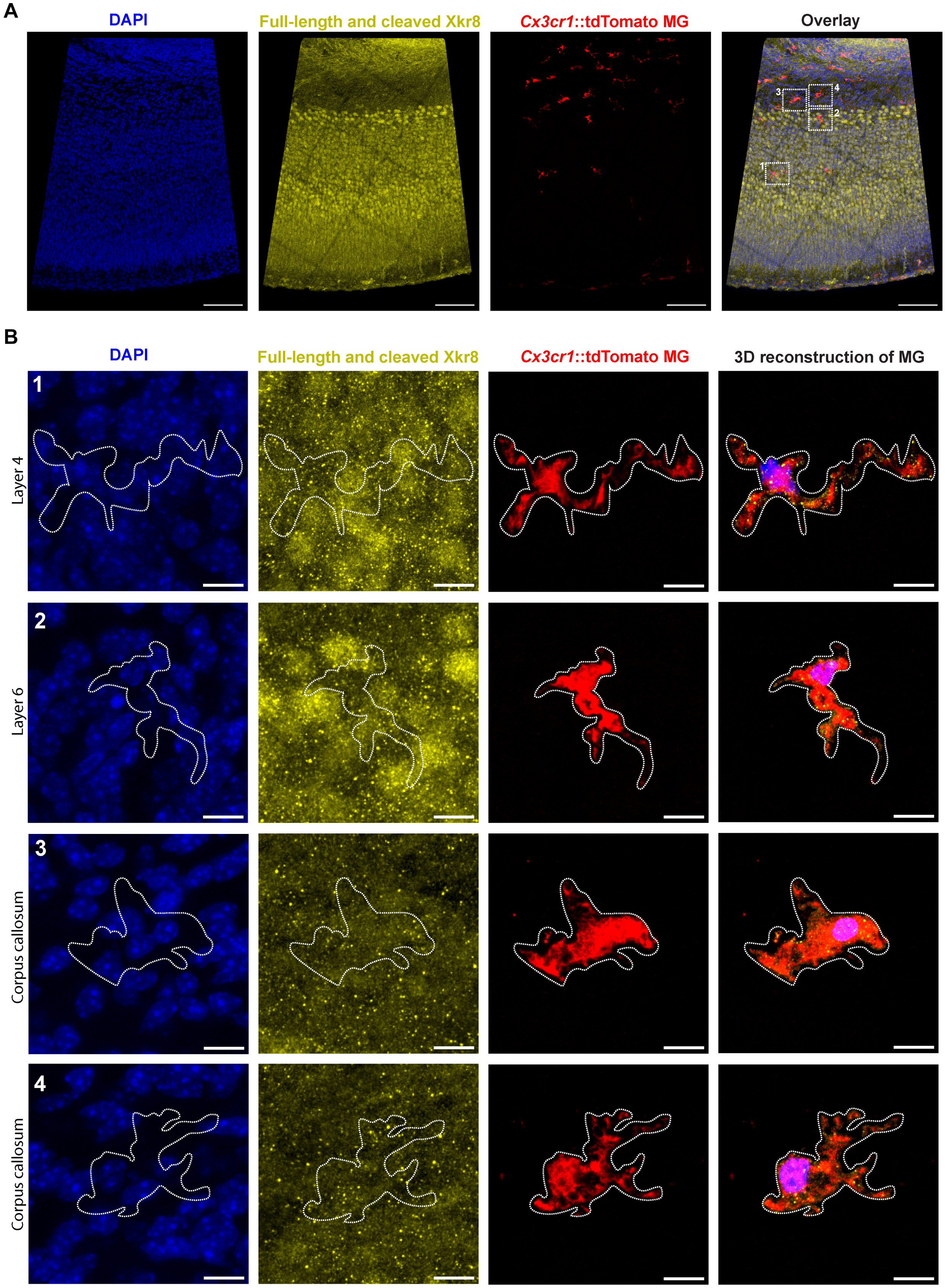

Figure EV1.

 © 2023 The Authors

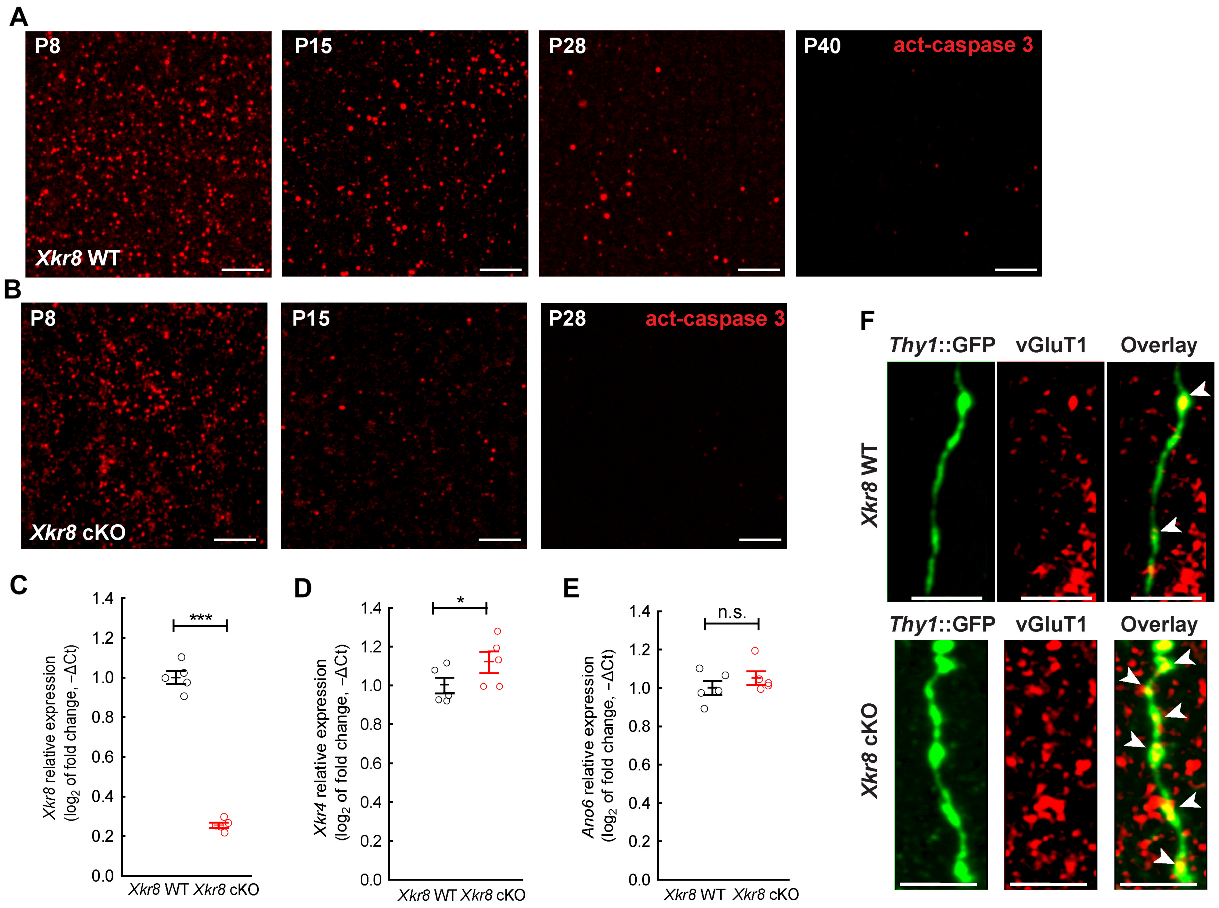

Figure EV2. The characterization of *Xkr8* cKO mouse brain.

A, B Immunofluorescence labeling of active caspase-3 in developing brain of *Xkr8* WT (A) and *Xkr8* cKO (B) mice from P8 to P40; *scale bar* 2 μm. Signal quantification is presented in Fig 2C.

C–E Expression of *Xkr8* (C), *Xkr4* (D) and *Ano6* (E) mRNA in postnatal P0 brain of *Xkr8* WT and *Xkr8* cKO mouse was measured by quantitative RT–PCR, normalized to the expression of *Gadph* at P0 and to the expression of that mRNA in *Xkr8* WT brain. Data were analyzed by one-way ANOVA, each dot represents an individual mouse, $n = 5$ per genotype group; mean ± SEM, $^{*}P < 0.05$, $^{***}P < 0.001$.

F Immunofluorescence labeling of vGluT1 (*red*) in *Thy1*::GFP^{+} axons (*green*) in *Xkr8* WT and *Xkr8* cKO hippocampus. The arrows indicate axonal varicosities; *scale bar* 5 μm.

Source data are available online for this figure.

© 2023 The Authors

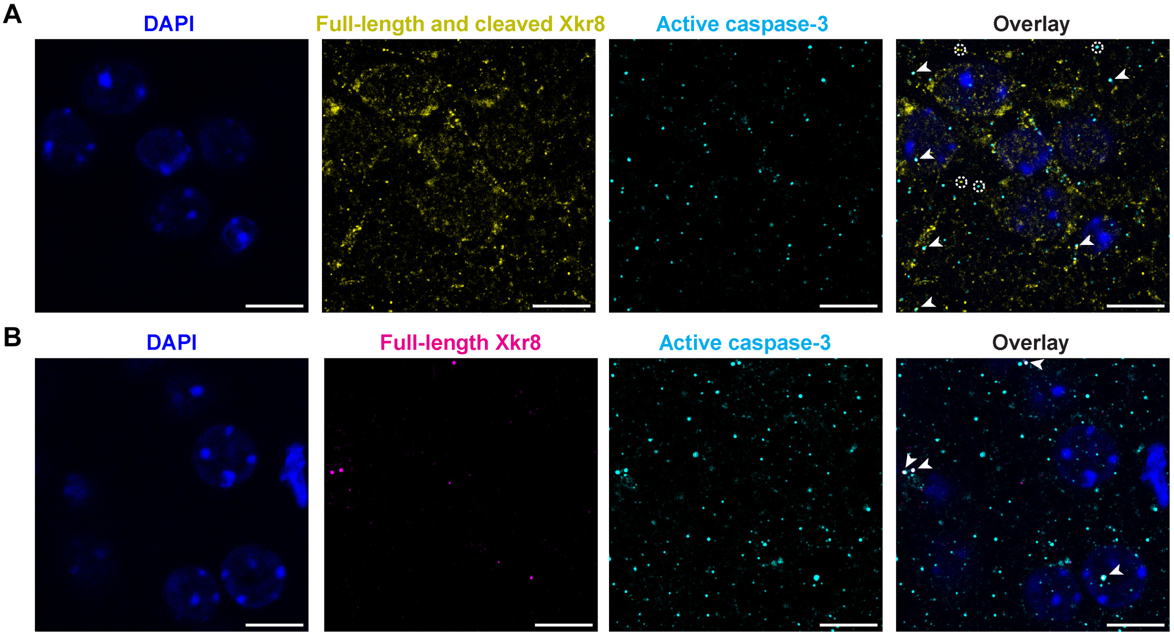

Figure EV3. The collocalization of Xkr8 and active caspase-3 in developing brain.

A, B Immunofluorescence co-labeling of active caspase-3 (*cyan*) and Xkr8 antibodies that recognize either both full-length and cleaved Xkr8 (A, *yellow*) or only full-length Xkr8 (B, *magenta*) in the S1 cortex of P8 mouse. Arrows mark co-localizing particles, dashed circles label particles that do not co-localize; *scale bar* 10 μm.

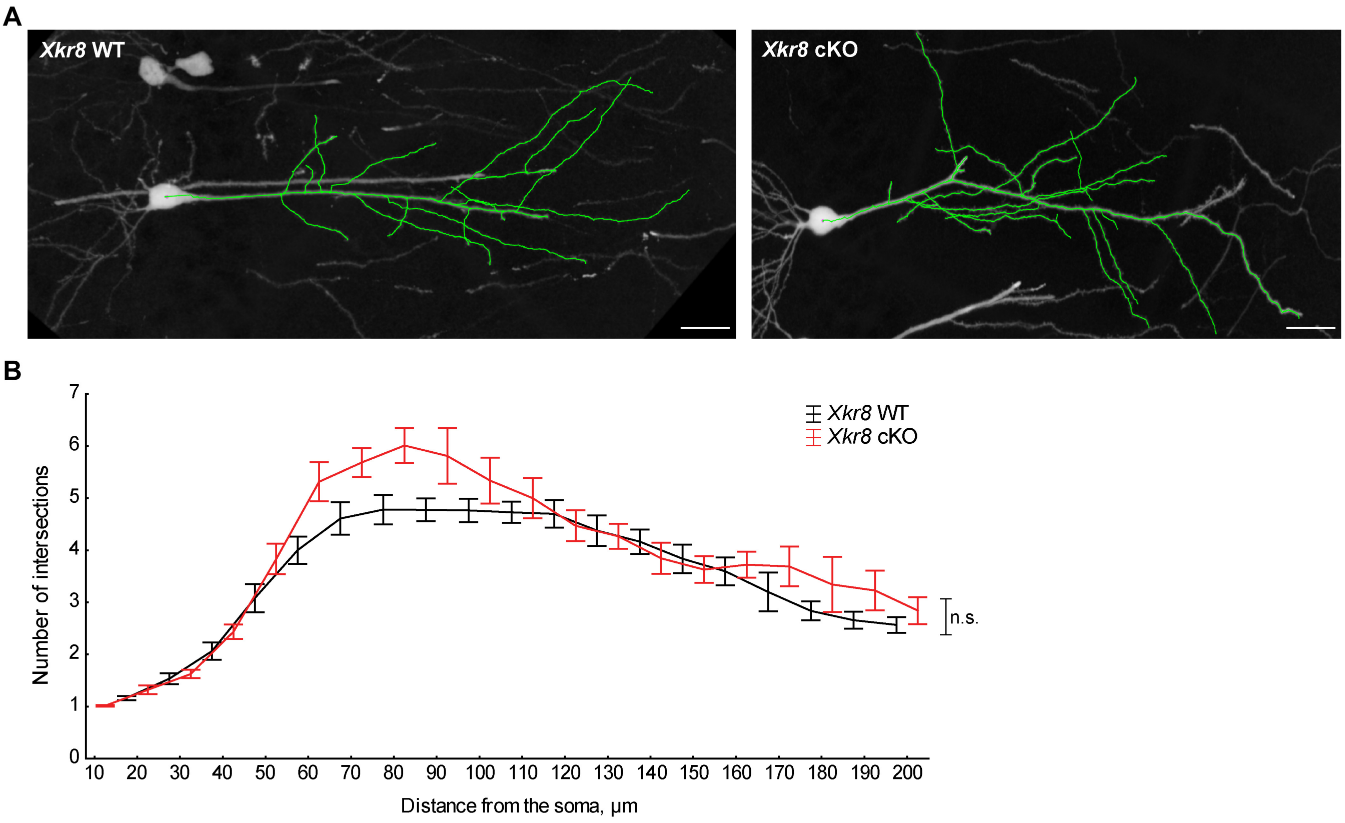

Figure EV4.

© 2023 The Authors

◀ **Figure EV4. *Xkr8* KO did not alter dendritic morphology of pyramidal neurons.**

A Representative dendritic arbors of *Thy1*::GFP^{+} hippocampal CA1 neurons at P28 in *Xkr8* WT and cKO brains; *scale bar* 20 μm.
B Dendritic branching patterns of *Thy1*::GFP^{+} CA1 neurons in *Xkr8* WT and cKO brains were defined by Sholl analysis, which quantifies the number of dendritic branches at predefined distances from the soma (Mann–Whitney test, n = 6 mice per genotype group; mean ± SEM).

Source data are available online for this figure.

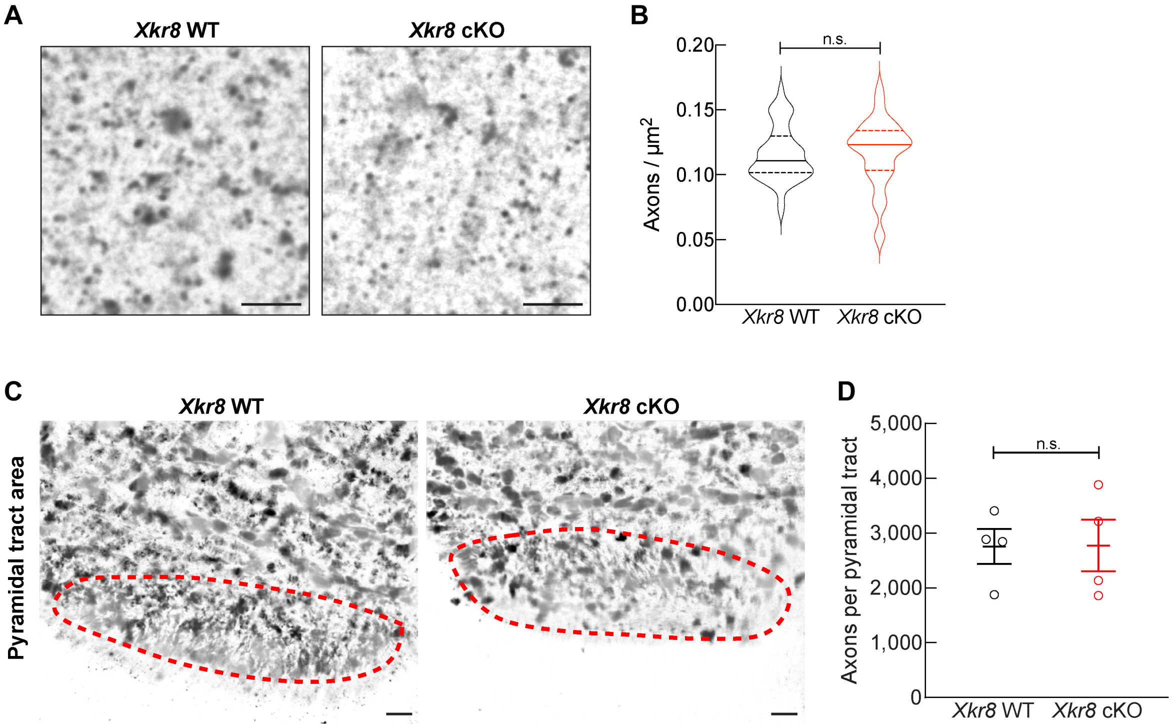

Figure EV5. The density of corticospinal axons in the medulla of P0 *Xkr8* WT and cKO mouse.

A Corticospinal axons of the pyramidal tracts in medulla of P0 *Xkr8* WT and cKO mice were visualized by Palmgren staining in high magnification (60×) to quantify axonal density; *scale bar* 4 μm.
B Corticospinal axon density (Mann–Whitney test, n = 4 mice per genotype, the data are presented as median and quartiles).
C Corticospinal axons of the pyramidal tracts in medulla of P0 *Xkr8* WT and cKO mice were visualized by Palmgren staining in low magnification (20×) to measure pyramidal tract area, delineated by dashed lines; *scale bar* 20 μm.
D Corticospinal axon count per whole pyramidal tract area (two-tailed Student's t-test, n = 4 mice per genotype, the data are presented as mean ± SEM).

Source data are available online for this figure.

© 2023 The Authors

Phospholipid scramblase Xkr8 is required for developmental axon pruning

Urte Neniskyte*, Ugne Kuliesiute, Auguste Vadisiute, Kristina Jevdokimenko, Ludovico Coletta, Senthilkumar Deivasigamani, Daina Pamedytyte, Neringa Daugelaviciene, Daiva Dabkeviciene, Emerald Perlas, Daina Pamedytyte, Ugne Kuliesiute, Aditya Bali, Bernadette Basilico, Alessandro Gozzi, Davide Ragozzino, Cornelius T. Gross*

Appendix

Table of contents

1. Appendix Figure S1 page 2
2. Appendix Figure S2 page 3
3. Appendix Table S1 page 4
4. Appendix Figure S3 page 6
5. Appendix Figure S4 page 7
6. Appendix Figure S5 page 8

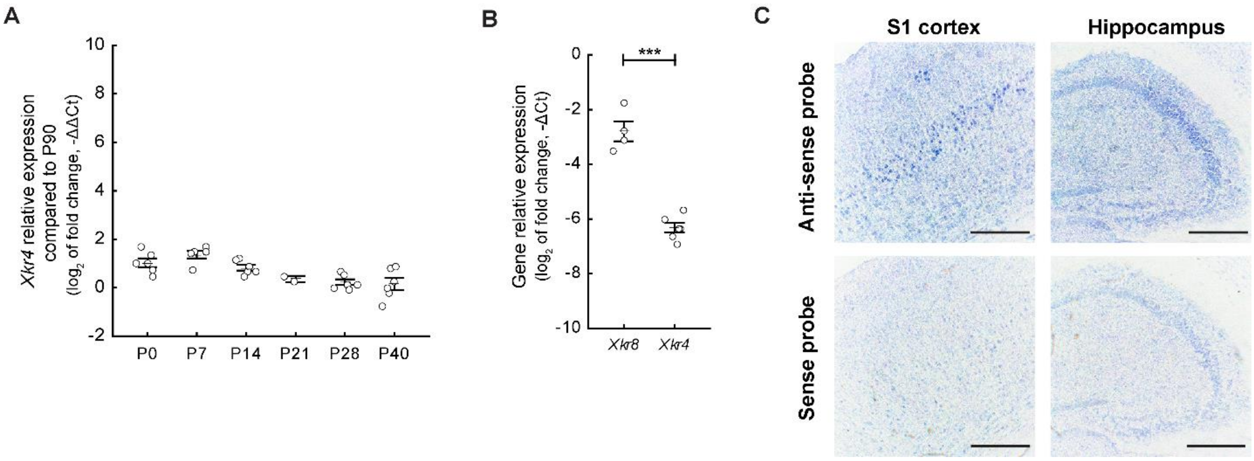

Appendix Figure S1. Expression of *Xkr8* and *Xkr4* in developing brain. A Developmental profile of *Xkr4* mRNA expression in mouse brain from P0 to P40 was measured by quantitative RT-PCR and normalized to the expression of *Gapdh* at a relative time-point and to the expression of the gene of interest at P90. No significant changes in the expression of *Xkr4* were observed during the course of development. (one-way ANOVA, each dot represents an individual mouse, n = 2–6 per age group; mean ± SEM).

B Expression of *Xkr8* and *Xkr4* mRNA in postnatal P0 brain by quantitative RT-PCR normalized to the expression of *Gadph* at P0. Significantly higher expression of *Xkr8* was observed in P0 brains compared to *Xkr4* (one-way ANOVA, each dot represents an individual mouse, n = 2–6 per age group; mean ± SEM, *** $p < 0.001$).

C *Xkr8* mRNA expression in hippocampus and cortex of adult mice visualized by *in situ* hybridization; *scale bar* 200 µm.

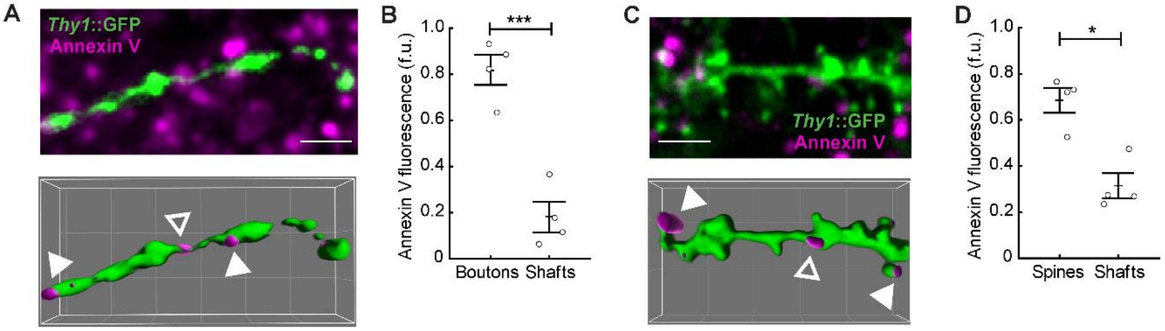

Appendix Figure S2. PtdSer is preferentially exposed on synaptic structures of pyramidal *Thy1*::GFP neurons.

A-D Organotypic hippocampal slices from *Thy1*::GFP (*green*) mice were labelled with PtdSer-binding fluorescently tagged Annexin V (*magenta*) at 16-19 DIV. Fluorescent confocal image *z* stacks were reconstructed on Imaris to obtain 3D surfaces of axons (**A**) and dendrites (**C**); *scale bar* 2 µm, *grid cell* 2 µm. The fluorescence of bound Annexin V within GFP+ was quantified on ImageJ by measuring the integrated density of fluorescent structures within *Thy1*::GFP surface. GFP+ axonal boutons (**B**) and dendritic shafts (**D**) were identified manually by their characteristic structure. Annexin V structures on boutons or spines are labelled with closed arrows, while those on shafts are labelled with open arrows. The data were compared by two-tailed Student's *t*-test, each dot represents OHSC preparation from one mouse, n = 4; mean ± SEM, * $p < 0.05$, *** $p < 0.001$.

Appendix Table S1. Statistical analysis of data presented in Figure 1–3 using sex as an independent variable. (NOTE: Due to small sample sizes, these results are only indicative.)

Two-way/Three-way ANOVA					Mean±SEM; n	
Figure	Effect	Degr. of freedom	F	*p*	Male	Female
1a	SEX	1, 13	0.61	0.45	2.17±0.79; 13	2.79±0.89; 12
	AGE*SEX:	5, 13	0.34	0.88		
	P0				5.87±2.13; 2	8.84±0.49; 2
	P7				2.48±2.58; 3	1.92±1.03; 2
	P14				2.07±1.04; 3	2.61±1.83; 2
	P21				1.20±NA; 1	1.22±0.11; 2
	P28				0.20±0.17; 2	1.72±0.80; 2
	P40				0.58±0.03; 2	0.46±0.05; 2
1g	SEX	1, 26	1.09	0.31	6.3±1.01; 18	8.04±1.51; 18
	AGE*SEX:	2, 26	0.80	0.46		
	P8				8.72±2.51; 6	13.17±3.48; 6
	P15				5.35±1.08; 6	6.50±1.49; 6
	P28				4.95±1.09; 6	4.45±0.87; 6
2c	SEX	1, 26	1.09	0.31	6.3±1.01; 18	8.04±1.51; 18
	AGE*SEX:	2, 26	0.80	0.46		
	P8				8.72±2.51; 6	13.17±3.48; 6
	P15				5.35±1.08; 6	6.50±1.49; 6
	P28				4.95±1.09; 6	4.45±0.87; 6
	GENE*SEX:	1, 26	0.20	0.66		
	WT				6.93±1.40; 9	9.34±1.22; 9
	cKO				5.76±1.51; 9	6.74±2.79; 9
3b	SEX	1, 26	11.59	**0.002**	**0.13±0.006; 18**	**0.11±0.004; 18**
	AGE*SEX:	2, 26	1.82	0.18		
	P8				0.15±0.006; 6	0.12±0.007; 6
	P15				0.12±0.008; 6	0.11±0.005; 6
	P28				0.13±0.011; 6	0.10±0.007; 6
	GENE*SEX:	1, 26	0.01	0.93		
	WT				0.12±0.009; 9	0.10±0.006; 9
	cKO				0.14±0.007; 9	0.12±0.004; 9
3c	SEX	1, 26	5.08	**0.03**	**2.96±0.12; 18**	**2.66±0.09; 18**
	AGE*SEX:	2, 26	0.76	0.48		
	P8				3.39±0.06; 6	2.91±0.11; 6
	P15				2.72±0.21; 6	2.37±0.15; 6
	P28				2.78±0.23; 6	2.69±0.11; 6
	GENE*SEX:	1, 26	0.49	0.49		
	Wt				2.93±0.17; 9	2.72±0.16; 9
	cKO				2.99±0.18; 9	2.60±0.07; 9

Continued on the next page

Continued from the last page

3e	SEX<br>AGE*SEX:<br>P8<br>P15<br>P28<br>GENE*SEX:<br>WT<br>cKO	1, 26<br>2, 26<br><br><br><br>1, 26	2.41<br>3.23<br><br><br><br>0.11	0.13<br>0.06<br><br><br><br>0.78	13.29±1.63; 18<br><br>3.97±0.51; 6<br>17.07±0.36; 6<br>18.83±0.57; 6<br><br>13.04±2.38; 9<br>13.54±2.36; 9	12.69±1.83; 18<br><br>2.25±0.44; 6<br>16.34±0.36; 6<br>19.49±0.47; 6<br><br>12.56±2.50; 9<br>12.83±2.83; 9
3f	SEX<br>GENE*SEX:<br>WT<br>cKO	1, 8<br>1, 8	0.72<br>0.01	0.42<br>0.93	1059±59; 6<br><br>1002±70; 3<br>1116±96; 3	980±68; 6<br><br>932±58; 3<br>1029±131; 3
3j	SEX<br>AGE*SEX:<br>P8<br>P28<br>GENE*SEX:<br>WT<br>cKO	1, 17<br>1, 17<br><br><br>1, 17	0.58<br>4.75<br><br><br>2.53	0.46<br>**0.04**<br><br><br>0.13	48.1±6.4; 12<br><br>**33.1±9.6; 6**<br>**63.0±1.4; 6**<br><br>39.7±11.6; 6<br>56.4±4.5; 6	52.3±6.6; 12<br><br>**49.5±12.1; 6**<br>**55.1±6.6; 6**<br><br>35.1±8.6; 6<br>69.4±1.7; 6
3k	SEX<br>AGE*SEX:<br>P8<br>P28<br>GENE*SEX:<br>WT<br>cKO	1, 17<br>1, 17<br><br><br>1, 17	0.04<br>2.46<br><br><br>2.86	0.84<br>0.14<br><br><br>0.11	23.9±4.1; 12<br><br>15.7±3.3; 6<br>32.1±6.3; 6<br><br>27.5±8.1;6<br>20.3±2.5; 6	24.7±2.5; 12<br><br>22.7±5.0; 6<br>26.8±1.4; 6<br><br>21.7±4.6; 6<br>27.8±1.7; 6
3l	SEX<br>AGE*SEX:<br>P8<br>P28<br>GENE*SEX:<br>WT<br>cKO	1, 17<br>1, 17<br><br><br>1, 17	3.16<br>0.48<br><br><br>6.86	0.09<br>0.50<br><br><br>**0.02**	2.52±0.44;12<br><br>2.32±0.66; 6<br>2.72±0.65; 6<br><br>**1.25±0.16; 6**<br>**3.78±0.45; 6**	2.02±0.19; 6<br><br>2.01±0.31; 6<br>2.03±0.27; 6<br><br>**1.49±0.16; 6**<br>**2.56±0.18; 6**

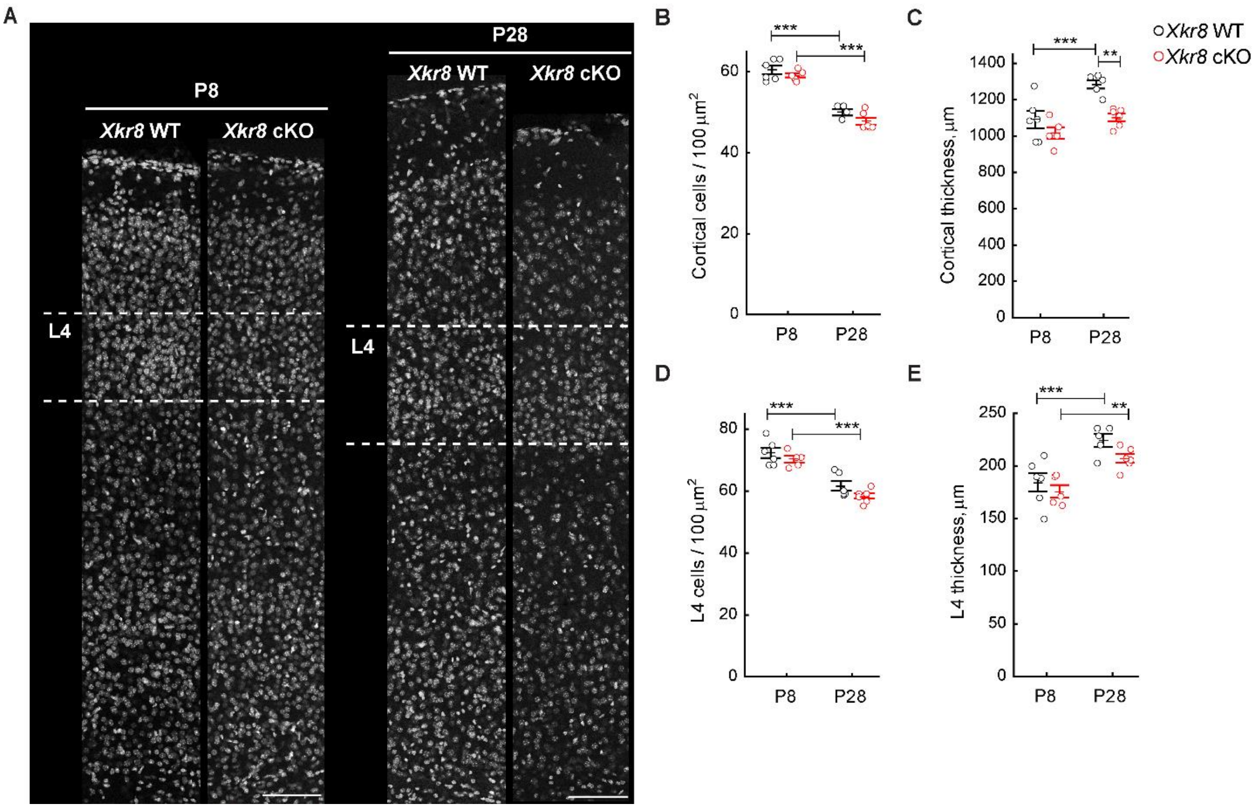

Appendix Figure S3. No decrease in developmental cell loss in *Xkr8* knockout.
A Cortex of *Xkr8* WT and cKO mice was labelled with DAPI to identify nuclei; *scale bar* 100 µm.
B-E (B, D) Quantification of cortical and layer 4 (L4) cell density revealed no difference between *Xkr8* WT and cKO animals. (C, E) Cortical thickness and layer 4 (L4) thickness did not increase in *Xkr8* cKO animals (nested design and mixed model ANOVA, each dot represents an individual mouse, n = 4–6 per age and genotype group; mean ± SEM, ** $p < 0.01$, *** $p < 0.001$).

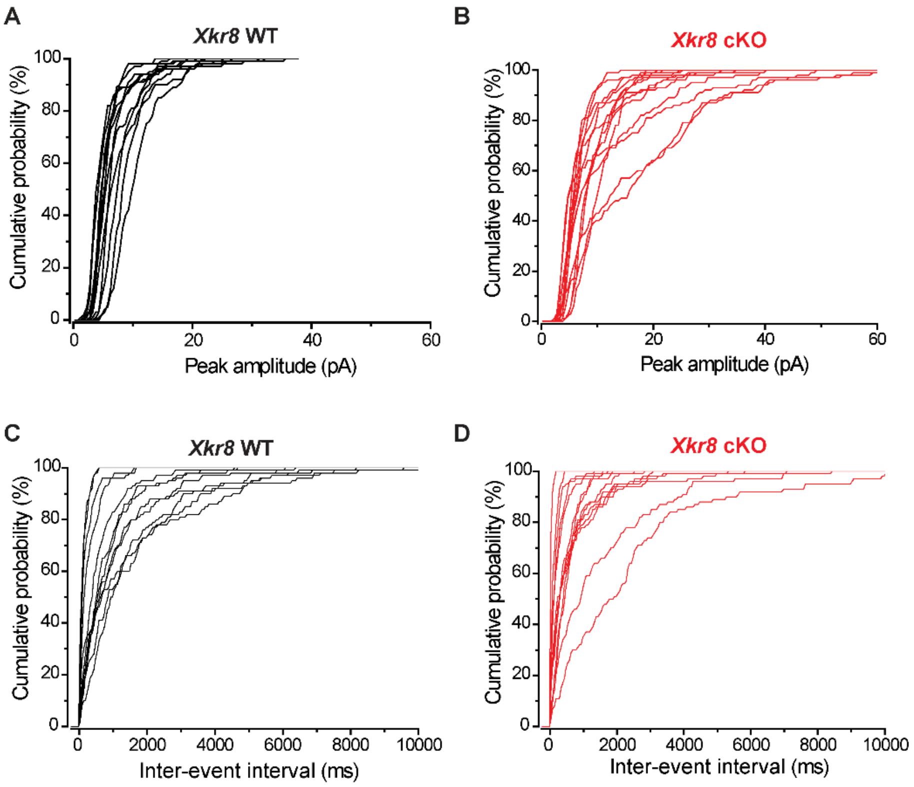

Appendix Figure S4. Synaptic activity of individual neurons in *Xkr8* cKO and WT hippocampus.

A-D (A, B) Individual traces of sEPSC peak amplitudes of *Xkr8* WT and cKO neurons. (C, D) Individual traces of sEPSC inter-event intervals of *Xkr8* WT and cKO neurons. A subset of *Xkr8* cKO neurons with particularly high synaptic activity (B, D) compared to *Xkr8* WT neurons (A, C) was observed.

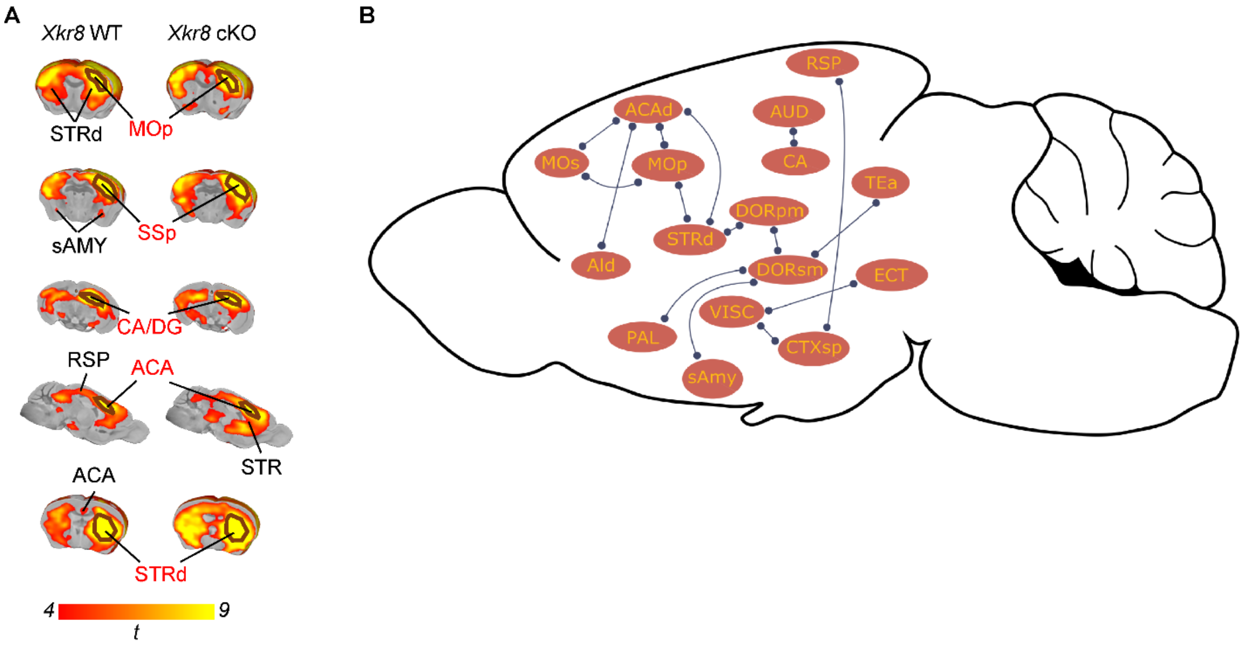

Appendix Figure S5. Seed-based correlation maps of functional connectivity in *Xkr8* WT and cKO mice.

A Seed-based correlation maps in control and *Xkr8* cKO mice (seed area delineated in black) with red/yellow heat-maps indicating regions exhibiting significant rsfMRI connectivity with the seed volume. The corresponding between-group voxel-wise differences have been reported in **Figure 7**.

B Inter-group comparison of intra-hemispheric rsfMRI connectivity in regionally-parcellated brains using network-based statistics (NBS) showed regions exhibiting increased intra-hemispheric connectivity ($t > 2.1$, FWE corrected at $p < 0.05$). Only pairs of regions characterized by significantly increased intra-hemispheric connectivity independently in both hemispheres were retained for display.

Data information: ACA: anterior cingulate cortex, ACAd: anterior cingulate cortex, dorsal, AId: agranular insular area, dorsal, sAMY: striatum-like amygdalar nuclei, AUD: auditory areas, CA: Ammon's horn, CTXsp: cortical subplate, DG: dentate gyrus, DORpm: poly-modal association cortex-related region, DORsm: sensory-motor cortex-related region, ECT: ectorhinal area, MOp: primary motor area, MOs: secondary motor area, PAL: pallidum, RSP: retrosplenial area, SSp: primary somatosensory area, STR: striatum, STRd: striatum dorsal region, SUB: subiculum, TEa: temporal association areas, VIS: visual areas, VISC: visceral area.
