# Supplementary material for: Phospholipid scramblase Xkr8 is required for developmental axon pruning via phosphatidylserine exposure
Source: EMBO J. 2023 May 22;42(14):e111790. doi: 10.15252/embj.2022111790 (PMC10350823; doi:10.15252/embj.2022111790)
Supplement: Supplementary file 12 [file EMBJ-42-e111790-s007.pdf]

THE EMBO JOURNAL

Phospholipid scramblase Xkr8 is required for developmental axon pruning via phosphatidylserine exposure

Urte Neniskyte, Ugne Kuliesiute, Auguste Vadisiute, Kristina Jevdokimenko, Ludovico Coletta, Senthilkumar Deivasigamani, Daina Pamedytyte, Neringa Daugelaviciene, Daiva Dabkeviciene, Emerald Perlas, Aditya Bali, Bernadette Basilico, Alessandro Gozzi, Davide Ragozzino, and Cornelius Gross
DOI: 10.15252/embj.2022111790

Corresponding author(s): Cornelius Gross (gross@embl.it) , Urte Neniskyte (urte.neniskyte@gmc.vu.lt)

Review Timeline:

Submission Date:	31st May 22
Editorial Correspondence	28th June 22
Editorial Decision:	18th Aug 22
Revision Received:	30th Jan 23
Editorial Decision:	17th Mar 23
Revision Received:	18th Apr 23
Accepted:	25th Apr 23

Editor: Karin Dumstrei

Transaction Report:

(Note: With the exception of the correction of typographical or spelling errors that could be a source of ambiguity, letters and reports are not edited. Depending on transfer agreements, referee reports obtained elsewhere may or may not be included in this compilation. Referee reports are anonymous unless the Referee chooses to sign their reports.)

Dear Cornelius,

Thank you for submitting your manuscript to The EMBO Journal. Your study has now been seen by two referees and I have provided the comments below.

As you can see, the referees find the analysis interesting but also find that much further work is needed for consideration here. As it is not clear if you can address the raised concerns, I find it most productive to ask you to provide a point-by-point response upfront and based upon this I would then take the decision on the manuscript.

I am also available to discuss your response via a video call if that is helpful.

With best wishes

Karin

Karin Dumstrei, PhD
Senior Editor
The EMBO Journal

Dear Cornelius and Urte,

Thank you for sending me your response to the concerns raised and the data you can include to address the raised concerns.

I have now run your response by referee #2 and heard back from the referee.

The referee appreciates the proposed changes and is open to looking at a revised version.

Given this, I would therefore like to invite you to submit a revised version.

When preparing your letter of response to the referees' comments, please bear in mind that this will form part of the Review Process File, and will therefore be available online to the community. For more details on our Transparent Editorial Process, please visit our website: https://www.embopress.org/page/journal/14602075/authorguide#transparentprocess

Thank you for the opportunity to consider your work for publication. I look forward to your revision.

Yours sincerely,

Karin

Karin Dumstrei, PhD
Senior Editor
The EMBO Journal

Instructions for preparing your revised manuscript:

Please see attached guide with helpful tips on how to prepare the revised version.

Guide For Authors: https://www.embopress.org/page/journal/14602075/authorguide

We realize that it is difficult to revise to a specific deadline. In the interest of protecting the conceptual advance provided by the work, we recommend a revision within 3 months (16th Nov 2022).

As a matter of policy, competing manuscripts published during this period will not negatively impact on our assessment of the conceptual advance presented by your study. However, we request that you contact the editor as soon as possible upon publication of any related work, to discuss how to proceed.

If you require more time to complete the revisions let me know as as I can grant an extension.

Referee #1:

In this review, Neniskyte and colleagues aim to further elucidate the molecular underpinnings of developmental circuit formation. They conclude that Xkr8 is the phospholipid scrambalase expressed by neurons that allows phosphatidylserine to be present on the extracellular surface and that loss of this scrambalase results in exuberant axon tracts in vivo and connectivity electrophysiologically. They also link Xkr8 cleavage to caspase activity, suggesting an apoptosis-like program acting at the axon (axon-death). They thus conclude that neuron-based Xkr8 is the requisite enzyme that directs axonal pruning. Overall, this is a compelling study with fluorescent micrographs that are textbook in their beauty. While this is an area of great investigation, there is much to be learned and this study provides a welcome advance. There is one (and a half) major and few minor concerns that if addressed would greatly improve the impact of the study and better support its conclusions.

The authors (rightly) invoke the idea of axon pruning in light of known non-cell autonomous mechanisms by which phagocytic cells sculpt synapses and (presumably) axons tagged by exposed phosphatidylserine. It is also possible, as can occur at the developing Drosophila neuromuscular junction, that there is some degree of autonomous dying-back. An alternative explanation - given the analysis window, and the known role that external phosphatidylserine can play in adhesion - is that loss of Xkr8 leads

not to decreased pruning but over-exuberance of axon formation in the first place. The authors should either evaluate an earlier time period (prenatally, when perhaps there are equal axons density formed but before axon pruning), demonstrate that Xkr8 loss does not lead to a change in axon formation (perhaps in vitro?), decreases axon engulfment, or some other evaluation that better supports the conclusion that this is an axon pruning phenotyping rather than a neurite outgrowth one. An alternative strategy is to reframe the study about how externalized phosphatidylserine controls axon number, regardless of the underlying mechanism.
The half concern is the specificity of the Xkr8 knockdown. Some cell type specificity with double immunostaining or in situ is valuable in light of Emx1 being a constitutive Cre that can also target glia developmentally... perhaps showing the normal cell-specific distribution of Xkr8 would exonerate a glial phosphatidylserine.

Minor concerns:
Link between caspase 3 and XKR8 is not wholly clear as they are not necessarily shown to be co-distributed. (consider double labeling)

Which cells are expressing XKR8? Appears to be neurons but is that all?

Figure 1 - beautiful immunohistochemistry - orient the readers to the layers by notating the surface

XKR8 "upregulated" during development but given lack of prenatal time points, not sure if a peak; more accurate would be "downregulated" during development.

How intriguing is that population of adult cortical neurons with full length Xkr8-expression? I wonder what they are doing...

Referee #2:

In this manuscript the authors demonstrate that the phospholipid scramblase Xkr8, which is developmentally upregulated in the early postnatal period, is required for phosphatidylserine exposure in hippocampus. They show that lack of Xkr8 results in excess excitatory terminals, elevated density of cortico-cortical projections and global brain hyperconnectivity. They conclude that Xkr8 is selectively involved in promoting axonal pruning, which offers a powerful tool for investigations aimed at identifying the mechanisms discriminating the axons destined to be eliminated from those that survive.

Although the identification of Xkr8 as required for the neuronal externalization of phosphatidylserine is relevant for a better understanding of brain development and circuit formation, its involvement in the process of axon elimination, proposed by the authors, is not convincingly demonstrated. No evidence is provided for the glial engulfment of synaptic or axonal material in Xkr8 WT versus KO mice, and the neuronal structures (synapses, axons, other) exposing phosphatidylserine are not identified. Also, whether differences in connectivity and synapse activity in KO versus WT mice are the consequences of altered synaptic -or axonal- pruning is not demonstrated. Finally, no characterization of the Xkr8 KO mice is provided.

Specific points:

Fig. 1D: I am unable to see the full-length staining at any time points. If, as the authors state, "full-length, uncleaved Xkr8 was depleted in the early postnatal brain" it would be nice to have a detectable prenatal staining. Differently, one can only state that the full length plus cleaved Xkr8 (green staining) is highest in early postnatal period. There is no evidence that the cleavage is higher in the early postnatal period.

Fig. 1H shows some annexin spots, but it is not clear what they do represent. Are these axons? Are they synapses? What is PtdSer exposure normalized to in fig.1I? DAPI staining should be performed, together with dendritic/axonal and synaptic staining. The authors should also demonstrate that PtdSer exposure reduction occurs only in hippocampal pyramidal neurons, where Xkr8 is specifically downregulated.

Fig. 1: a characterization of the Xkr8 KO mouse should be provided. Is Xkr4 normally expressed? Are TMEM proteins normally expressed?

Fig. 2A: the boutons should be labeled with both pre and postsynaptic markers to confirm that they are in fact synaptic terminals.

Fig. 2B: the fact that the reduction of bouton size is the consequence of a reduction in the efficiency of synaptic trogocytosis is speculative and would require a direct demonstration. Also, the authors should underline and discuss the evidence that the reduction in the bouton size is detectable only at P28, while Xkr8 is expressed at P0 and drastically downregulated already at P7.

Fig. 2D: the authors should analyze the dendritic spines by differentiating them in mature versus immature (mushroom versus

filopodia). In the absence of these analyses and without a staining for PSD95, which labels functionally mature spines, it is almost impossible to state that there is "relatively undisturbed maturation of excitatory synapses in Xkr8 cKO mice".

Fig. 2G and J show that Xkr8 cKO mice display increased density of presynaptic puncta. The authors should quantify the engulfed synaptic material in glial cells to demonstrate whether this increase results from defective synapse elimination. A quantification of the postsynaptic marker PSD-95 would also be useful to discriminate between presynaptic and postsynaptic involvement.

The sentence "In the absence of a change in bouton density along individual axons, such an increase in the absolute density of excitatory terminals points to a deficiency in the elimination of entire axon arbors, rather than the removal of individual boutons" is rather speculative, given that the boutons in 2A are not demonstrated to be true synapses. In the absence of a clear identification of the structures which expose PtdSer (see fig. 1H), and without analyzing the nature of the engulfed material in glial cells, statements concerning whether isolated synapses or entire axons are engulfed are not supported by data.

Fig. 2K: the authors should explain why a clear effect is also detectable in thalamic vGlut2 positive boutons, which express Xkr8.

Fig. 4: The authors analyze spontaneous and evoked activity in CA1 pyramidal neurons in hippocampal slices from Xkr8 cKO and wild-type control mice at P40 and find significant increase in the amplitude of spontaneous, but not evoked, events. Since spontaneous activity reflects the general circuitry organization, including inhibitory afferents, analyses of the frequency and amplitude of miniature events would be crucial to better disentangle synaptic events. The analysis should be performed also at early postnatal stages, when Xkr8 is highly expressed.

The reasons put forward to explain the lack of differences in evoked events (page 10, lines 8-13) should be more clearly expressed.

The discussion is very speculative.

Minor points
Fig. 1A display a quite high data variability. Did the authors separately analyze males and females?

Fig. 3D: a higher number of observations would be required to unequivocally exclude the lack of effect in S2.

The authors could calculate whether lack of Xkr8 affects synaptic multiplicity, i.e. the density of axon terminals onto a given postsynaptic neuron.

Point-by-point Rebuttal, Neniskyte et al.

Referee #1

In this review, Neniskyte and colleagues aim to further elucidate the molecular underpinnings of developmental circuit formation. They conclude that Xkr8 is the phospholipid scrambalase expressed by neurons that allows phosphatidylserine to be present on the extracellular surface and that loss of this scrambalase results in exuberant axon tracts in vivo and connectivity electrophysiologically. They also link Xkr8 cleavage to caspase activity, suggesting an apoptosis-like program acting at the axon (axon-death). They thus conclude that neuron-based Xkr8 is the requisite enzyme that directs axonal pruning. Overall, this is a compelling study with fluorescent micrographs that are textbook in their beauty. While this is an area of great investigation, there is much to be learned and this study provides a welcome advance. There is one (and a half) major and few minor concerns that if addressed would greatly improve the impact of the study and better support its conclusions.

The authors (rightly) invoke the idea of axon pruning in light of known non-cell autonomous mechanisms by which phagocytic cells sculpt synapses and (presumably) axons tagged by exposed phosphatidylserine. It is also possible, as can occur at the developing Drosophila neuromuscular junction, that there is some degree of autonomous dying-back. An alternative explanation - given the analysis window, and the known role that external phosphatidylserine can play in adhesion - is that loss of Xkr8 leads not to decreased pruning but over-exuberance of axon formation in the first place. The authors should either evaluate an earlier time period (prenatally, when perhaps there are equal axons density formed but before axon pruning), demonstrate that Xkr8 loss does not lead to a change in axon formation (perhaps in vitro?), decreases axon engulfment, or some other evaluation that better supports the conclusion that this is an axon pruning phenotyping rather than a neurite outgrowth one. An alternative strategy is to reframe the study about how externalized phosphatidylserine controls axon number, regardless of the underlying mechanism.

Authors' response: *We agree with the reviewer that a role for Xkr8 in axonal outgrowth is possible, therefore we have now evaluated axon density in the medulla of* Xkr8 *cKO and control wild-type mice* in vivo *at P0, P8 and P28 and found that there was no difference in axonal density at P0 and increased axonal density at P8 that was corrected by P28, in line with our data presented in* ***Figure 3****.*

The half concern is the specificity of the Xkr8 knockdown. Some cell type specificity with double immunostaining or in situ is valuable in light of Emx1 being a constitutive Cre that can also target glia developmentally... perhaps showing the normal cell-specific distribution of Xkr8 would exonerate a glial phosphatidylserine.

Authors' response: *The reviewer is correct to note that the* Emx1*::Cre driver is active in both cortical neurons and astrocytes/oligodendrocytes due to its expression in radial glia progenitors and that deletion of Xkr8 from these glial cell-types could contribute to adult phenotypes in our mice. However, astrocytes and oligodendrocytes emerge at later stages of cortical development than neurons (Qian et al., 2000, 10.1016/s0896-6273(00)00086-6; Shen*

et al., 2006, 10.1038/nn1694) – after the perinatal pruning phenomenon we examine – making it unlikely that an absence of Xkr8 in glia contributes to the phenotypes we observed, at least those related to perinatal neuronal development. Moreover, the expression of Xkr8 as visualized by in situ *labelling of* Xkr8 *mRNA (**Figure EV1c**) appears restricted to principal neurons in hippocampus and cortex, arguing against a prominent role for Xkr8 in astrocytes/oligodendrocytes. We have explicitly discussed these points in the revised manuscript. We have also evaluated Xkr8 collocalization with microglial cells in wild-type mice and found very low staining of Xkr8 within microglia (**Figure EV2**).*

Minor concerns:
Link between caspase 3 and XKR8 is not wholly clear as they are not necessarily shown to be co-distributed. (consider double labeling)

***Authors' response:** We have performed IHC co-labelling to demonstrate the distribution of Xkr8 and caspase-3 and found that active caspase-3 co-localizes with both Xkr8, in particular with full-length isoform in the cortex of P8 mouse (**Figure EV4**).*

Which cells are expressing XKR8? Appears to be neurons but is that all?

***Authors' response:** The expression of Xkr8 as visualized by* in situ *labelling of* Xkr8 *mRNA (**Figure EV1c**) appears restricted to principal neurons in hippocampus and cortex. At P0 and P8 Xkr8 immunofluorescence is also predominant in the granular layer of neuronal bodies (**Figure 1e**). To support our observation of predominant Xkr8 expression in neurons, we have evaluated Xkr8 collocalization with microglia and found very low staining of Xkr8 within microglia (**Figure EV2**).*

Figure 1 - beautiful immunohistochemistry - orient the readers to the layers by notating the surface

***Authors' response:** We believe the reviewer meant **Figure 1c, d**. We have indicated the surface of the tissue as suggested.*

XKR8 "upregulated" during development but given lack of prenatal time points, not sure if a peak; more accurate would be "downregulated" during development.

***Authors' response:** We agree with the reviewer that it may be misleading to refer to the expression changes as 'upregulated' and have revised the text to reflect the postnatal dynamics of Xkr8 more precisely.*

How intriguing is that population of adult cortical neurons with full length Xkr8-expression? I wonder what they are doing...

***Authors' response:** We agree that our data point to a possible role of Xkr8 in adulthood where we speculate that Xkr8-dependent PS exposure could be required for neuronal plasticity or apoptosis; however, addressing such a function is beyond the scope of this manuscript.*

Referee #2

In this manuscript the authors demonstrate that the phospholipid scramblase Xkr8, which is developmentally upregulated in the early postnatal period, is required for phosphatidylserine exposure in hippocampus. They show that lack of Xkr8 results in excess excitatory terminals, elevated density of cortico-cortical projections and global brain hyperconnectivity. They conclude that Xkr8 is selectively involved in promoting axonal pruning, which offers a powerful tool for investigations aimed at identifying the mechanisms discriminating the axons destined to be eliminated from those that survive.

Although the identification of Xkr8 as required for the neuronal externalization of phosphatidylserine is relevant for a better understanding of brain development and circuit formation, its involvement in the process of axon elimination, proposed by the authors, is not convincingly demonstrated. No evidence is provided for the glial engulfment of synaptic or axonal material in Xkr8 WT versus KO mice, and the neuronal structures (synapses, axons, other) exposing phosphatidylserine are not identified. Also, whether differences in connectivity and synapse activity in KO versus WT mice are the consequences of altered synaptic -or axonal-pruning is not demonstrated. Finally, no characterization of the Xkr8 KO mice is provided.

Authors' response: *We are pleased that the reviewer acknowledges the importance of the identification of Xkr8 as a critical mediator of PS exposure during neuronal development. We note that in this study we did not aim to identify the cellular phagocyte responsible for axon engulfment mainly because the live imaging necessary to definitively identify such phagocytic clearance is challenging during the perinatal period in mice. However, previous imaging studies in fixed tissue have found evidence for phagocytic microglia in developing white matter tracts during the perinatal period making them a prime phagocyte candidate. We have performed colocalization of pan-axonal SMI312 marker, vGluT1 marker for pre-synaptic material and PSD95 marker for post-synaptic material with the microglia marker Iba1 to confirm such phagocytic activity in our mice during the early postnatal period and determined that there is significant reduction of SMI312 and vGluT1, but not PSD95 collocalization with microglia in* Xkr8 *cKO mice, suggesting that Xkr8 is required for the uptake of axonal rather than dendritic material (**Figure 4**).*

*Concerning the question about PtdSer localization, we have added data demonstrating that PtdSer (labelled by Annexin V in live organotypic slices) is preferentially exposed on synaptic structures (**Figure EV5**). We agree with the reviewer that it is difficult to rule out that the adult phenotypes we observed in* Xkr8 *cKO mice are not the result of a role for Xkr8 in physiological processes beyond axonal elimination, including a potential function in adulthood, as discussed above. However, it is hard to think of an experimental manipulation that would definitively address this issue.* Xkr8 *cKO mice with conditionally restored Xkr8 function adulthood could be used to rule out an adult contribution to these phenotypes, but combining cell-type and temporally specific gene manipulation is challenging in mice. Alternatively, further evidence for a role of Xkr8-dependent axonal elimination could come from mice in which the PS receptors were conditionally manipulated. However, the PS receptor involved in axonal maturation has not been identified and its identification and functional manipulation would arguably constitute a major effort beyond the scope of the present study. We argue, nevertheless, that our study constitutes an important advance in the field by identifying the machinery responsible for PS exposure during neuronal development and causally linking this*

exposure to axonal maturation phenotypes during development and for the first time identifying deficits in circuitry function and interhemispheric functional connectivity that might depend on such developmental phenomena.

As regards the reviewer's final question, we agree that the manuscript would benefit from the description of Xkr8 *cKO mice. As requested below we have added data on the expression of other scramblases in Xkr8 cKO mice (**Figure EV3c-e**). We have also added a concise description of Xkr8 cKO mice phenotype, which does not have any gross developmental or behavioral abnormalities. We feel that a complete characterization of the Xkr8 cKO mice at, say, the behavioral level, would not have significantly strengthened the manuscript which was focused on the cellular mechanisms responsible for axonal growth and because we did not have strong hypotheses about what sort of behavioral changes might arise as a result of altered axonal connectivity and not having any* a priori *hypothesis, for example, deriving from patients lacking Xkr8.*

Specific points:

Fig. 1D: I am unable to see the full-length staining at any time points. If, as the authors state, "full-length, uncleaved Xkr8 was depleted in the early postnatal brain" it would be nice to have a detectable prenatal staining. Differently, one can only state that the full length plus cleaved Xkr8 (green staining) is highest in early postnatal period. There is no evidence that the cleavage is higher in the early postnatal period.

Authors' response: *We apologize for the faint signal quality in* ***Figure 1d, f****. We have increased the contrast to allow better assessment of expression pattern and changes across ages. We agree that the terms 'upregulation' and 'peak' could be misleading and have adjusted the text to more closely reflect the finding that* Xkr8 *mRNA and protein expression is high in the perinatal period and decreases across development.*

Fig. 1H shows some annexin spots, but it is not clear what they do represent. Are these axons? Are they synapses? What is PtdSer exposure normalized to in fig.1I? DAPI staining should be performed, together with dendritic/axonal and synaptic staining. The authors should also demonstrate that PtdSer exposure reduction occurs only in hippocampal pyramidal neurons, where Xkr8 is specifically downregulated.

Authors' response: *Annexin V staining across samples in current* ***Figure 2a*** *was quantified using identical imaging parameters and normalized to the same volume of brain tissue. This procedure was be more explicitly described in the Materials & Methods. We have carried out colocalization of Annexin V and excitatory GFP neurons in live organotypic Thy1::GFP slices and have included these data in the figure (**Figure 2b**). There is partial collocalization of Annexin V with* Thy1*::GFP; however, as only 2-3% of neurons in this model express GFP, there also is Annexin V signal outside of GFP.*

Fig. 1: a characterization of the Xkr8 KO mouse should be provided. Is Xkr4 normally expressed? Are TMEM proteins normally expressed?

Authors' response: *We have now assessed levels of Xkr4 and Ano6 (encoding TMEM16F) mRNA in* Xkr8 *cKO and wild-type control mice (**Figure EV3c-e**). There was no effect on Ano6*

*expression and Xkr4 expression was only marginally increased. As Xkr8 is expressed at approximately 8x higher mRNA levels than Xkr4 at P0 in wild-type animals (**Figure EV1b**), such increase cannot compensate the loss of Xkr8 in* Xkr8 *cKO animals.*

Fig. 2A: the boutons should be labeled with both pre and postsynaptic markers to confirm that they are in fact synaptic terminals.

Authors' response: *As the density of axonal varicosities and dendritic spines was unchanged in* Xkr8 *cKO mice (**Figure 3a-e**) we did not perform further validation studies on our samples to determine whether varicosities and spines contained functional synaptic machinery (e.g. vGluT1, PSD95). We will rephrase the text to be more precise in referring to these structures as axonal varicosities and dendritic spines.*

Fig. 2B: the fact that the reduction of bouton size is the consequence of a reduction in the efficiency of synaptic trogocytosis is speculative and would require a direct demonstration. Also, the authors should underline and discuss the evidence that the reduction in the bouton size is detectable only at P28, while Xkr8 is expressed at P0 and drastically downregulated already at P7.

Authors' response: *We agree that a role for trogocytosis in the differences in axonal varicosity size at P28 (**Figure 3b**) remains speculative – we were careful to label it as such in the text – and we have removed this statement as it does not contribute significantly to the findings. Instead we have added a short discussion at the end of the manuscript to point out that this difference appears gradually as axons develop in the absence of Xkr8, and that this indicates either that Xkr8 has additional functions beyond those we describe that persist despite a decrease in its expression during the second and third postnatal weeks, or that its role during the perinatal period leads to a different developmental projectory in which varicosity size is no longer adjusted as it is in wild-type animals.*

Fig. 2D: the authors should analyze the dendritic spines by differentiating them in mature versus immature (mushroom versus filopodia). In the absence of these analyses and without a staining for PSD95, which labels functionally mature spines, it is almost impossible to state that there is "relatively undisturbed maturation of excitatory synapses in Xkr8 cKO mice".

Authors' response: *We believe our results can be used to make a general statement about an absence of differences in spine numbers. A more detailed analysis of spine shape would constitute a major effort and many months of work, given the many timepoints we investigated. Moreover, we are not convinced that any resulting changes in the shape of spines would allow us to draw concrete conclusions about the function of Xkr8, especially as such shape changes could easily result secondarily from changes in the connectivity of the circuitry. We have rephrased the text to more precisely describe the findings of an absence of changes in spine number avoiding more speculative statements about synaptic maturation.*

Fig. 2G and J show that Xkr8 cKO mice display increased density of presynaptic puncta. The authors should quantify the engulfed synaptic material in glial cells to demonstrate whether this

increase results from defective synapse elimination. A quantification of the postsynaptic marker PSD-95 would also be useful to discriminate between presynaptic and postsynaptic involvement.

Authors' response: *We have now quantified internalized SMI312+ axonal material, vGluT1+ pre-synaptic and PSD95+ post-synaptic material within Iba1+ microglia to evaluate whether the excess of axons and their varicosities in* Xkr8 *cKO mice is related to defective pruning of developing axons by microglia (**Figure 4**). We found that loss of Xkr8 significantly reduced microglial uptake of SMI312+ and vGluT1+ axonal material, but not PSD95+ pre-synaptic material. In our study, we did not find any evidence that the density of dendritic spines and axonal varicosities on individual neurons is affected by* Xkr8 *cKO, in contrast to the finding that the number of axons is increased. Therefore, the major finding of this manuscript is that Xkr8 is required for the removal of axons. We have carefully revised the text to avoid any implications of synaptic rather than axonal pruning, as only the latter was observed in our study.*

The sentence "In the absence of a change in bouton density along individual axons, such an increase in the absolute density of excitatory terminals points to a deficiency in the elimination of entire axon arbors, rather than the removal of individual boutons" is rather speculative, given that the boutons in 2A are not demonstrated to be true synapses. In the absence of a clear identification of the structures which expose PtdSer (see fig. 1H), and without analyzing the nature of the engulfed material in glial cells, statements concerning whether isolated synapses or entire axons are engulfed are not supported by data.

Authors' response: *We agree with the reviewer that these are important questions to address. We have added data showing that all axonal varicosities at this developmental stage colocalize with vGluT1 to validate our quantification of varicosities as surrogates for synaptic specializations (**Figure EV3f**). Moreover, we have added data showing that PtdSer is preferentially exposed on axonal varicosities and dendritic spines (colocalization of Annexin V and Thy1::GFP labeled neurons in live organotypic cultures, **Figure EV5**). We believe that our data demonstrating increased axonal density in corpus callosum (**Figure 5**) confirms our hypothesis that* Xkr8 *cKO have impaired axonal elimination. We have also added data demonstrating that lack of Xkr8 leads to increased density of corticospinal axons in the medulla (**Figure 5b**). We have revised the identified sentence to indicate clearly that our findings of the absence of a change in bouton density along individual axons and an increase in the absolute density of excitatory terminals guided our further analysis of axonal densities in corpus callosum and medulla rather than allowing us to state a definite conclusion.*

Fig. 2K: the authors should explain why a clear effect is also detectable in thalamic vGlut2 positive boutons, which express Xkr8.

Authors' response: *The effect of* Emx1*::Cre-driven* Xkr8 *cKO on vGlut2+ axons in cortex suggest that PtdSer-exposure may act in a cell non-autonomous manner to influence neuronal maturation. Interestingly, exposed PtdSer has been shown to be cleaved by phosholipases to generate the chemotactic signal lysoPtdSer and we speculate that a lack of such a cell non-autonomous chemotactic role of PtdSer may explain the phenotypes we see beyond vGluT1+ synapses in our mice. We have now included this point in the revised manuscript.*

Fig. 4: The authors analyze spontaneous and evoked activity in CA1 pyramidal neurons in hippocampal slices from Xkr8 cKO and wild-type control mice at P40 and find significant increase in the amplitude of spontaneous, but not evoked, events. Since spontaneous activity reflects the general circuitry organization, including inhibitory afferents, analyses of the frequency and amplitude of miniature events would be crucial to better disentangle synaptic events. The analysis should be performed also at early postnatal stages, when Xkr8 is highly expressed.

Authors' response: *While we agree that a full description of electrophysiological changes across development in Xkr8 cKO mice would be a valid endeavour and might help determine the evolution of the deficits we see in adulthood, we do not think that such a description would alter significantly our finding that disrupting PtdSer exposure results in a transient excess of presynaptic structures that nevertheless has a long-term impact on neuronal function. Both the electrophysiological and fMRI characterization were intentionally carried out in adult animals with the intention of determining whether early PtdSer-dependent remodeling had any long-term impact on brain function. As we are not in a position to make precise hypotheses about the precise electrophysiological changes we expected under such circumstances we feel the electrophysiological findings stand on their own and would not benefit from a wider description. Our main point was to show that the transient changes in axons we see in the early postnatal period are associated with long-term changes in synaptic connectivity that persist throughout life.*

The reasons put forward to explain the lack of differences in evoked events (page 10, lines 8-13) should be more clearly expressed.

Authors' response: *We apologize for the insufficient clarity and have expressed our reasoning more extensively in the revised manuscript.*

The discussion is very speculative.

Authors' response: *We have eliminated the more speculative statements in the discussion to hone our considerations more closely to the data.*

Minor points
Fig. 1A display a quite high data variability. Did the authors separately analyze males and females?

Authors' response: *Our data set of* ***Figures 1-3*** *included similar numbers of male and female mice. Although the study was not designed to explicitly assess the sex effect in Xkr8-dependent developmental pruning, we have now added a statistical analysis (****Table EV1****) of sex effects in the revised manuscript. Importantly, due to small sample size in separate sex groups sex differences only indicate possible tendencies and should be interpreted with caution. We clearly stated this in the revised manuscript.*

Fig. 3D: a higher number of observations would be required to unequivocally exclude the lack of effect in S2.

Authors' response: *We agree with the reviewer that we cannot rule out a significant effect in S2 and we have revised the text to indicate that, although the difference is larger in S1 than S2, we cannot rule out a statistically significant difference in S2.*

The authors could calculate whether lack of Xkr8 affects synaptic multiplicity, i.e. the density of axon terminals onto a given postsynaptic neuron.

Authors' response: *Inferring synaptic multiplicity would require sequential measurements of mEPSCs and sEPSCs which we have not done in this study. We refer to our rebuttal above as to why we feel that a more extensive electrophysiological characterization of* Xkr8 *cKO mice would not significantly strengthen our major findings.*

Dear Cornelius,

Thank you for submitting your manuscript to The EMBO Journal. Your study has now been seen by the two referees and their comments are provided below. The referees appreciate the introduced revisions with referee #2 having a few remaining points. I have discussed the points raised by referee #2 further with referee #1 and here are our response:

Point regarding to:

Fig. 2 and EV3b
Please provide the statistical analysis and quantification.

Fig. 3A and D:
If you have the requested stainings on hand please include them otherwise OK to respond to this issue in the point-by-point response. Do you have quantification of synaptic PSD95 density in the Xkr8 KO hippocampus? Please also make sure that you show representative images.

Fig. 4:
Please clarify => What does "internalized SMI312, vGlut1, PSD95" on the Y axis represent? Is it volume? Pixels? graphs the number of mice and not only the cells.
Regarding the point of synaptic markers are in fact engulfed in microglia - Can you show that the "engulfed" particles are surrounded by microglia => reanalysis of your microscopy images to show that your IF image analysis shows internalization.

Regarding the last point if Xkr8- dependent PtdSer exposure promotes elimination of entire axons rather than individual synaptic structures. Please make sure you have a balanced discussion about this.

When you submit the revised version will you also take care of the following issues:

- please upload production ready figures

- The funders inserted in the comments box in the online submission system should also be listed and mapped to authors. Please make sure that this info is also in the MS file as well.

- Check the reference style => should be max 10 authors followed by et al.

- COI needs to be re-labelled as Disclosure and competing interests statement

- Please remove the Authors Contributions from the manuscript. The 'Author Contributions' section is replaced by the CRediT contributor roles taxonomy to specify the contributions of each author in the journal submission system. Please use the free text box in the 'author information' section of the manuscript submisssion system to provide more detailed descriptions (e.g., 'X provided intracellular Ca++ measurements in fig Y')

- Please check that there are figure callouts for Figure 1B, Figure 6B-E; Figure EV2A-B, Figure EV4A-B, Figure EV4A and C, Figure EV7A-E, Figure EV8A-D, Figure EV9A-D, Figure EV10A.

- You currently have 10 EV figures, but can only have 5. The rest can be added to the appendix. The appendix should have a ToC. Please also see our guide to authors for proper nomenclature and figure callout for the appendix figures.

- Please upload a synopsis text. We need a summary statement plus 3-5 bullet points describing the key findings of the MS.

- We also need a synopsis image => 550 wide by [200-400]

- Table EV1 should be renamed to Table 1 with the corresponding callout or uploaded separately as Table EV1

- Section order should be corrected

- Author email bounced for: Senthilkumar Deivasigamani, Bernadette Basilico, Aditya Bali

- Our publisher has also done their pre-publication check on your manuscript. When you log into the manuscript submission system you will see the file "Data Edited Manuscript file". Please take a look at the word file and the comments regarding the figure legends and respond to the issues.

- Also, our source data coordinate sent you a list of figure panels where we would need source data for back in August. I have attached the list again. Please provide source data for the figures when you submit your revised version.

That should be all - let me know if we need to discuss anything further

Best Karin

Karin Dumstrei, PhD
Senior Editor
The EMBO Journal

Guide For Authors: https://www.embopress.org/page/journal/14602075/authorguide

----

Referee #1:

The authors have addressed all of this reviewer's concerns, which greatly improves the impact of this work.

A very minor point: regarding the question EMX1 driver, the point is sufficiently made about lack of Xkr8 expression in glia. In terms of the statement about the timing of cortical gliogenesis - the gliogenic switch occurs perinatally potentially in time for the phenotypes observed, so the EMX1 driver for other targets could very well influence glia-specific processes in the perinatal period (see Kessaris et al, Nat Neurosci, 2006 for OPCs example).

Referee #2:

The revision performed by the Authors is partially satisfactory.

Fig. 2 and EV3b: the representative pictures in EV3b show a relevant reduction of active caspase 3 in XkR8 KO. A significant decrease in caspase 3 at P15 is also evident from the graph in Fig. 2c. Is statistical analysis missing? Further, it is not clear how the mean fluorescence in Fig.2 was analyzed. Only annexin colocalized with GFP positive neurons should be quantified.

Fig. 3A and D: a staining with pre- and post- synaptic markers is required to determine the level of maturation of these structures in the Xkr8 KO model with respect to the WT. Further, since the Authors assessed the amount of PSD95 internalized inside microglia (fig.4), a parallel quantification of synaptic PSD95 density in the Xkr8 KO hippocampus is mandatory. A synaptic staining would also help in Fig. EV5b and d. Here, the images are not representative, neither show a higher exposure of PtdSer on boutons or dendritic spines compared to shafts.

Fig. 4: in the absence of lysosomal staining (e.g. CD68) it is hard to state that synaptic markers are in fact engulfed in microglia. Why didn't the authors use the 3D reconstruction already exploited for the localization of phosphatidylserine and the dendritic spines? What does "internalized SMI312, vGlut1, PSD95" on the Y axis represent? Is it volume? Pixels? Authors should also show in their graphs the number of mice and not only the cells.

The idea that the Xkr8- dependent PtdSer exposure promotes elimination of entire axons rather than individual synaptic structures is still fully speculative.

Dear Dr. Dumstrei,

Here is our point-by-point response to the Referees:

Referee #1:

The authors have addressed all of this reviewer's concerns, which greatly improves the impact of this work.

A very minor point: regarding the question EMX1 driver, the point is sufficiently made about lack of Xkr8 expression in glia. In terms of the statement about the timing of cortical gliogenesis - the gliogenic switch occurs perinatally potentially in time for the phenotypes observed, so the EMX1 driver for other targets could very well influence glia-specific processes in the perinatal period (see Kessaris et al, Nat Neurosci, 2006 forOPCs example).

We thank the reviewer for this important note and have revised the manuscript to remove the statement regarding the development of astrocytes and oligodendrocytes.

****

Referee #2:

The revision performed by the Authors is partially satisfactory.

Fig. 2 and EV3b: the representative pictures in EV3b show a relevant reduction of active caspase 3 in XkR8 KO. A significant decrease in caspase 3 at P15 is also evident from the graph in Fig. 2c. Is statistical analysis missing? Further, it is not clear how the mean fluorescence in Fig.2 was analyzed. Only annexin colocalized with GFP positive neurons should be quantified.

We apologize for insufficient clarity regarding the analysis of active caspase-3. As the referee noted, there is an apparent reduction of active caspase-3 that was observed both in representative images (Figure EV3B) as well as signal quantification (Figure 2C). However, two-way ANOVA did not indicate any significant effect of either genotype or age ($F(2,30) = 0.86$, $p = 0.433$). For clarity, we have now added the output of ANOVA analysis to the legend of Figure 2. In addition, we have indicated in the legend of Figure EV3 that the quantification of the signal is presented in Figure 2C to ease the linking these two panels together.

We apologize for omitting the details regarding the quantification of fluorescent signal of annexin V in Figure 2B. We have now added explicit description of annexin V binding quantification in the Methods section. As Thy1*::GFP labels only small fraction of neurons (2-3%), we chose to analyze annexin V binding throughout the organotypic section in order not to exclude the majority of annexin V signal. In the revised manuscript, we indicated that the lack of Xkr8 leads to the reduction of PtdSer exposure in the brain tissue rather than on neurons. However, as Xkr8 is mostly observed in neurons (Figure 1, Figure EV1, Appendix Figure S1) and we used* Emx1*::Cre line to knock-out Xkr8, the reduction of annexin V binding in the brain tissue is most likely linked to reduced neuronal PtdSer exposure.*

Fig. 3A and D: a staining with pre- and post- synaptic markers is required to determine the level of maturation of these structures in the Xkr8 KO model with respect to the WT. Further, since the Authors assessed the amount of PSD95 internalized inside microglia (fig.4), a parallel quantification of synaptic PSD95 density in the Xkr8 KO hippocampus is mandatory. A synaptic staining would also help in Fig. EV5b and d. Here, the images are not representative, neither show a higher exposure of PtdSer on boutons or dendritic spines compared to shafts.

We agree with the reviewer that synaptic markers would be necessary to evaluate structural maturation of pre- and post- synapses. We have found that all axonal varicosities are immunopositive for vGlut1+ (Figure EV2F). However, we found no indication that the lack of Xkr8 affects the post-synaptic compartment: dendritic spine density was normal (Figure 3D, E) as was dendritic arbour length (Figure 3F) or branching (Figure EV4). Furthermore, the analysis of microglial uptake indicated no differences in the internalization of PSD95 structures (Figure 4E, F). Therefore, we did not pursue further analysis of post-synaptic compartment in Xkr8 *cKO mice. To avoid any unsupported statements any references regarding the structural maturation of pre- or post-synapses had been removed in the previous revision.*

We apologize for inappropriate choice of images in Figure EV5. We have now replaced them with more representative images and added arrows to mark annexin V signal on axonal boutons and dendritic spines.

Fig. 4: in the absence of lysosomal staining (e.g. CD68) it is hard to state that synaptic markers are in fact engulfed in microglia. Why didn't the authors use the 3D reconstruction already exploited for the localization of phosphatidylserine and the dendritic spines? What does "internalized SMI312, vGlut1, PSD95" on the Y axis represent? Is it volume? Pixels? Authors should also show in their graphs the number of mice and not only the cells.

We apologize for insufficient clarity in describing the analysis of microglial internalization of neuronal material. We have indeed performed the analysis in 3D, but open access software did not visualize it properly. We have now added 3D reconstruction on IMARIS to demonstrate that only the signal within Iba1+ microglial body volume was analyzed. We also apologize for inappropriate presentation of quantitative results. We have now changed the Y axis to "SMI312/vGluT1/PSD95 in 3D Iba1+ microglia" and have specified in the legend of Figure 4 that the total volume of internalized particles was normalized to microglial cell volume. We have also changed the representation of data in the graphs as mice rather than individual microglial cells. As nested design ANOVA was used to compare Xkr8 *WT and* Xkr8 *cKO samples, the* p *values remained the same.*

The idea that the Xkr8- dependent PtdSer exposure promotes elimination of entire axons rather than individual synaptic structures is still fully speculative.

Having identified reduced axonal densities in corticospinal tract (Figure 5B) and corpus callosum (Figure 5E) as well as increased density of vGluT1+ particles (Figure 3J) in the absence of changes of bouton density on a single axon (Figure 3C) in Xkr8 *cKO mice, we concluded that such differences can only be a result of the removal of whole axons. However, the referee is correct to point out that there may be other mechanisms of axonal refinement that*

involve Xkr8 in addition to PtdSer exposure. For example, Xkr8 has been demonstrated to interact with extracellular matrix metalloproteinase inducer Basigin or synaptic glycoprotein neuroplastin (Sakuragi et al., 2021; doi: 10.1038/s41594-021-00665-8), which could in turn modulate neurodevelopment through the remodelling of extracellular matrix or by regulating calcium homeostasis. We have now expanded the discussion to include these alternatives.

****

Changes to Figures:

- Figure 4 now includes 3D reconstruction of IMARIS to depict internalized axonal and dendritic material better.
- Images in Appendix Figure S2 are now changed to more representative images and annexin V on axonal varicosities and dendritic spines is indicated with arrows.
- While preparing the Source Data, we have noticed some inaccuracies that have now been corrected:
    - Figure 1C P0 WT image and adult cKO image as well as Figure 1E adult cKO images were updated.
    - DAPI image in the top row of Figure EV3 was updated.
    - The scale of the graph of Figure EV5B has been corrected.

Other changes:

- Production ready tiff files of figures have been uploaded.
- All funders have been listed and mapped to authors except for L'Oreal-UNESCO For Women in Science programme, which was not available in the list of possible funders.
- The reference style has been updated.
- COI was re-labelled as "Disclosure and competing interests statement".
- Authors Contributions have been removed from the manuscript, detailed descriptions have been provided in the section with authors information. Authors' emails have been updated.
- The callouts for each figure have been included.
- Five EV figures (Figures EV1-EV5) have been prepared, the remaining five figures (Appendix Figures S1-S5) and the table (Appendix Table S1) have been moved to the appendix.
- Synopsis image and text have been uploaded.
- Section order has been corrected.
- All issues in a data edited manuscript file have been resolved.
- Source Data files have been prepared. Numerical data files for main figures (Figure 1-7) have been uploaded and linked to the corresponding figures. Numerical data files for extended view and appendix have been uploaded as a zip folder. Microscopy images have been uploaded on BioImage (accession No. S-BIAD678)

2nd Revision - Editorial Decision 25th Apr 2023

Dear Cornelius and Urte,

Thank you for submitting your revised manuscript to the EMBO Journal. I have now had a chance to take a look at it and I appreciate the introduced changes.

I am therefore very pleased to accept the MS for publication here.

With best wishes

Karin

Karin Dumstrei, PhD
Senior Editor
The EMBO Journal

Please note that it is EMBO Journal policy for the transcript of the editorial process (containing referee reports and your response letter) to be published as an online supplement to each paper. If you do NOT want this, you will need to inform the Editorial Office via email immediately. More information is available here: https://www.embopress.org/page/journal/14602075/authorguide#transparentprocess

Your manuscript will be processed for publication in the journal by EMBO Press. Manuscripts in the PDF and electronic editions of The EMBO Journal will be copy edited, and you will be provided with page proofs prior to publication. Please note that supplementary information is not included in the proofs.

You will be contacted by Wiley Author Services to complete licensing and payment information. The required 'Page Charges Authorization Form' is available here: https://www.embopress.org/pb-assets/embo-site/tej_apc.pdf - please download and complete the form and return to embopressproduction@wiley.com

EMBO Press participates in many Publish and Read agreements that allow authors to publish Open Access with reduced/no publication charges. Check your eligibility: https://authorservices.wiley.com/author-resources/Journal-Authors/open-access/affiliation-policies-payments/index.html

Should you be planning a Press Release on your article, please get in contact with embojournal@wiley.com as early as possible, in order to coordinate publication and release dates.

If you have any questions, please do not hesitate to call or email the Editorial Office. Thank you for your contribution to The EMBO Journal.

EMBO Press Author Checklist

Corresponding Author Name: Cornelius Gross
Journal Submitted to: EMBO Journal
Manuscript Number: EMBOJ-2022-111790

USEFUL LINKS FOR COMPLETING THIS FORM
The EMBO Journal - Author Guidelines
EMBO Reports - Author Guidelines
Molecular Systems Biology - Author Guidelines
EMBO Molecular Medicine - Author Guidelines

Reporting Checklist for Life Science Articles (updated January
This checklist is adapted from Materials Design Analysis Reporting (MDAR) Checklist for Authors. MDAR establishes a minimum set of requirements in transparent reporting in the life sciences (see Statement of Task: 10.31222/osf.io/9sm4x). Please follow the journal's guidelines in preparing your
Please note that a copy of this checklist will be published alongside your article.

Abridged guidelines for figures
1. Data
The data shown in figures should satisfy the following conditions:
- the data were obtained and processed according to the field's best practice and are presented to reflect the results of the experiments in an accurate and unbiased manner.
- ideally, figure panels should include only measurements that are directly comparable to each other and obtained with the same assay.
- plots include clearly labeled error bars for independent experiments and sample sizes. Unless justified, error bars should not be shown for technical
- if n<5, the individual data points from each experiment should be plotted. Any statistical test employed should be justified.
- Source Data should be included to report the data underlying figures according to the guidelines set out in the authorship guidelines on Data

2. Captions
Each figure caption should contain the following information, for each panel where they are relevant:
- a specification of the experimental system investigated (eg cell line, species name).
- the assay(s) and method(s) used to carry out the reported observations and measurements.
- an explicit mention of the biological and chemical entity(ies) that are being measured.
- an explicit mention of the biological and chemical entity(ies) that are altered/varied/perturbed in a controlled manner.
- the exact sample size (n) for each experimental group/condition, given as a number, not a range;
- a description of the sample collection allowing the reader to understand whether the samples represent technical or biological replicates (including how many animals, litters, cultures, etc.).
- a statement of how many times the experiment shown was independently replicated in the laboratory.
- definitions of statistical methods and measures:
  - common tests, such as t-test (please specify whether paired vs. unpaired), simple χ2 tests, Wilcoxon and Mann-Whitney tests, can be unambiguously identified by name only, but more complex techniques should be described in the methods section;
  - are tests one-sided or two-sided?
  - are there adjustments for multiple comparisons?
  - exact statistical test results, e.g., P values = x but not P values < x;
  - definition of 'center values' as median or average;
  - definition of error bars as s.d. or s.e.m.

Please complete ALL of the questions below.
Select "Not Applicable" only when the requested information is not relevant for your study.

Materials

Newly Created Materials	Information included in the manuscript?	In which section is the information available? (Reagents and Tools Table, Materials and Methods, Figures, Data Availability Section)
New materials and reagents need to be available; do any restrictions apply?	Not Applicable	

Antibodies	Information included in the manuscript?	In which section is the information available? (Reagents and Tools Table, Materials and Methods, Figures, Data Availability Section)
For **antibodies** provide the following information:<br>- Commercial antibodies: RRID (if possible) or supplier name, catalogue number and or/clone number<br>- Non-commercial: RRID or citation	Yes	Materials and Methods

DNA and RNA sequences	Information included in the manuscript?	In which section is the information available? (Reagents and Tools Table, Materials and Methods, Figures, Data Availability Section)
**Short novel DNA or RNA including primers, probes**: provide the sequences.	Yes	Materials and Methods

Cell materials	Information included in the manuscript?	In which section is the information available? (Reagents and Tools Table, Materials and Methods, Figures, Data Availability Section)
**Cell lines:** Provide species information, strain. Provide accession number in repository **OR** supplier name, catalog number, clone number, and/**OR** RRID.	Not Applicable	
**Primary cultures:** Provide species, strain, sex of origin, genetic modification status.	Yes	Materials and Methods, Figures
Report if the cell lines were recently **authenticated** (e.g., by STR profiling) and tested for mycoplasma contamination.	Not Applicable	

Experimental animals	Information included in the manuscript?	In which section is the information available? (Reagents and Tools Table, Materials and Methods, Figures, Data Availability Section)
**Laboratory animals or Model organisms:** Provide species, strain, sex, age, genetic modification status. Provide accession number in repository **OR** supplier name, catalog number, clone number, **OR** RRID.	Yes	Materials and Methods
**Animal observed in or captured from the field:** Provide species, sex, and age where possible.	Not Applicable	
Please detail **housing and husbandry conditions**.	Yes	Materials and Methods

Plants and microbes	Information included in the manuscript?	In which section is the information available? (Reagents and Tools Table, Materials and Methods, Figures, Data Availability Section)
**Plants:** provide species and strain, ecotype and cultivar where relevant, unique accession number if available, and source (including location for collected wild specimens).	Not Applicable	
**Microbes:** provide species and strain, unique accession number if available, and source.	Not Applicable	

Human research participants	Information included in the manuscript?	In which section is the information available? (Reagents and Tools Table, Materials and Methods, Figures, Data Availability Section)
If collected and within the bounds of privacy constraints report on age, sex and gender or ethnicity for all study participants.	Not Applicable	

Core facilities	Information included in the manuscript?	In which section is the information available? (Reagents and Tools Table, Materials and Methods, Figures, Data Availability Section)
If your work benefited from core facilities, was their service mentioned in the acknowledgments section?	Yes	Acknowledgements

Design

Study protocol	Information included in the manuscript?	In which section is the information available? (Reagents and Tools Table, Materials and Methods, Figures, Data Availability Section)

If study protocol has been **pre-registered, provide DOI in the manuscript**. For clinical trials, provide the trial registration number **OR** cite DOI.	Not Applicable	
Report the **clinical trial registration number** (at ClinicalTrials.gov or equivalent), where applicable.	Not Applicable	

Laboratory protocol	Information included in the manuscript?	In which section is the information available? (Reagents and Tools Table, Materials and Methods, Figures, Data Availability Section)
Provide DOI OR other citation details if **external detailed step-by-step protocols** are available.	Not Applicable	

Experimental study design and statistics	Information included in the manuscript?	In which section is the information available? (Reagents and Tools Table, Materials and Methods, Figures, Data Availability Section)
Include a statement about **sample size** estimate even if no statistical methods were used.	Yes	Materials and Methods, Figures
Were any steps taken to minimize the effects of subjective bias when allocating animals/samples to treatment (e.g. **randomization procedure**)? If yes, have they been described?	Not Applicable	
Include a statement about **blinding** even if no blinding was done.	Yes	Materials and Methods
Describe **inclusion/exclusion criteria** if samples or animals were excluded from the analysis. Were the criteria pre-established?<br><br>If sample or data points were omitted from analysis, report if this was due to attrition or intentional exclusion and provide justification.	Yes	Materials and Methods
For every figure, are **statistical tests** justified as appropriate? Do the data meet the assumptions of the tests (e.g., normal distribution)? Describe any methods used to assess it. Is there an estimate of variation within each group of data? Is the variance similar between the groups that are being statistically compared?	Yes	Materials and Methods

Sample definition and in-laboratory replication	Information included in the manuscript?	In which section is the information available? (Reagents and Tools Table, Materials and Methods, Figures, Data Availability Section)
In the figure legends: state number of times the experiment was **replicated** in laboratory.	Yes	Figures
In the figure legends: define whether data describe **technical or biological replicates**.	Yes	Figures

Ethics

Ethics	Information included in the manuscript?	In which section is the information available? (Reagents and Tools Table, Materials and Methods, Figures, Data Availability Section)
Studies involving **human participants**: State details of **authority granting ethics approval** (IRB or equivalent committee(s), provide reference number for approval.	Not Applicable	
Studies involving **human participants**: Include a statement confirming that **informed consent** was obtained from all subjects and that the experiments conformed to the principles set out in the WMA Declaration of Helsinki and the Department of Health and Human Services Belmont Report.	Not Applicable	
Studies involving **human participants:** For publication of **patient photos**, include a statement confirming that consent to publish was obtained.	Not Applicable	
Studies involving experimental **animals**: State details of **authority granting ethics approval** (IRB or equivalent committee(s), provide reference number for approval. Include a statement of compliance with ethical regulations.	Yes	Materials and Methods
Studies involving **specimen and field samples:** State if relevant **permits** obtained, provide details of authority approving study; if none were required, explain why.	Not Applicable	

Dual Use Research of Concern (DURC)	Information included in the manuscript?	In which section is the information available? (Reagents and Tools Table, Materials and Methods, Figures, Data Availability Section)
Could your study fall under dual use research restrictions? Please check biosecurity documents and list of **select agents and toxins** (CDC): https://www.selectagents.gov/sat/list.htm	Not Applicable	
If you used a select agent, is the security level of the lab appropriate and reported in the manuscript?	Not Applicable	
If a study is subject to dual use research of concern regulations, is the name of the **authority granting approval and reference number** for the regulatory approval provided in the manuscript?	Not Applicable	

Reporting

The MDAR framework recommends adoption of discipline-specific guidelines, established and endorsed through community initiatives. Journals have their own policy about requiring specific guidelines and recommendations to complement MDAR.

Adherence to community standards	Information included in the manuscript?	In which section is the information available? (Reagents and Tools Table, Materials and Methods, Figures, Data Availability Section)
State if relevant guidelines or checklists (e.g., **ICMJE, MIBBI, ARRIVE, PRISMA**) have been followed or provided.	Not Applicable	
For **tumor marker prognostic studies,** we recommend that you follow the **REMARK** reporting guidelines (see link list at top right). See author guidelines, under 'Reporting Guidelines'. Please confirm you have followed these guidelines.	Not Applicable	
For **phase II and III randomized controlled trials**, please refer to the **CONSORT** flow diagram (see link list at top right) and submit the CONSORT checklist (see link list at top right) with your submission. See author guidelines, under 'Reporting Guidelines'. Please confirm you have submitted this list.	Not Applicable	

Data Availability

Data availability	Information included in the manuscript?	In which section is the information available? (Reagents and Tools Table, Materials and Methods, Figures, Data Availability Section)
Have **primary datasets** been deposited according to the journal's guidelines (see 'Data Deposition' section) and the respective accession numbers provided in the Data Availability Section?	Not Applicable	
Were **human clinical and genomic datasets** deposited in a public access-controlled repository in accordance to ethical obligations to the patients and to the applicable consent agreement?	Not Applicable	
Are **computational models** that are central and integral to a study available without restrictions in a machine-readable form? Were the relevant accession numbers or links provided?	Not Applicable	
If publicly available data were reused, provide the respective **data citations in the reference list**.	Not Applicable	
